# Supplementary material for: Synthesis and NLRP3-Inflammasome Inhibitory Activity of the Naturally Occurring Velutone F and of Its Non-Natural Regioisomeric Chalconoids
Source: Int J Mol Sci. 2022 Aug 11;23(16):8957. doi: 10.3390/ijms23168957 (PMC9409317; doi:10.3390/ijms23168957)
Supplement: Supplementary file 1 [file ijms-23-08957-s001.zip › ijms-1839410-supplementary.pdf]

Synthesis and NLRP3-inflammasome inhibitory activity of the naturally occurring Velutone F and of its non-natural regioisomeric chalconoids

Tiziano De Ventura[1$], Mariasole Perrone[2,3$], Sonia Missiroli[2,3], Paolo Pinton[2,3,4], Paolo Marchetti[1], Giovanni Strazzabosco[1], Giulia Turrin[1], Davide Illuminati[1], Virginia Cristofori[1], Anna Fantinati[5], Martina Fabbri[1], Carlotta Giorgi[2,3*], Claudio Trapella[1,3*] and Vinicio Zanirato[1]

[1]Department of Chemistry, Pharmaceutical and Agricultural Sciences. University of Ferrara Via Luigi Borsari 46, 44121 Ferrara, Italy
[2]Department of Medical Sciences, Section of Experimental Medicine. University of Ferrara Via Fossato di Mortara,64/b, 44121 Ferrara, Italy.
[3] Laboratory for Technologies of Advanced Therapies (LTTA) Via Fossato di Mortara,70, 44121 Ferrara, Italy
[4] Maria Cecilia Hospital, GVM Care & Research, 48033 Cotignola, Italy
[5] Department of Environmental and Prevention Sciences, University of Ferrara, Via Fossato di Mortara 17, 44121 Ferrara, Italy.
[*]Corresponding authors
[$]Both the authors contributed equally

Summary

METHYL 3-(4-METHOXY-4-OXOBUTANOYL)FURAN-2-CARBOXYLATE (3) 3

METHYL 3-(1,1,4-TRIMETHOXY-4-OXOBUTYL)FURAN-2-CARBOXYLATE (4) 5

METHYL 4-HYDROXY-7-METHOXYBENZOFURAN-5-CARBOXYLATE (5) 7

METHYL 4,7-DIMETHOXYBENZOFURAN-5-CARBOXYLATE (6) 9

4,7-DIMETHOXYBENZOFURAN-5-CARBALDEHYDE (7) 11

2,5-DIMETHOXYPHENOL (9) 13

4-BROMO-2,5-DIMETHOXYPHENOL (10) 15

1-BROMO-4-(2,2-DIMETHOXYETHOXY)-2,5-DIMETHOXYBENZENE (11) 17

5-BROMO-4,7-DIMETHOXYBENZOFURAN (12) 19

1-PHENYLPROP-2-EN-1-ONE (13) 22

2-(2,5-DIMETHOXYPHENOXY)TETRAHYDRO-2*H*-PYRAN (14) 24

2-HYDROXY-3,6-DIMETHOXYBENZALDEHYDE (15) 24

3-BROMO-6-HYDROXY-2,5-DIMETHOXYBENZALDEHYDE (16) 26

ETHYL 2-(4-BROMO-2-FORMYL-3,6-DIMETHOXYPHENOXY)ACETATE (17) 29

2-(3-BROMO-6-FORMYL-2,5-DIMETHOXYPHENOXY)ACETIC ACID 32

(E)-3-(4,7-DIMETHOXYBENZOFURAN-5-YL)-1-PHENYLPROP-2-EN-1-ONE (1) 35

2-(2,2-DIMETHOXYETHOXY)-1,4-DIMETHOXYBENZENE (18) 40

4,7-DIMETHOXYBENZOFURAN (19) 42

4,7-DIMETHOXYBENZOFURAN-2-CARBALDEHYDE (20) + 4,7-DIMETHOXYBENZOFURAN-5-CARBALDEHYDE (7) 44

4,7-DIMETHOXYBENZOFURAN-6-CARBALDEHYDE (21) 47

1-(2.5-DIMETHOXYPHENOXY)PROPAN-2-ONE (24) 49

4,7-DIMETHOXY-3-METHYLBENZOFURAN (25) 52

(4,7-DIMETHOXYBENZOFURAN-3- YL)METHANOL (26) 55

4,7-DIMETHOXYBENZOFURAN-3-CARBALDEHYDE (27) 58

(*E*)-3-(4,7-DIMETHOXYBENZOFURAN-6-YL)-1-PHENYLPROP-2-EN-1-ONE (23) 61

(*E*)-3-(4,7-DIMETHOXYBENZOFURAN-2-YL)-1-PHENYLPROP-2-EN-1-ONE (22) 68

(*E*)-3-(4,7-DIMETHOXYBENZOFURAN-3-YL)-1-PHENYLPROP-2-EN-1-ONE (28) 73

methyl 3-(4-methoxy-4-oxobutanoyl)furan-2-carboxylate (3)

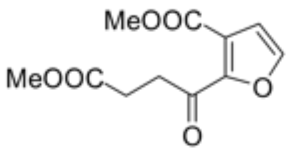

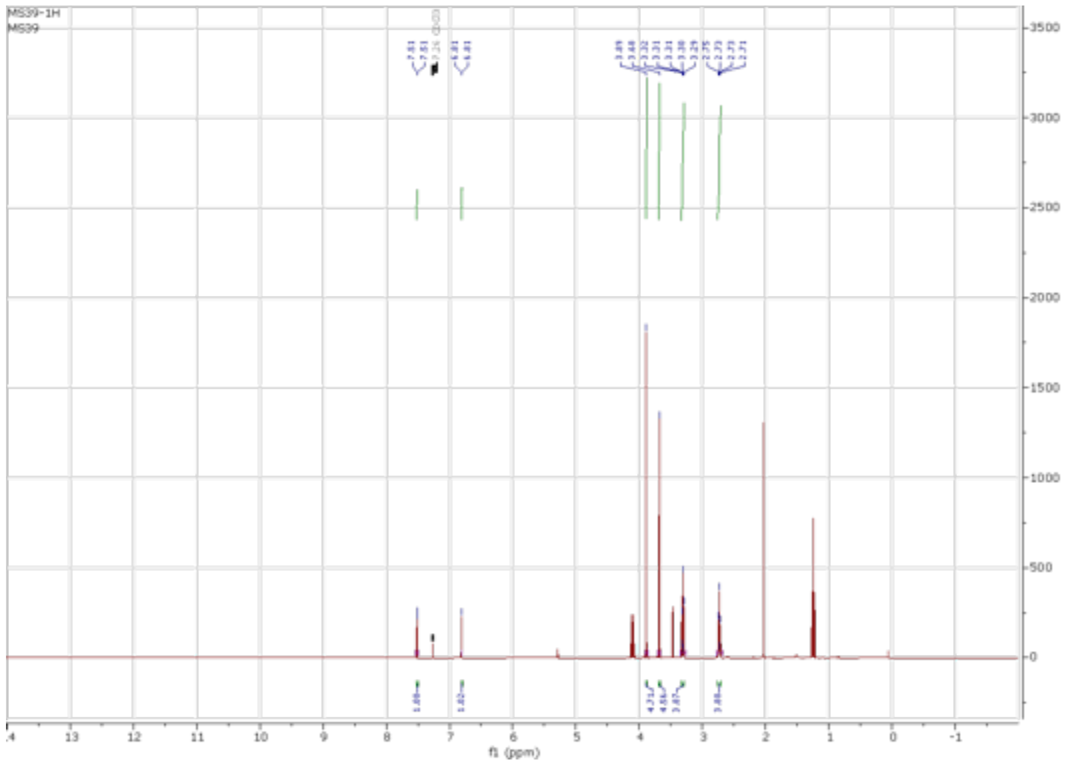

^{1}H NMR (400 MHz, Chloroform-*d*) δ 7.51 (d, *J* = 1.7 Hz, 1H), 6.81 (d, *J* = 1.7 Hz, 1H), 3.89 (s, 3H), 3.68 (s, 3H), 3.31 (t, *J* = 6.7 Hz, 2H), 2.73 (t, *J* = 6.6 Hz, 2H).

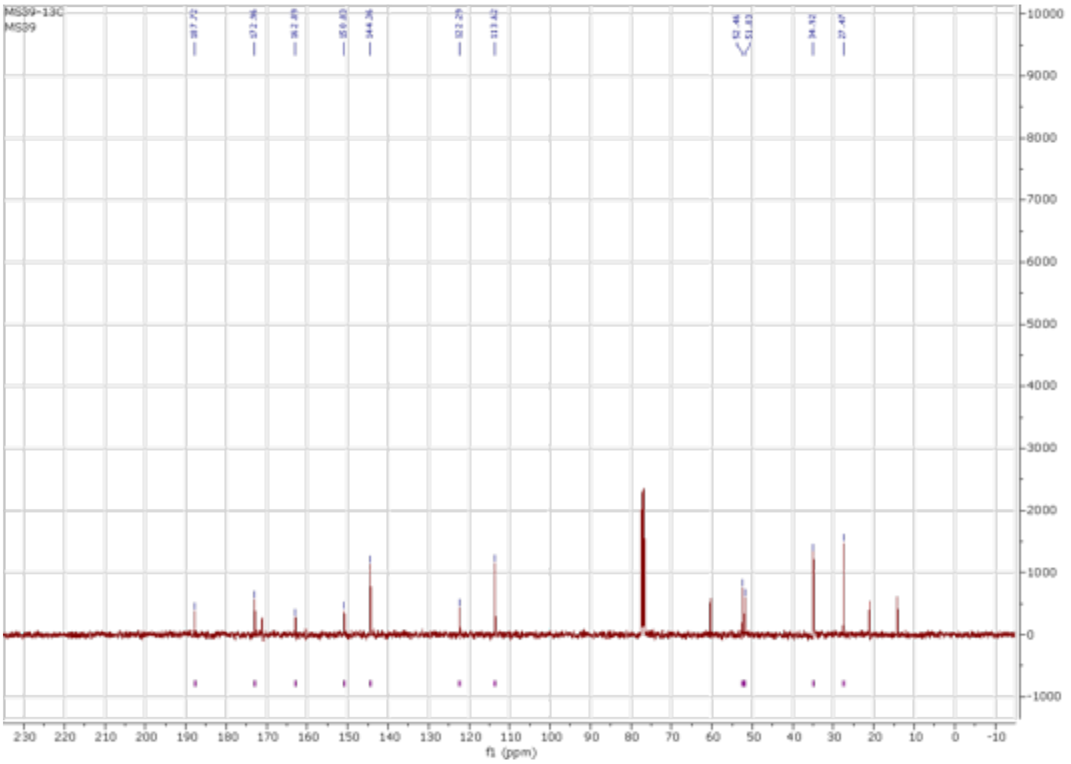

^{13}C NMR (101 MHz, Chloroform-d) δ 187.72, 172.96, 162.89, 150.83, 144.36, 122.29, 113.62, 52.46, 51.83, 34.92, 27.47.

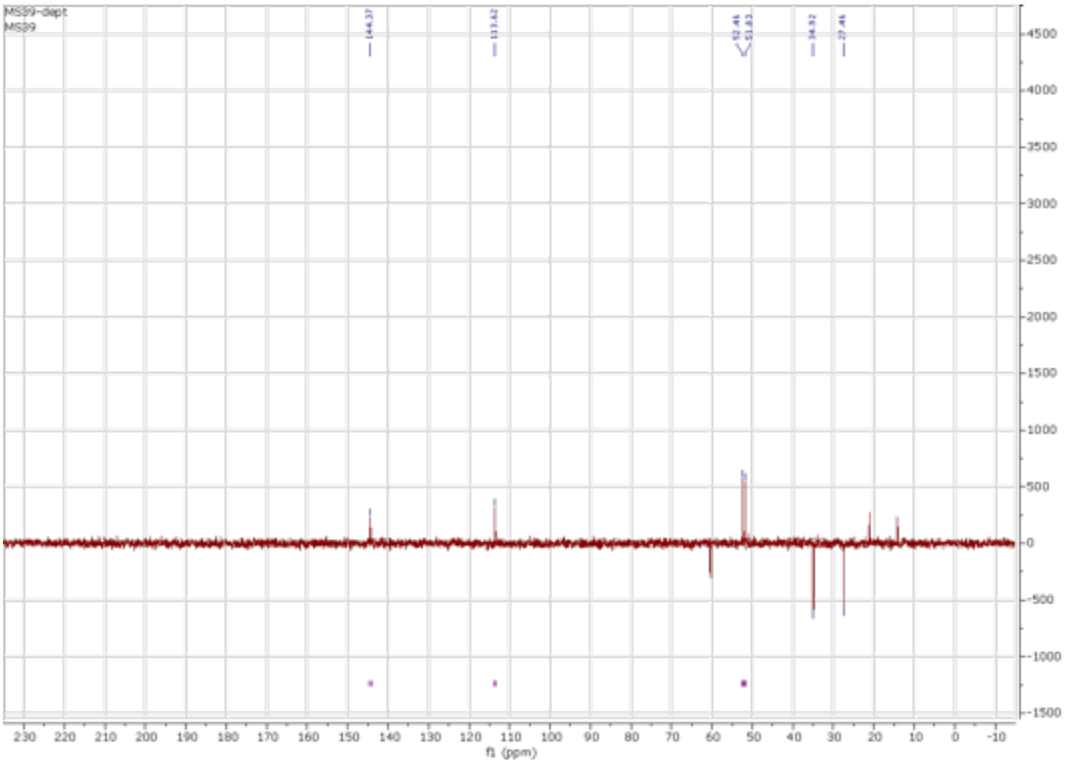

^{13}C NMR (101 MHz, Chloroform-d) δ 144.37, 113.62, 52.46, 51.83, 34.92, 27.46.

methyl 3-(1,1,4-trimethoxy-4-oxobutyl)furan-2-carboxylate **(4)**

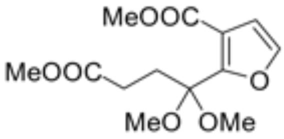

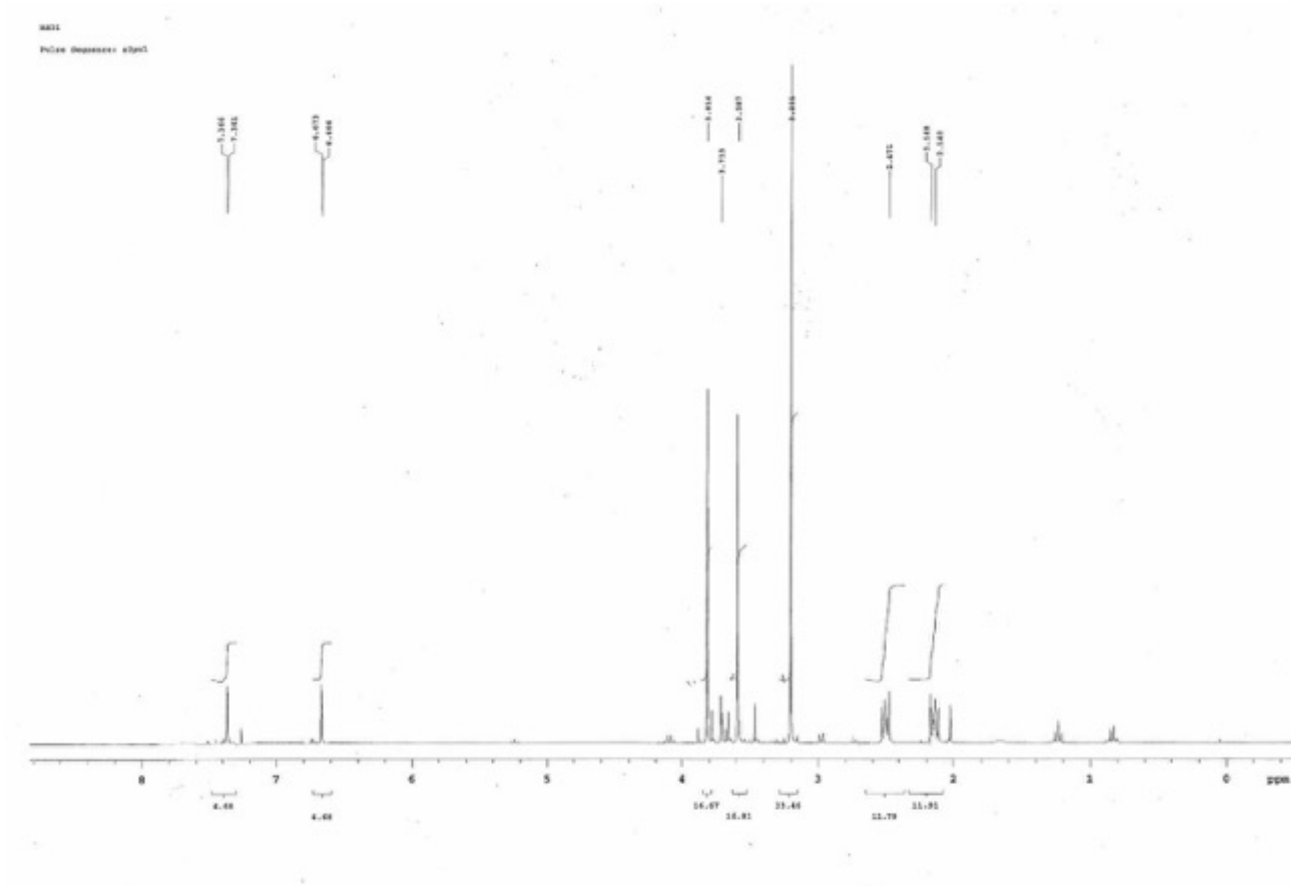

^{1}H NMR (400 MHz, Chloroform-*d*) δ 7.36 (d, *J* = 1.7 Hz, 1H), 6.66 (d, *J* = 1.7 Hz, 1H), 3.81 (s, 3H), 3.58 (s, 3H), 3.20 (s, 6H), 2.47 (t, *J* = 6.7 Hz, 2H), 2.16 (t, *J* = 6.6 Hz, 2H).

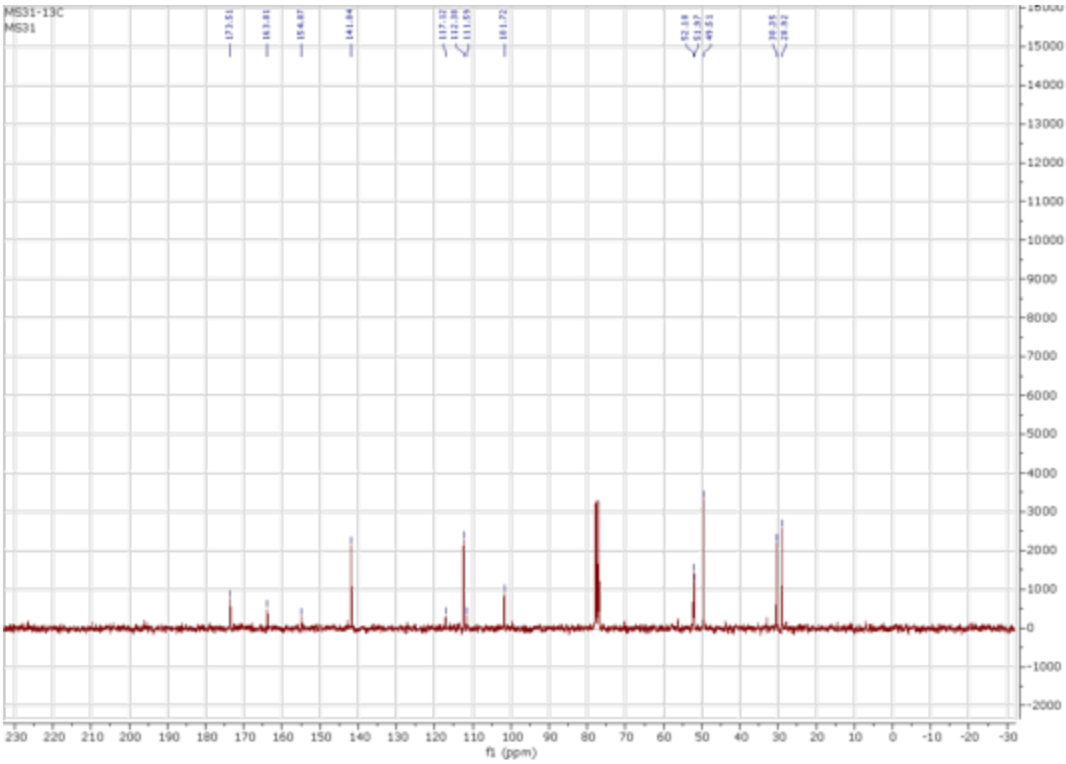

^{13}C NMR (75 MHz, Chloroform-d) δ 173.51, 163.81, 154.87, 141.84, 117.12, 112.38, 111.59, 101.72, 52.18, 51.97, 49.51, 30.35, 28.92.

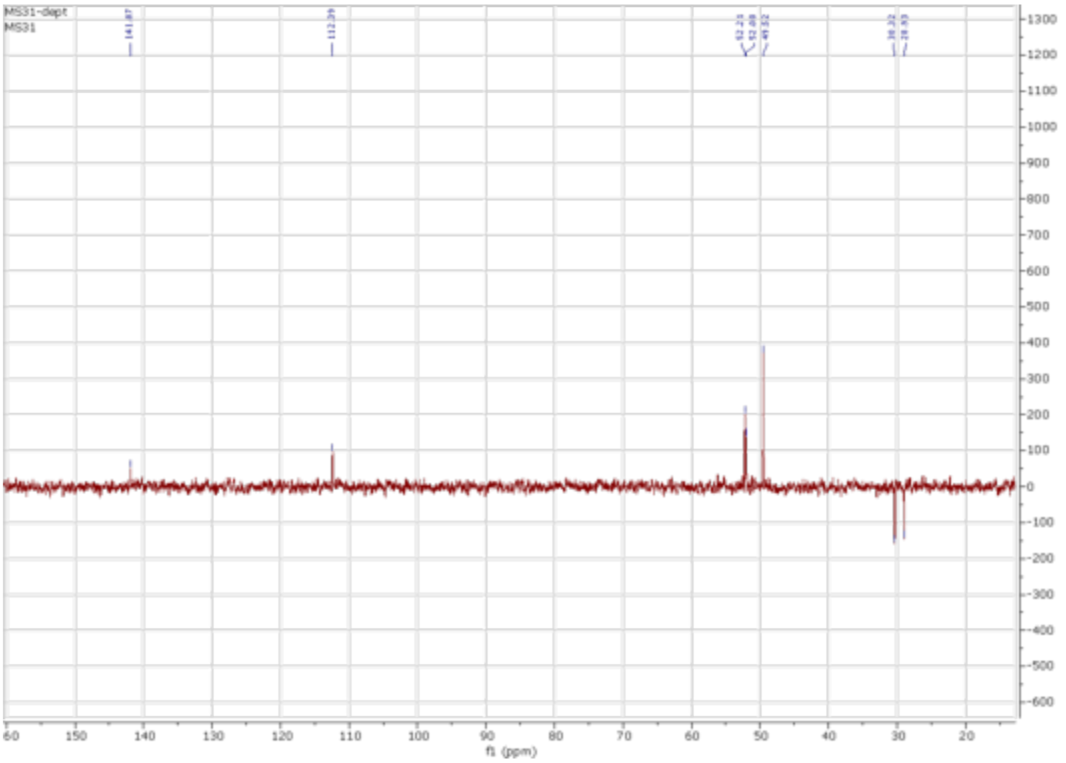

^{13}C NMR (75 MHz, Chloroform-d) δ 141.87, 112.39, 52.21, 52.00, 49.52, 30.32, 28.93.

methyl 4-hydroxy-7-methoxybenzofuran-5-carboxylate (5)

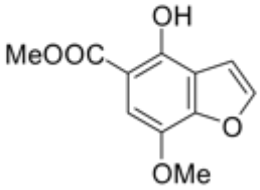

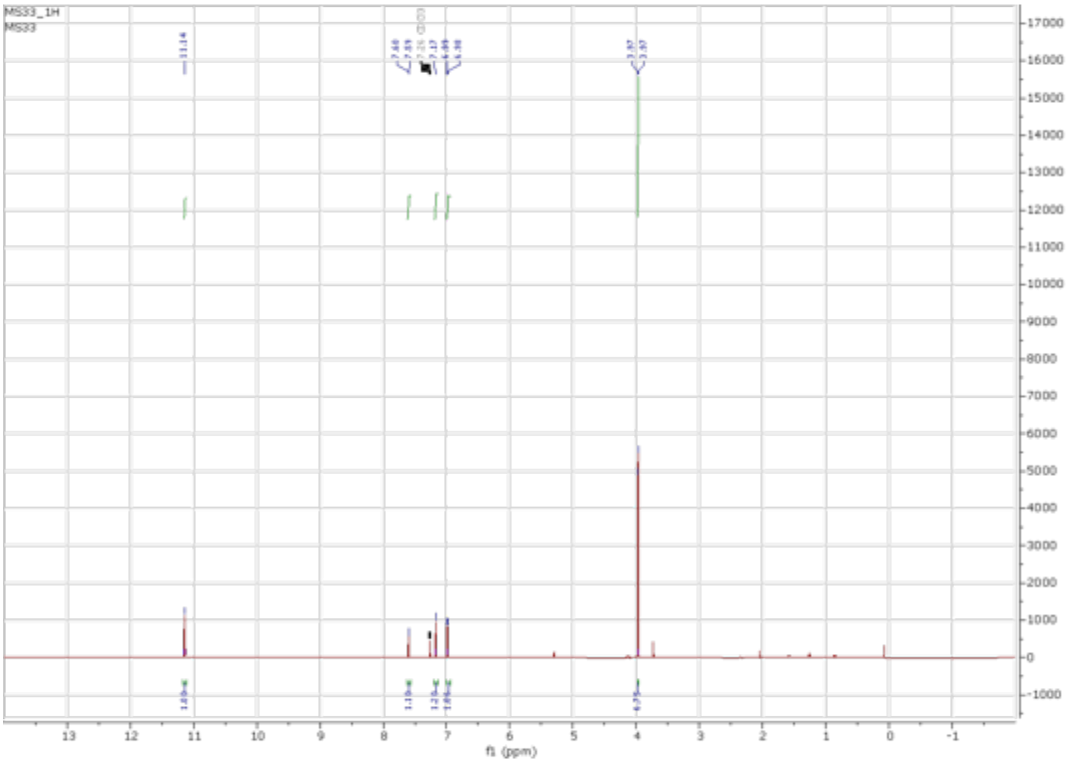

^{1}H NMR (400 MHz, Chloroform-*d*) δ 11.14 (s, 1H), 7.60 (dd, *J* = 2.1 Hz, 1H), 7.17 (s, 1H), 6.98 (d, *J* = 2.1 Hz, 1H), 3.97 (d, *J* = 1.6 Hz, 6H).

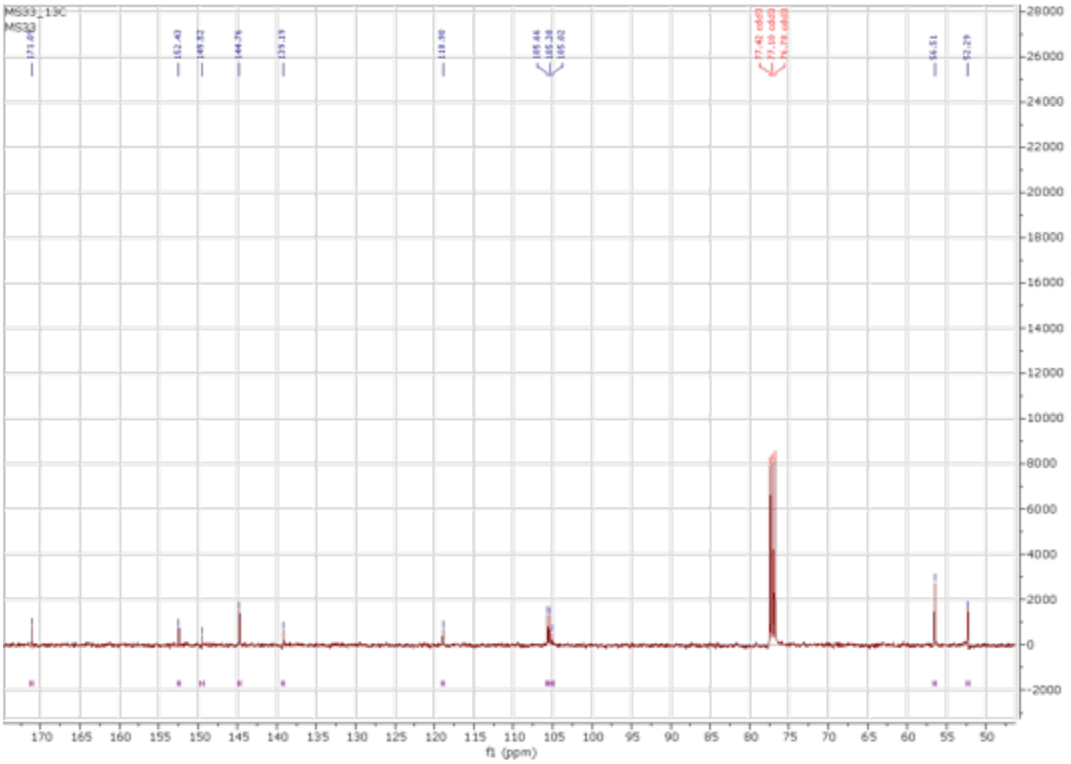

^{13}C NMR (101 MHz, Chloroform-d) δ 171.09, 152.43, 149.52, 144.76, 139.19, 118.90, 105.66, 105.38, 105.02, 56.51, 52.29.

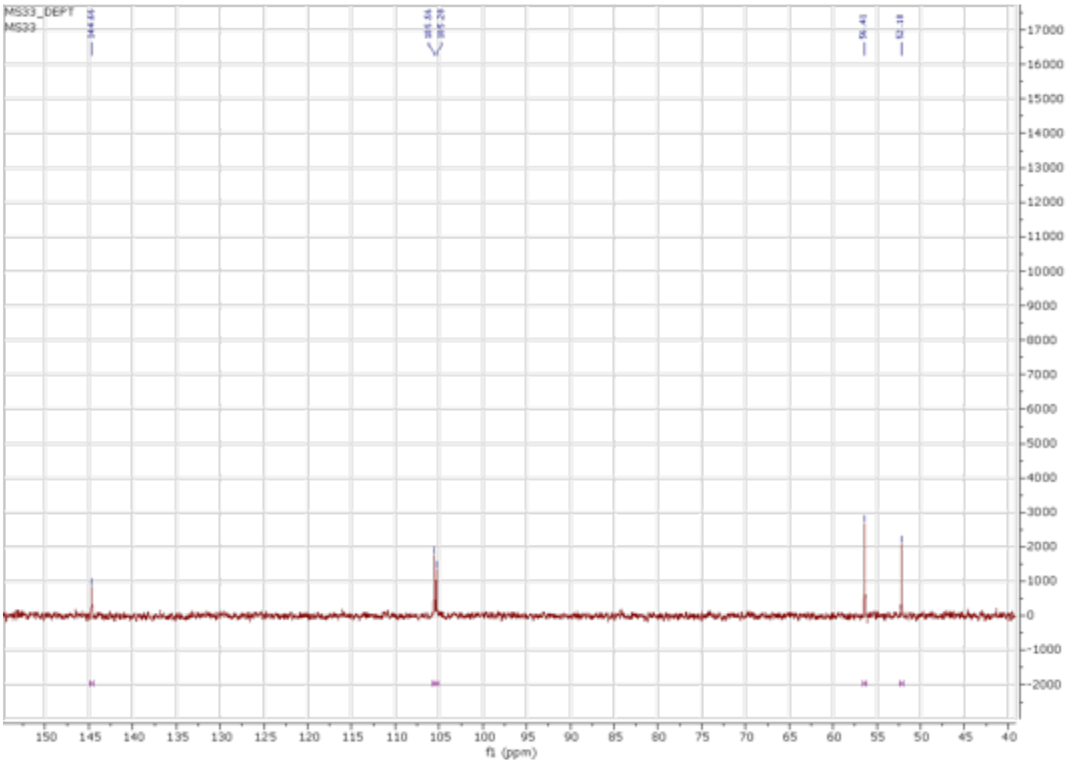

^{13}C NMR (101 MHz, Chloroform-d) δ 144.66, 105.56, 105.28, 56.41, 52.18.

methyl 4,7-dimethoxybenzofuran-5-carboxylate **(6)**

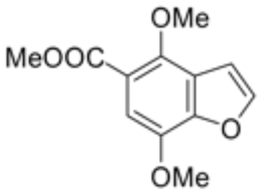

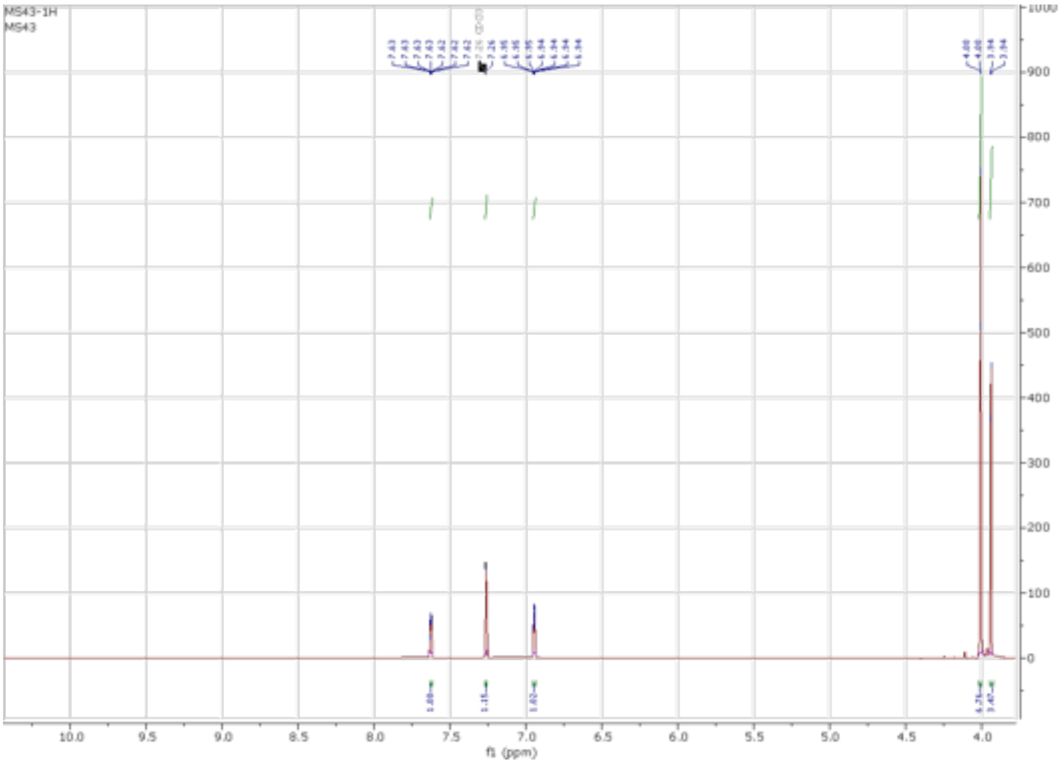

^{1}H NMR (300 MHz, Chloroform-*d*) δ 7.63 – 7.61 (m, 1H), 7.26 (s, 1H), 6.95 – 6.94 (m, 1H), 4.00 (d, *J* = 0.8 Hz, 6H), 3.94 (d, *J* = 0.8 Hz, 3H).

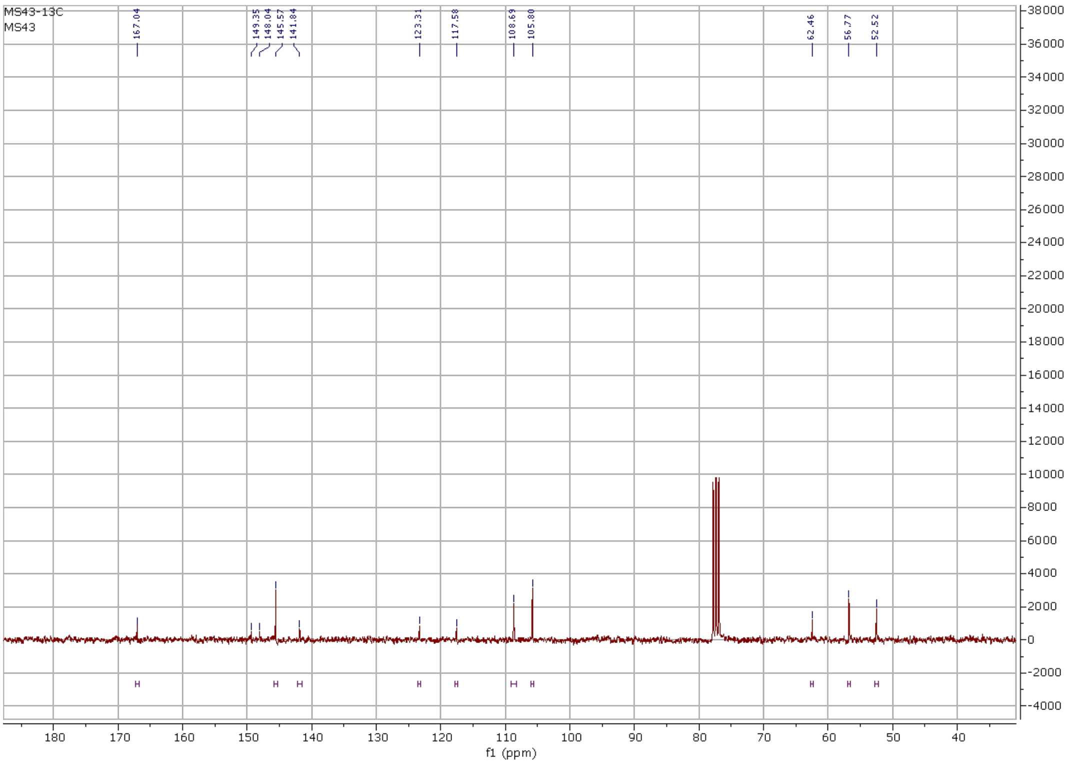

^{13}C NMR (75 MHz, Chloroform-d) δ 167.04, 149.35, 148.04, 145.57, 141.84, 123.31, 117.58, 108.69, 105.80, 62.46, 56.77, 52.52.

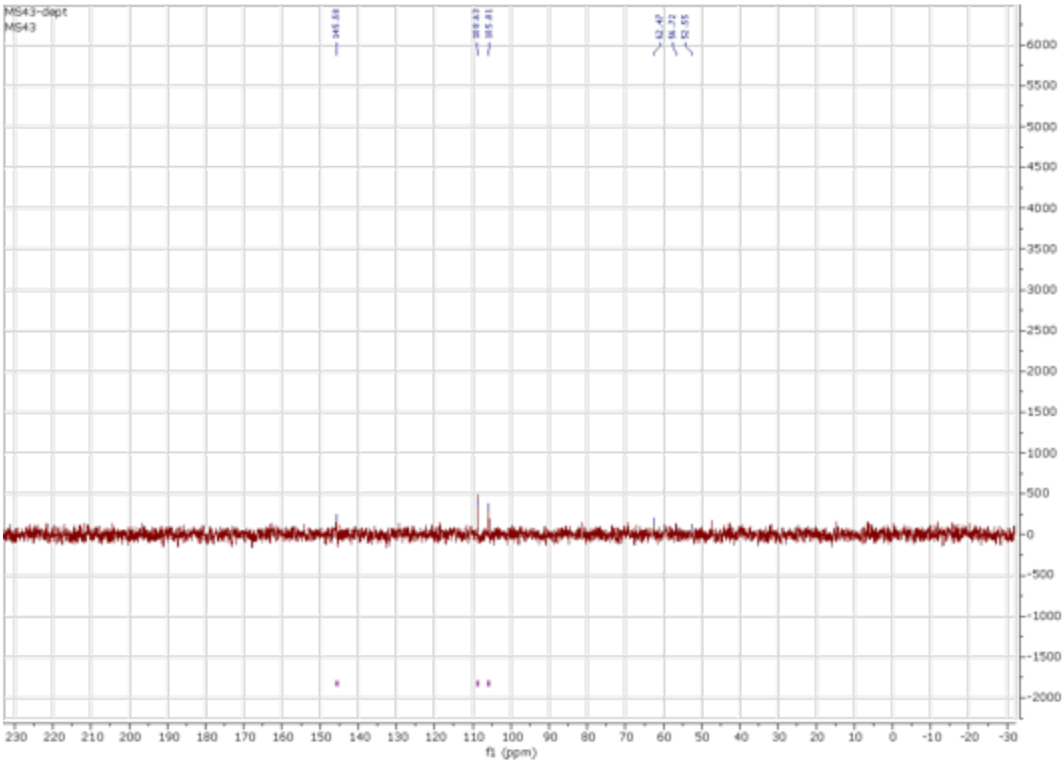

^{13}C NMR (75 MHz, Chloroform-d) δ 145.58, 108.63, 105.81, 62.47, 56.72, 52.55.

4,7-dimethoxybenzofuran-5-carbaldehyde **(7)**

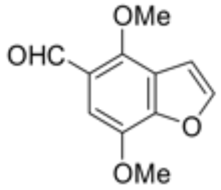

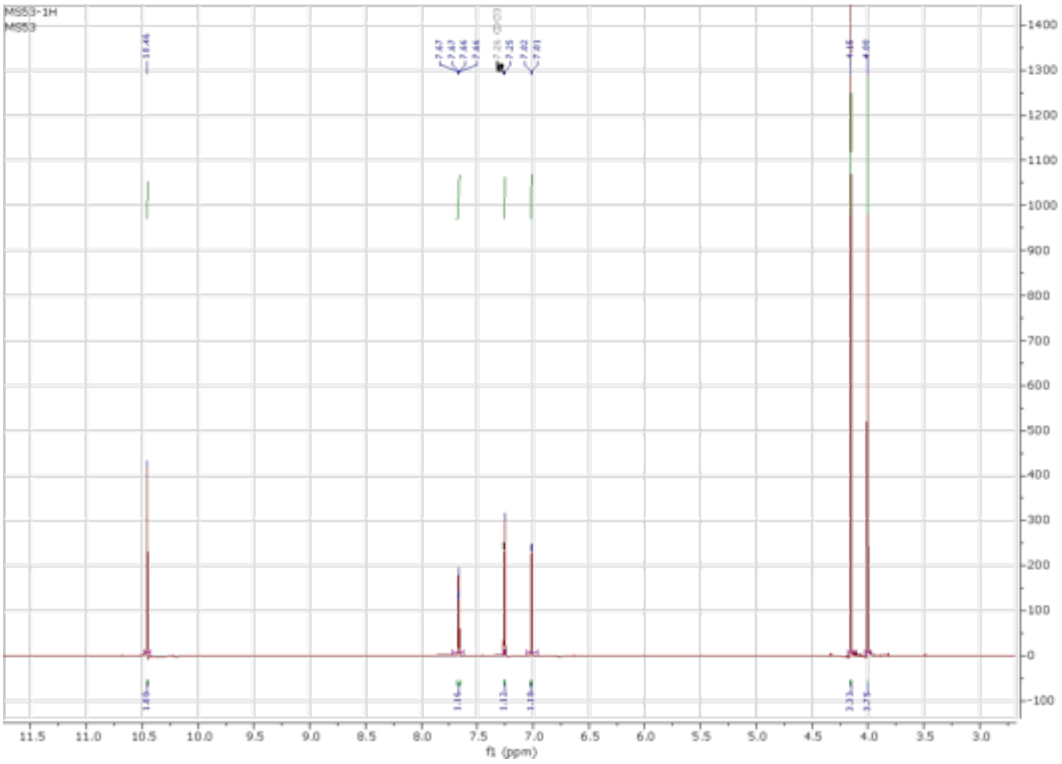

^{1}H NMR (400 MHz, Chloroform-*d*) δ 10.46 (s, 1H), 7.67 (dd, *J* = 2.3, 0.4 Hz, 1H), 7.25 (s, 1H), 7.02 (d, *J* = 2.3 Hz, 1H), 4.15 (s, 3H), 4.00 (s, 3H).

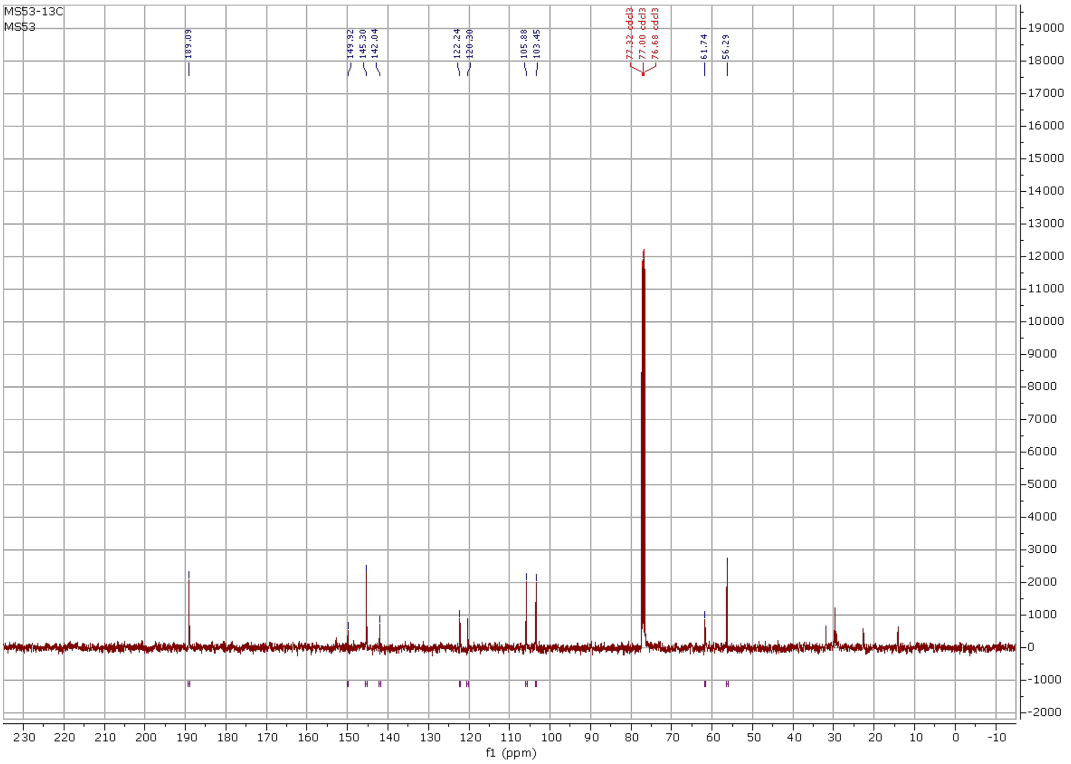

^{13}C NMR (101 MHz, Chloroform-d) δ 189.09, 149.92, 145.30, 142.04, 122.24, 120.30, 105.88, 103.45, 61.74, 56.29.

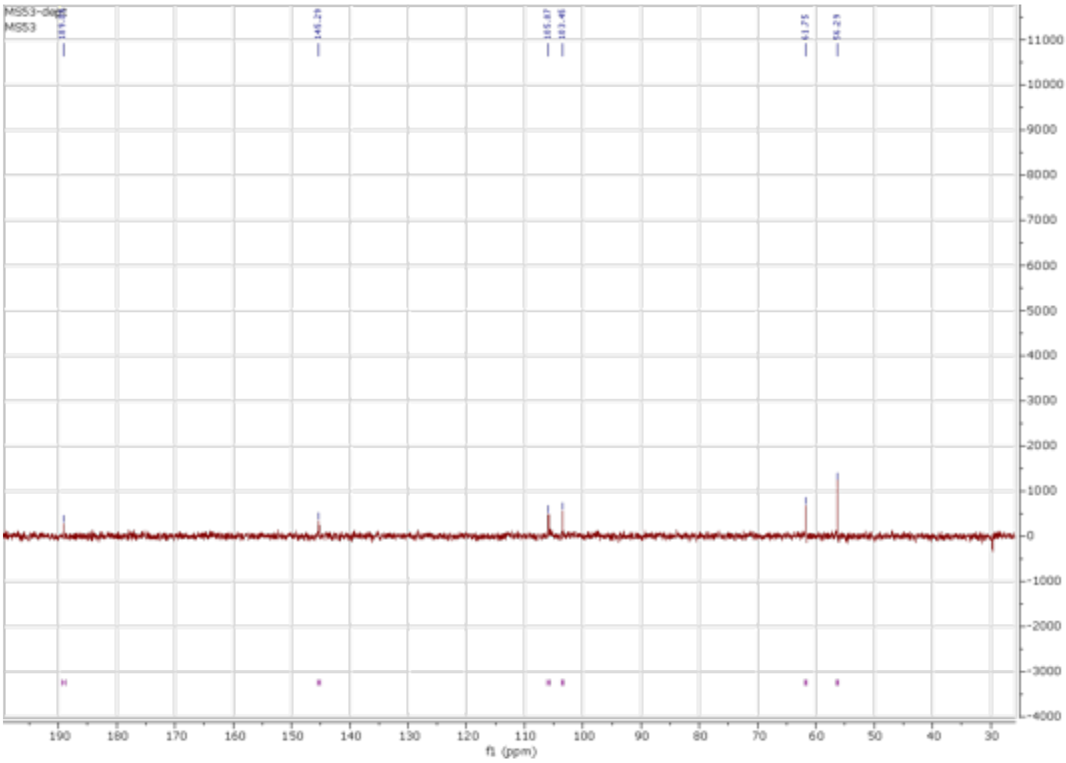

^{13}C NMR (101 MHz, Chloroform-d) δ 189.09, 145.29, 105.87, 103.45, 61.75, 56.29.

2,5-dimethoxyphenol **(9)**

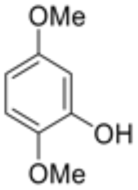

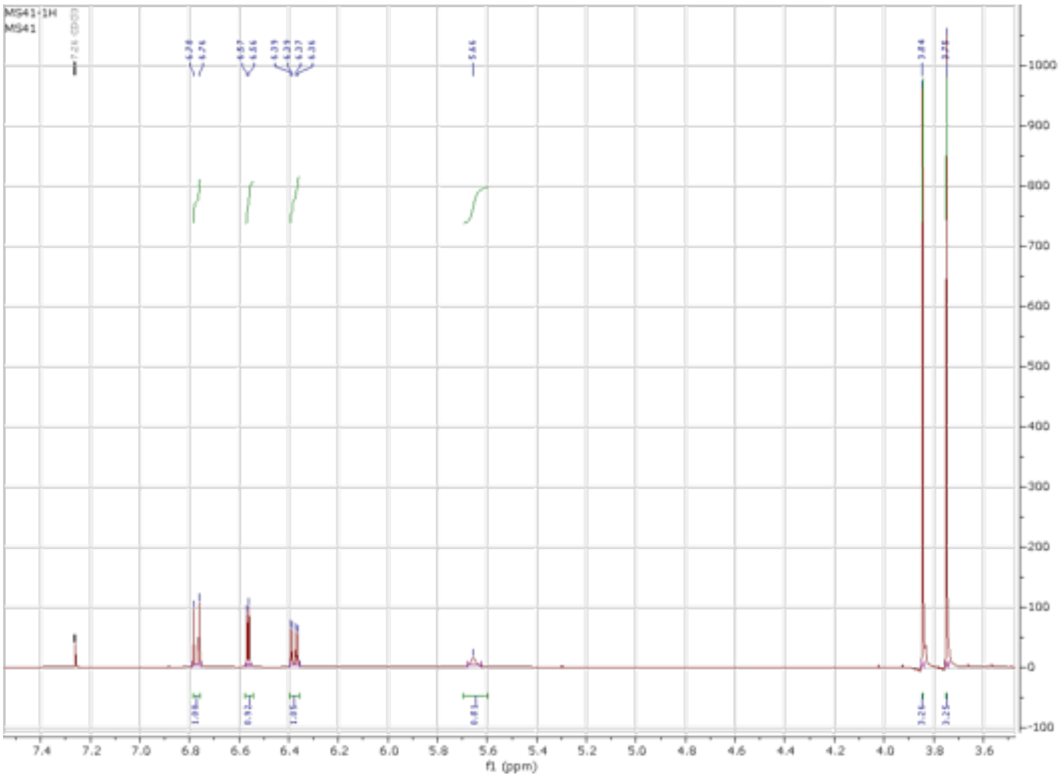

^{1}H NMR (400 MHz, Chloroform-d) δ 6.77 (d, *J* = 8.8 Hz, 1H), 6.56 (d, *J* = 2.9 Hz, 1H), 6.38 (dd, *J* = 8.8, 2.9 Hz, 1H), 5.66 (s, 1H), 3.84 (s, 3H), 3.75 (s, 3H).

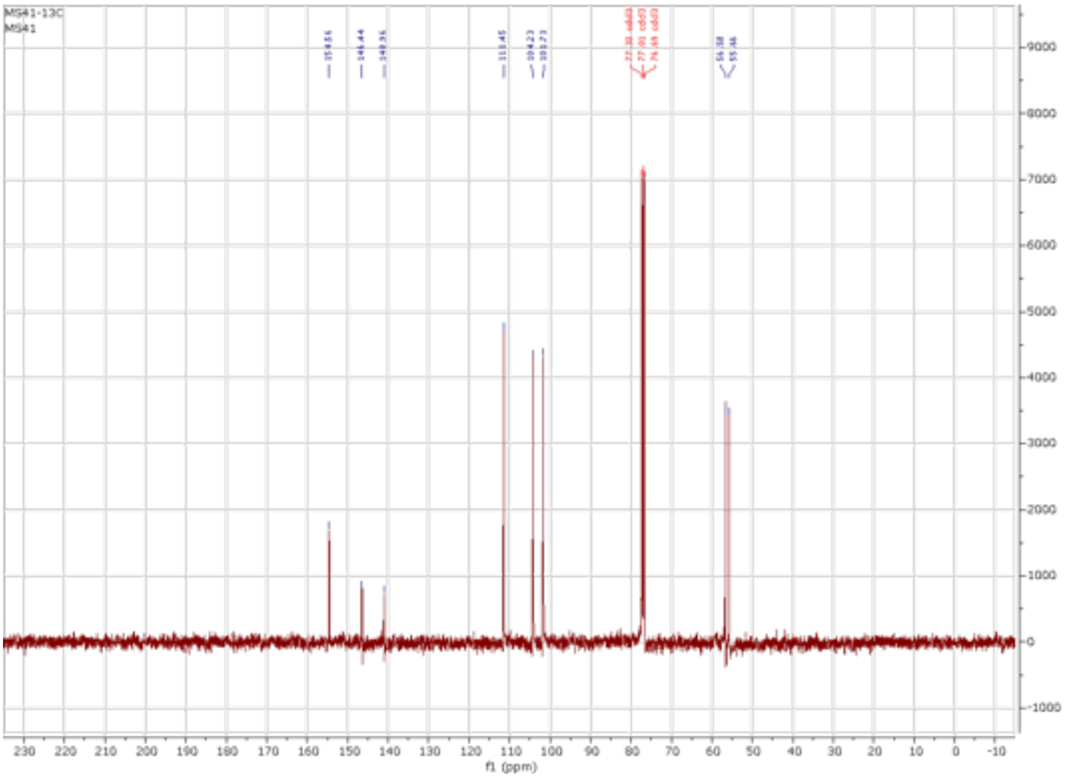

^{13}C NMR (101 MHz, Chloroform-d) δ 154.56, 146.44, 140.96, 111.45, 104.23, 101.73, 56.58, 55.66.

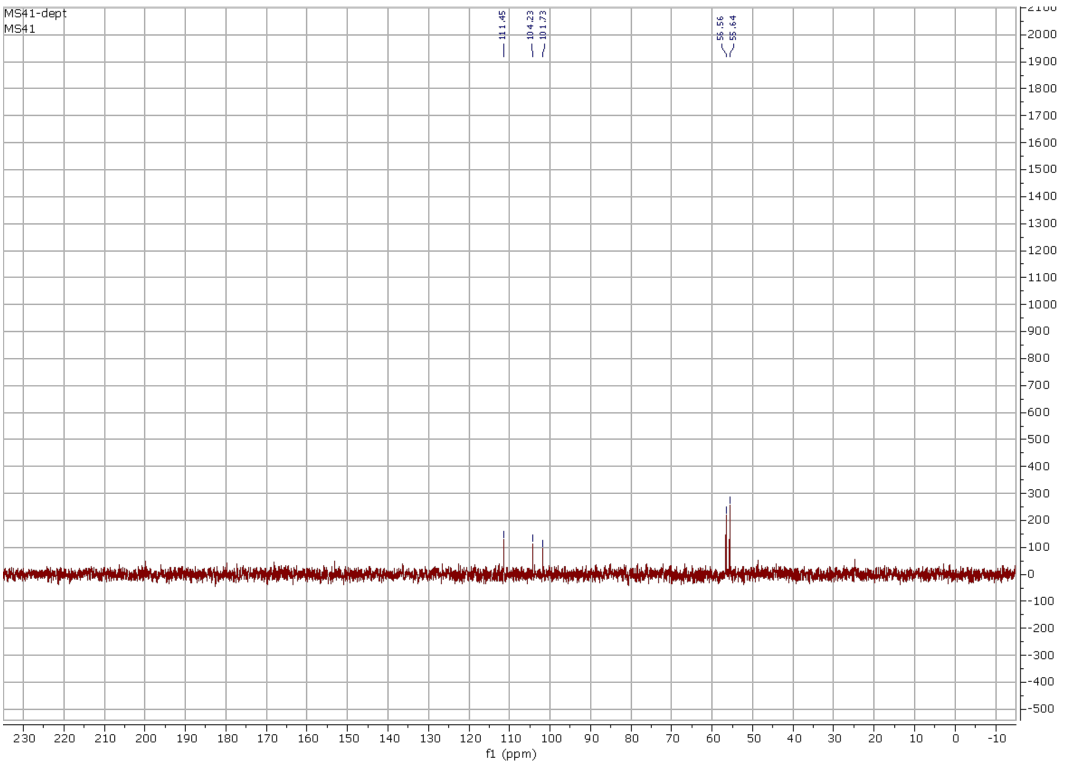

^{13}C NMR (101 MHz, Chloroform-d) δ 111.45, 104.23, 101.73, 56.56, 55.64.

4-bromo-2,5-dimethoxyphenol **(10)**

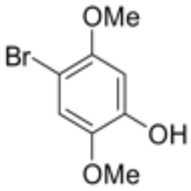

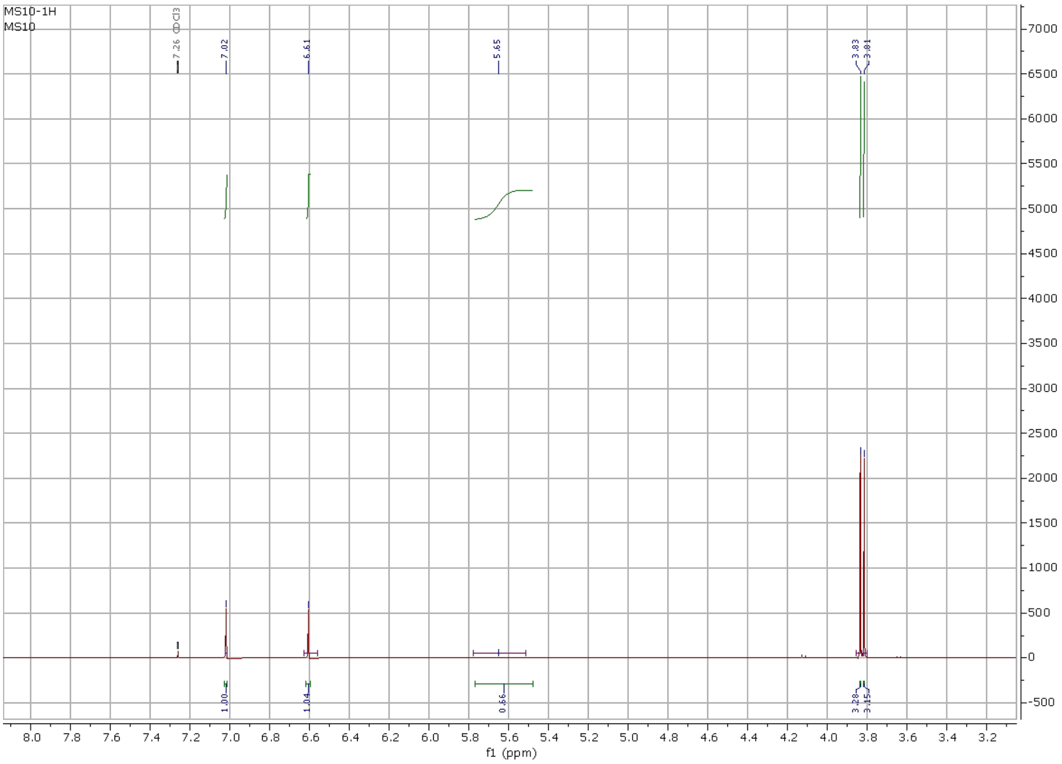

^{1}H NMR (400 MHz, Chloroform-d) δ 7.02 (s, 1H), 6.61 (s, 1H), 5.65 (s, 1H), 3.83 (s, 3H), 3.81 (s, 3H).

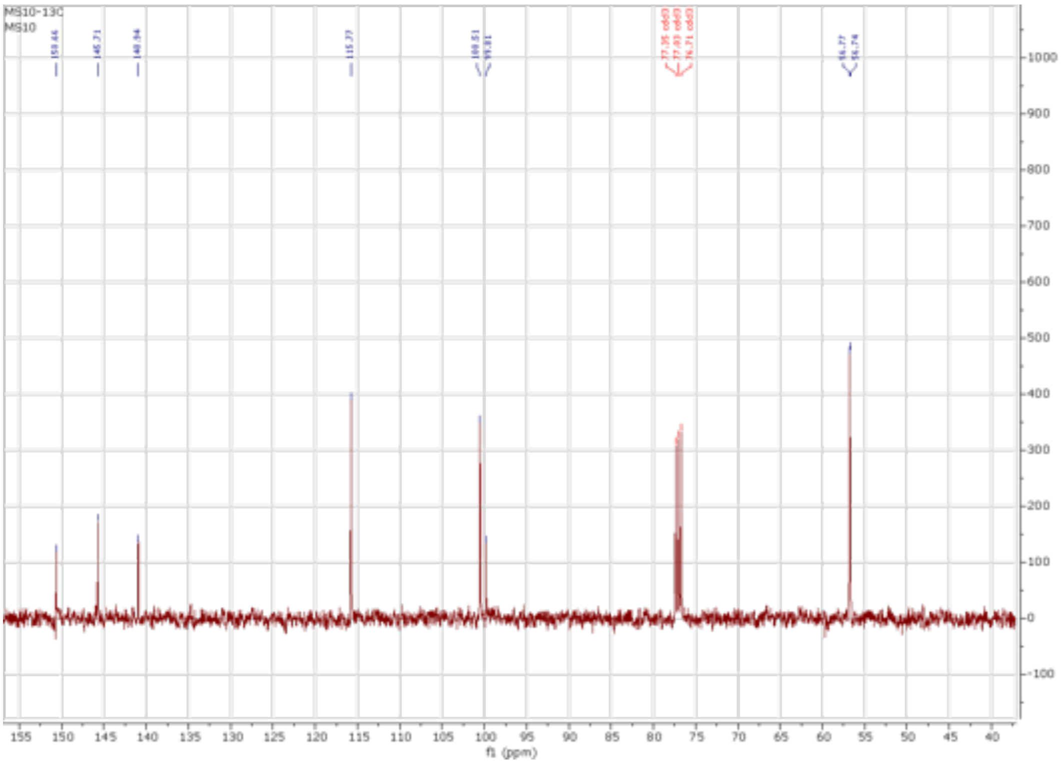

^{13}C NMR (101 MHz, Chloroform-d) δ 150.66, 145.71, 140.94, 115.77, 100.51, 99.81, 56.77, 56.74.

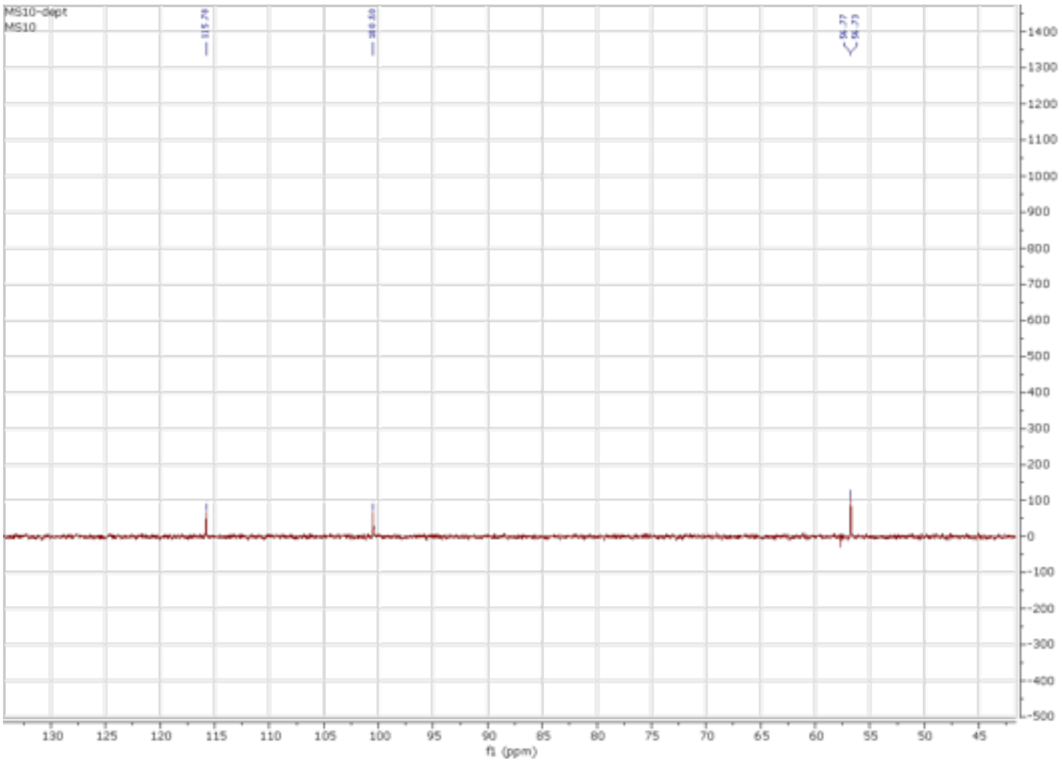

^{13}C NMR (101 MHz, Chloroform-d) δ 115.78, 100.50, 56.77, 56.73.

1-bromo-4-(2,2-dimethoxyethoxy)-2,5-dimethoxybenzene **(11)**

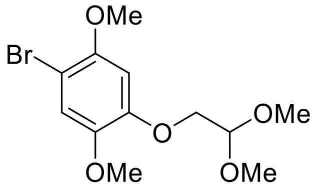

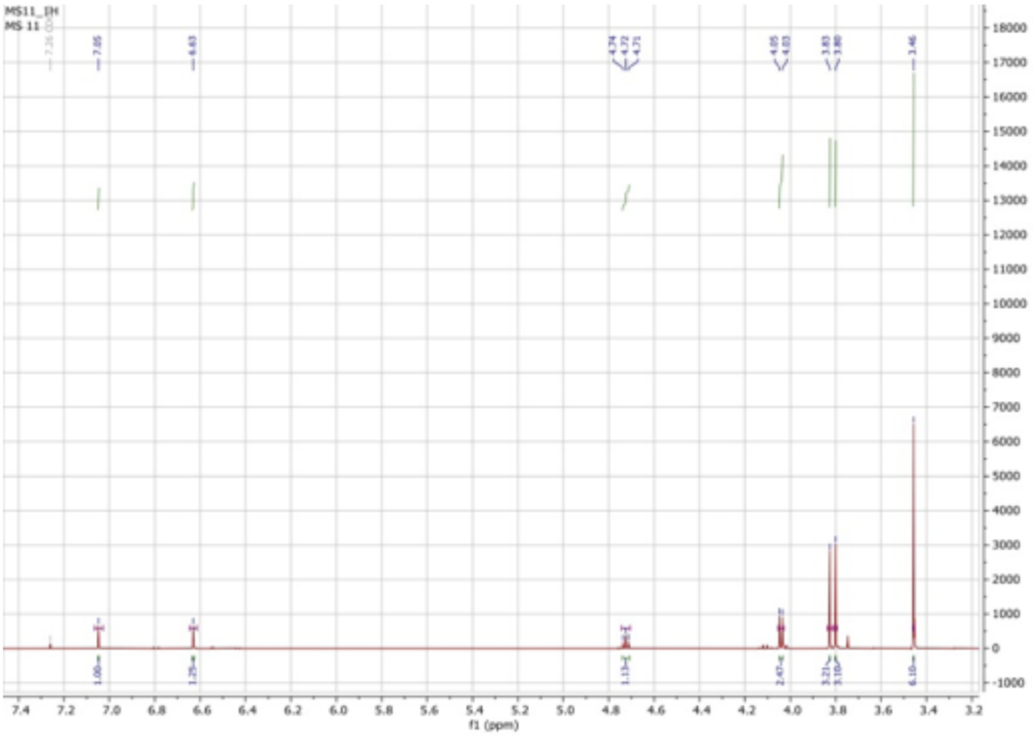

^{1}H NMR (400 MHz, Chloroform-d) δ 7.05 (s, 1H), 6.63 (s, 1H), 4.72 (t, *J* = 5.2 Hz, 1H), 4.04 (d, *J* = 5.2 Hz, 0H), 3.83 (s, 3H), 3.80 (s, 3H), 3.46 (s, 6H).

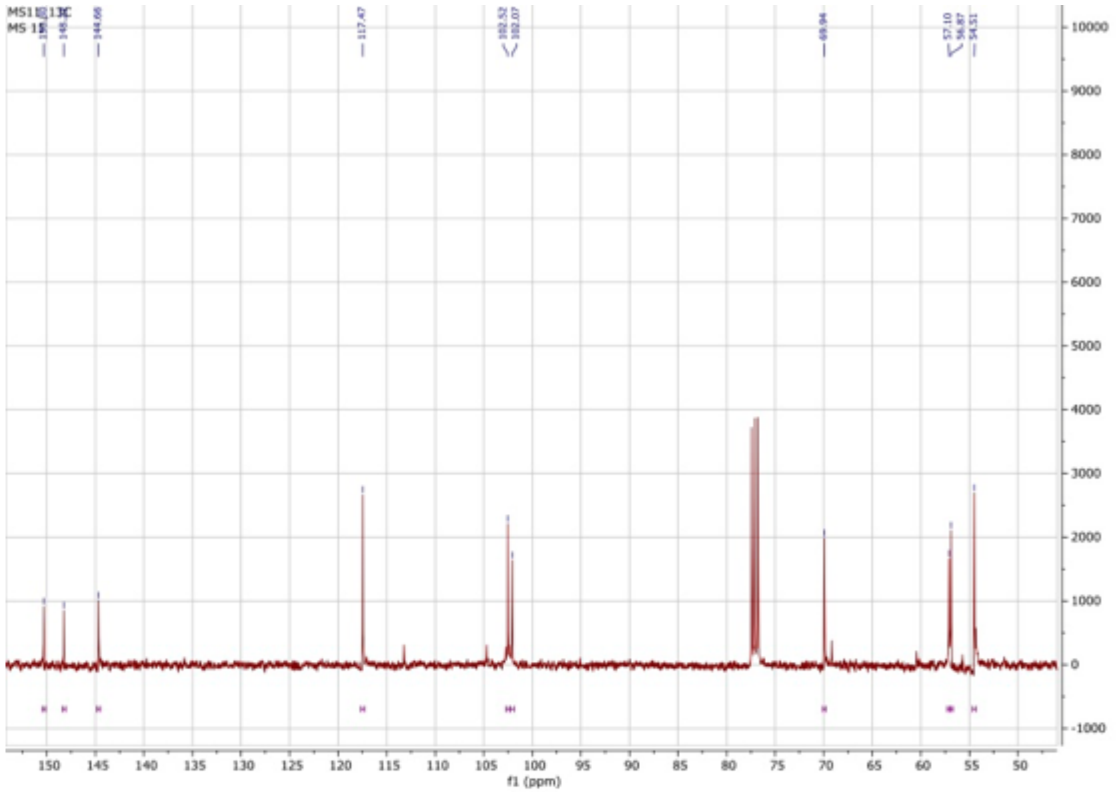

^{13}C NMR (101 MHz, Chloroform-d) δ 150.30, 148.22, 144.66, 117.47, 102.52, 102.07, 69.94, 57.10, 56.87, 54.51.

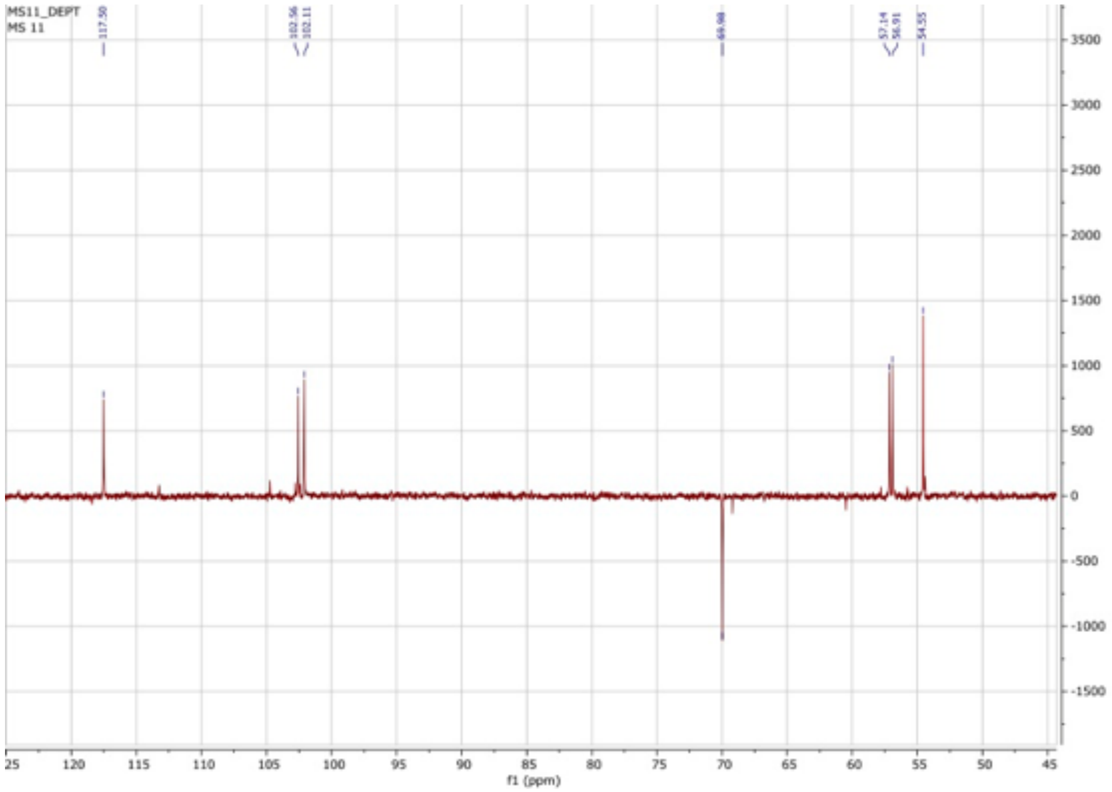

^{13}C NMR (101 MHz, Chloroform-d) δ 117.50, 102.56, 102.11, 69.98, 57.14, 56.91, 54.55.

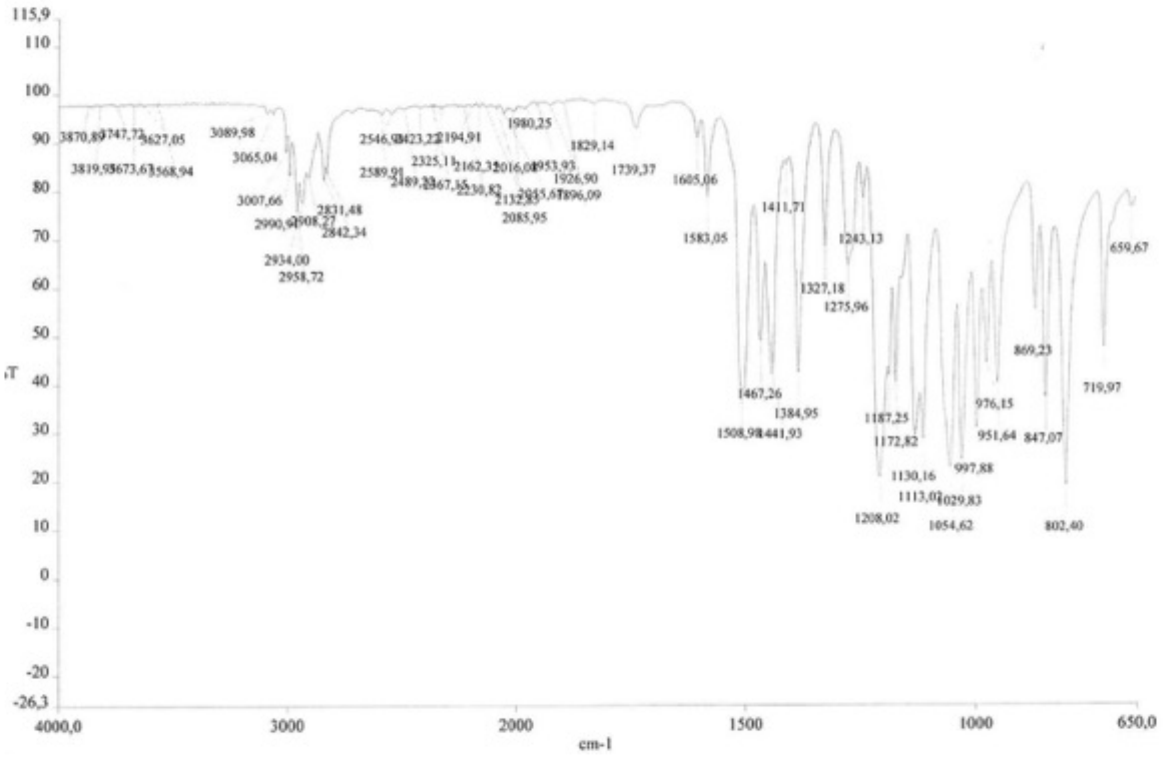

5-bromo-4,7-dimethoxybenzofuran (12)

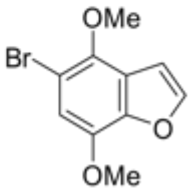

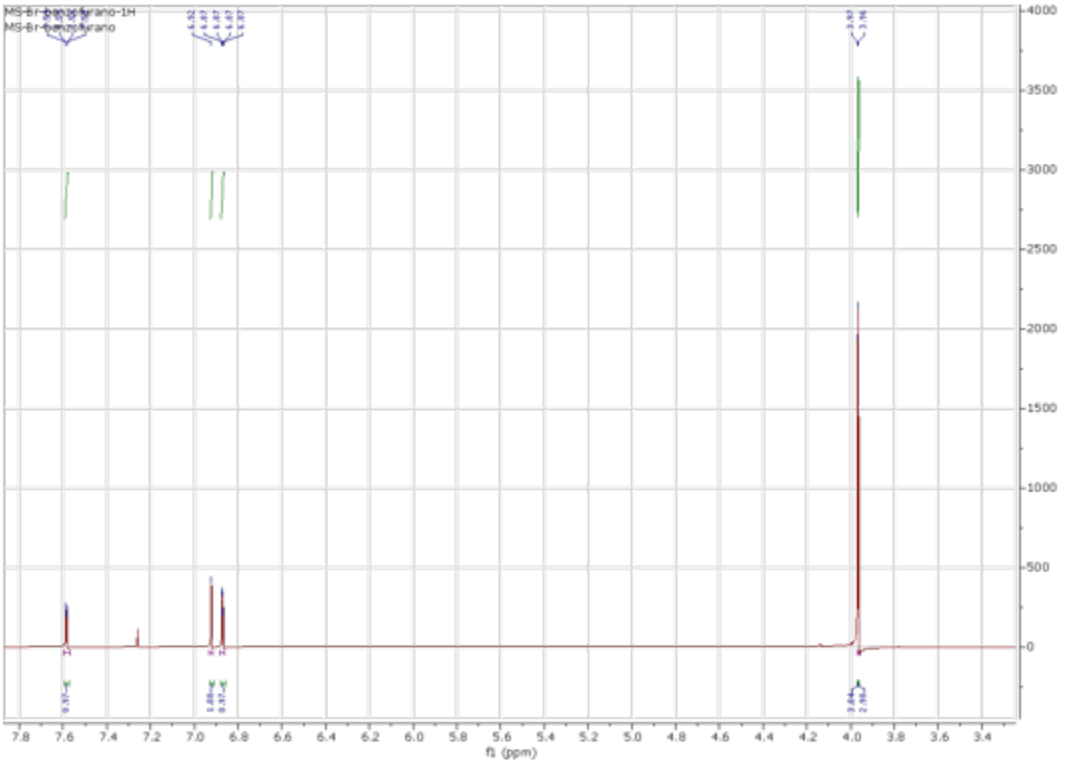

^{1}H NMR (400 MHz, Chloroform-*d*) δ 7.59 (dd, *J* = 2.2, 0.5 Hz, 1H), 6.92 (s, 1H), 6.87 (dd, *J* = 2.1, 0.4 Hz, 1H), 3.97 (d, *J* = 1.7 Hz, 6H).

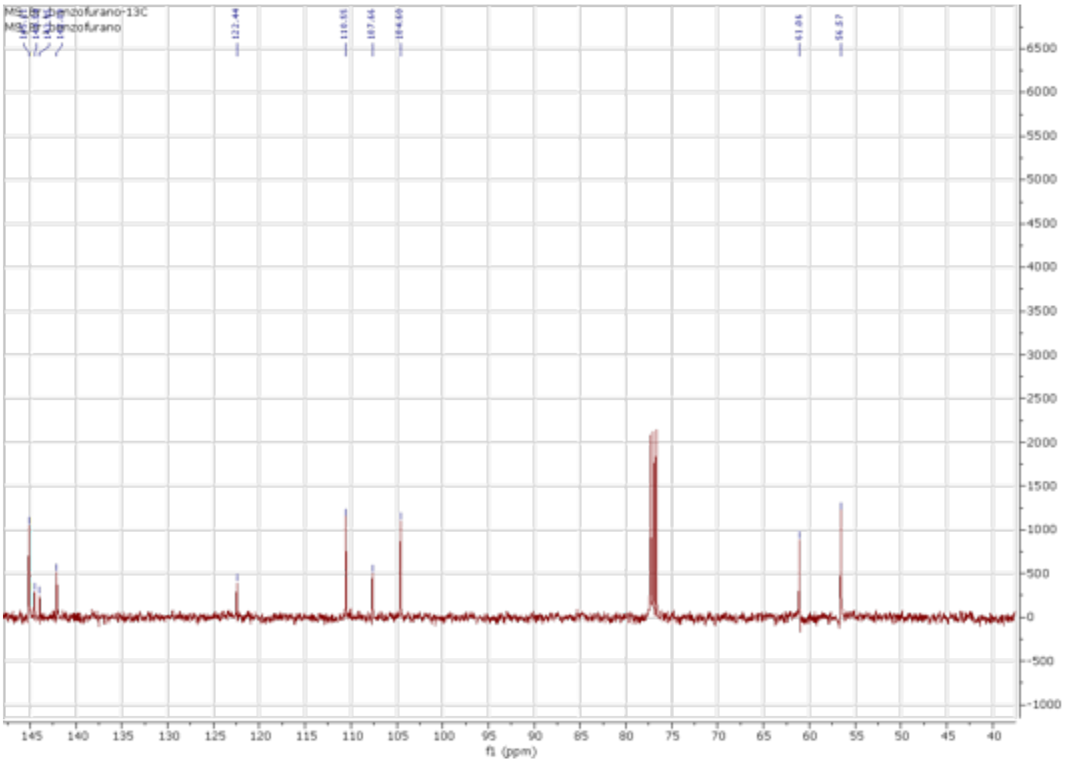

^{13}C NMR (101 MHz, Chloroform-d) δ 145.11, 144.54, 143.95, 142.09, 122.44, 110.55, 107.66, 104.60, 61.06, 56.57.

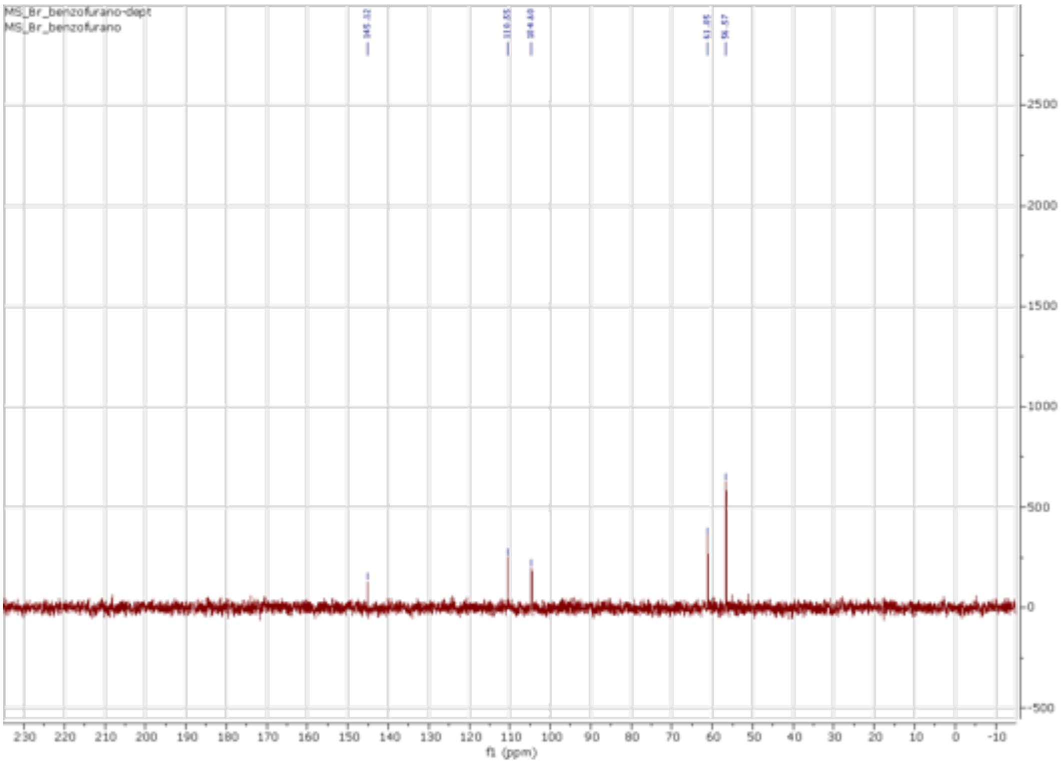

^{13}C NMR (101 MHz, Chloroform-d) δ 145.12, 110.55, 104.60, 61.05, 56.57.

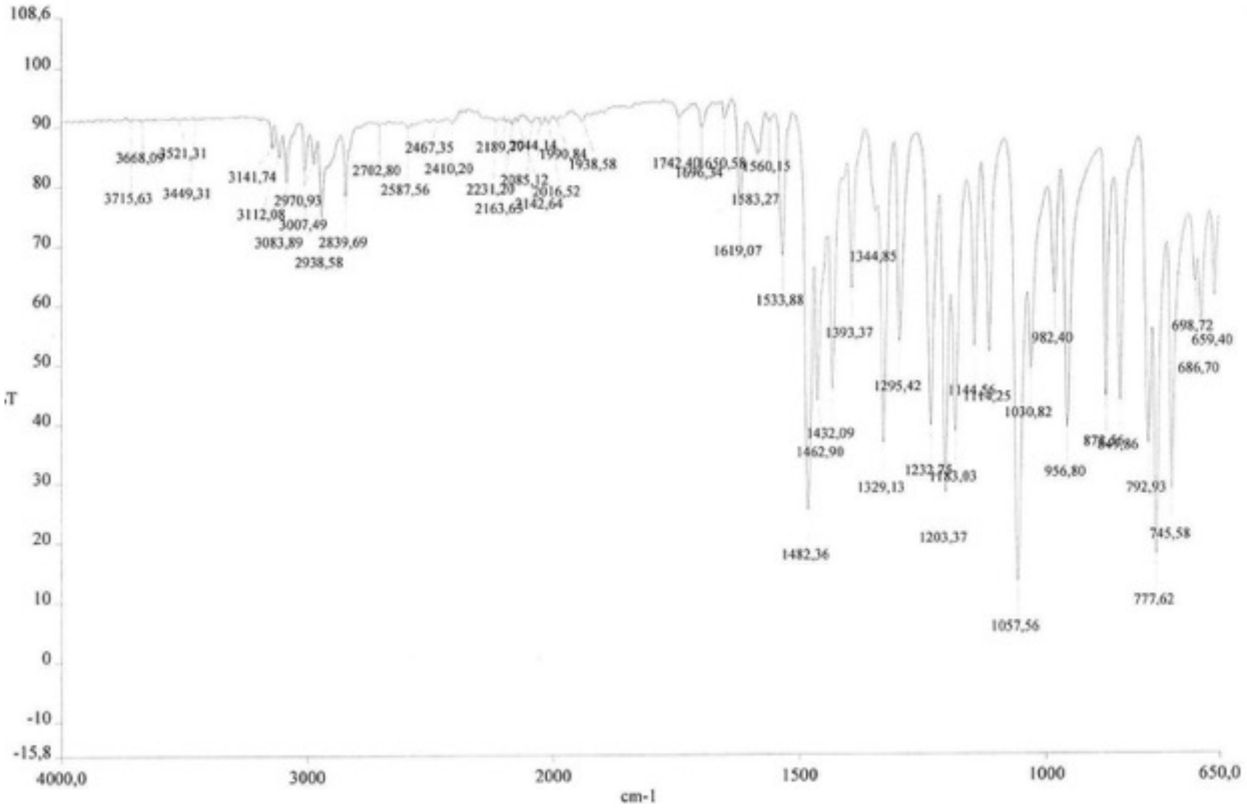

1-phenylprop-2-en-1-one **(13)**

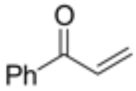

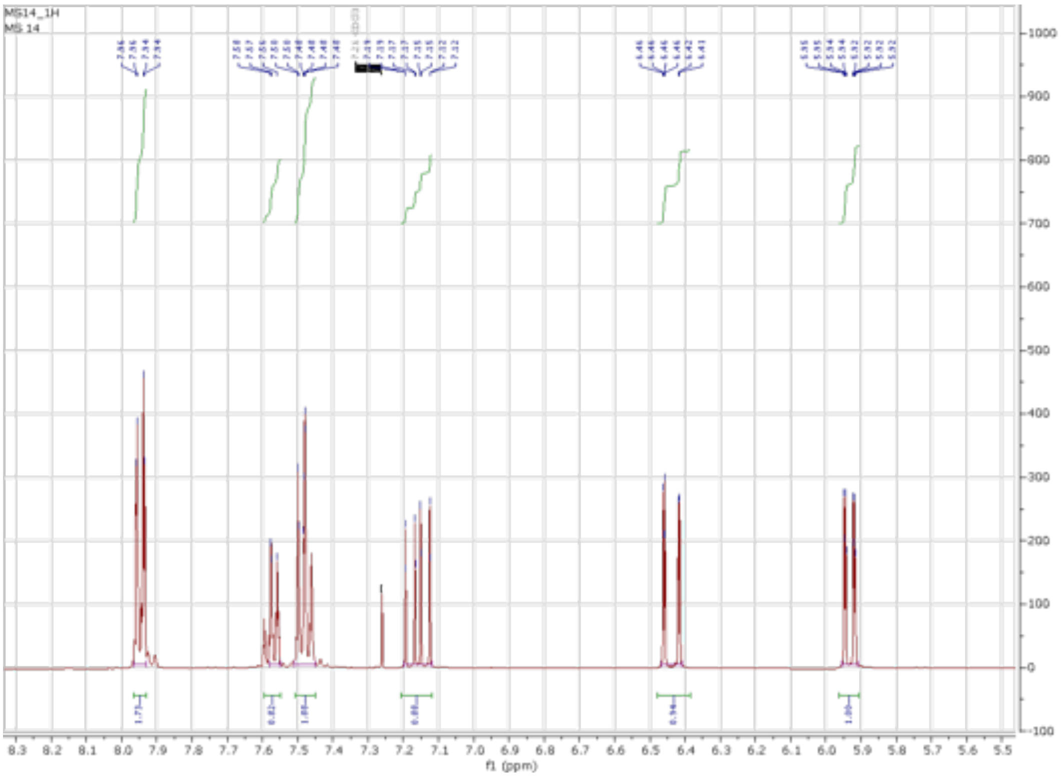

^{1}H NMR (400 MHz, Chloroform-d) δ 7.97 – 7.93 (m, 2H), 7.58 – 7.55 (m, 1H), 7.51 – 7.45 (m, 2H), 7.16 (ddd, *J* = 17.2, 10.6, 0.6 Hz, 1H), 6.44 (dd, *J* = 17.2, 1.7 Hz, 1H), 5.95 – 5.91 (m, 1H).

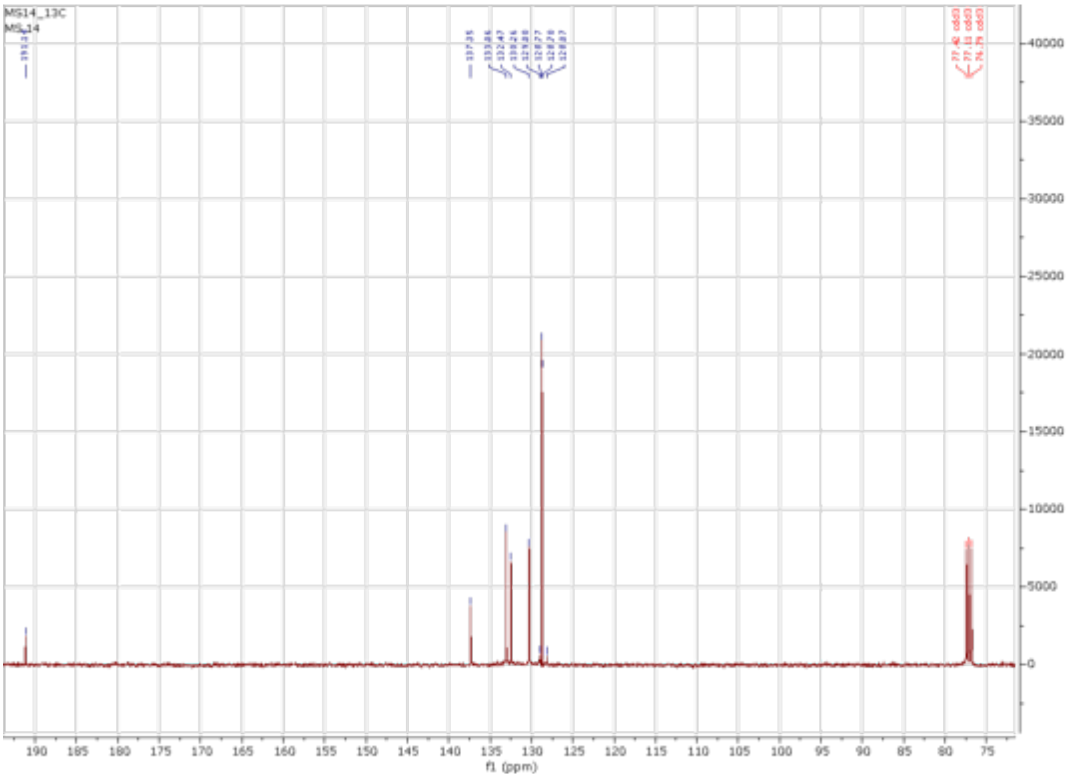

^{13}C NMR (101 MHz, Chloroform-d) δ 191.14, 137.35, 133.06, 132.47, 130.26, 129.00, 128.77, 128.70, 128.07.

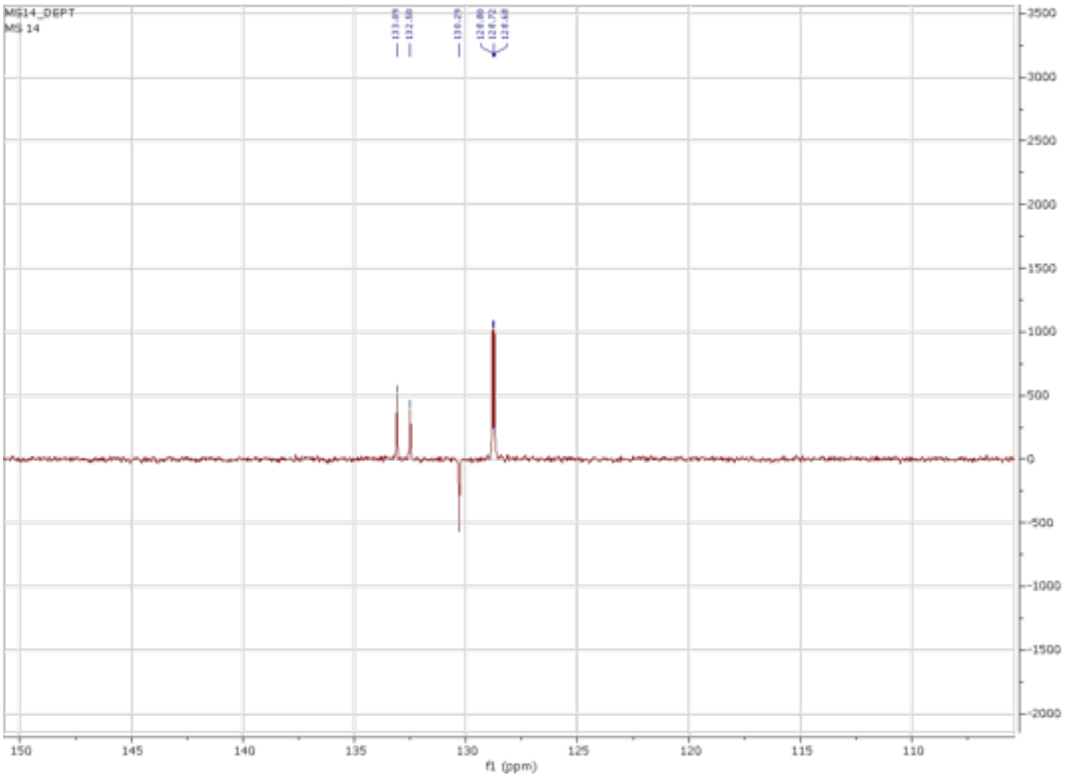

^{13}C NMR (101 MHz, Chloroform-d) δ 133.09, 132.50, 130.29, 128.80, 128.72, 128.68.

2-(2,5-dimethoxyphenoxy)tetrahydro-2*H*-pyran **(14)**

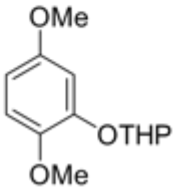

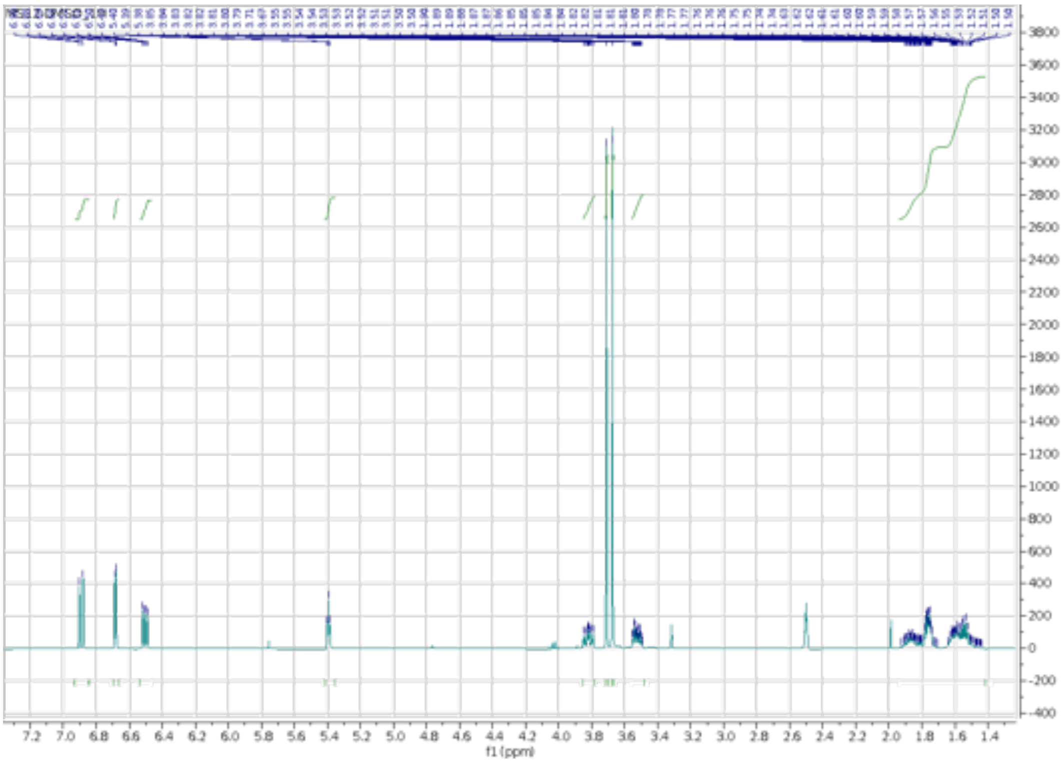

^{1}H NMR (400 MHz, dmso) δ 6.89 (d, *J* = 8.8 Hz, 1H), 6.68 (d, *J* = 2.9 Hz, 1H), 6.50 (dd, *J* = 8.9, 2.9 Hz, 1H), 5.39 (t, *J* = 3.3 Hz, 1H), 3.82 (ddd, *J* = 11.1, 8.9, 3.8 Hz, 1H), 3.71 (s, 3H), 3.67 (s, 3H), 3.53 (dtd, *J* = 11.5, 4.3, 1.2 Hz, 1H), 1.94 – 1.38 (m, 7H).

2-hydroxy-3,6-dimethoxybenzaldehyde **(15)**

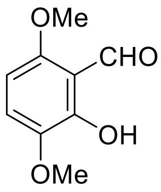

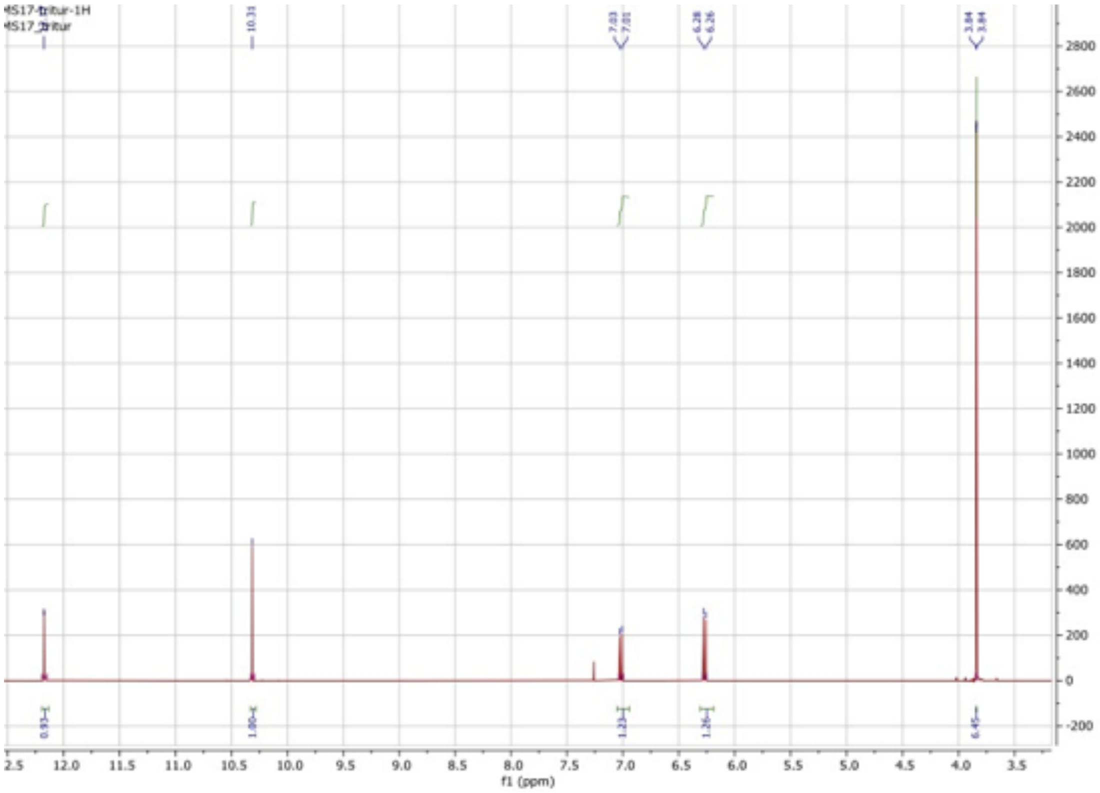

^{1}H NMR (400 MHz, Chloroform-*d*) δ 12.17 (s, 1H), 10.31 (s, 1H), 7.02 (d, *J* = 8.9 Hz, 1H), 6.27 (d, *J* = 8.9 Hz, 1H), 3.84 (d, *J* = 0.8 Hz, 6H).

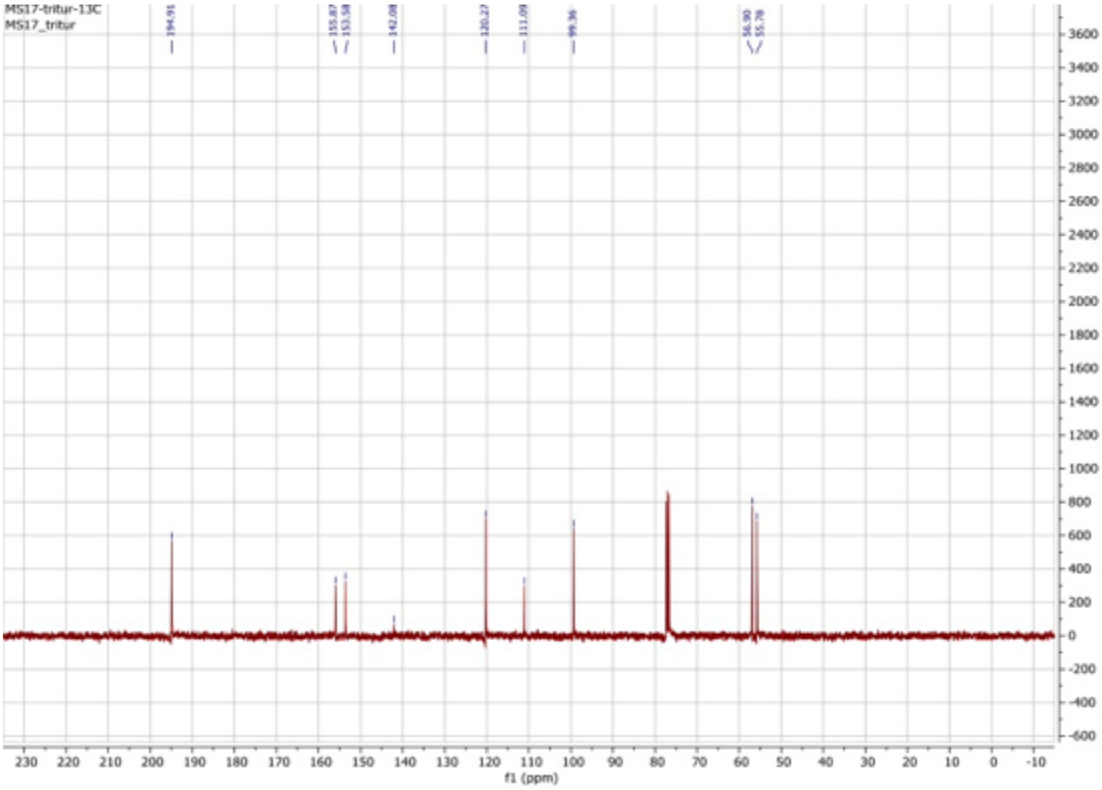

^{13}C NMR (101 MHz, Chloroform-d) δ 194.91, 155.87, 153.58, 142.08, 120.27, 111.09, 99.36, 56.90, 55.78.

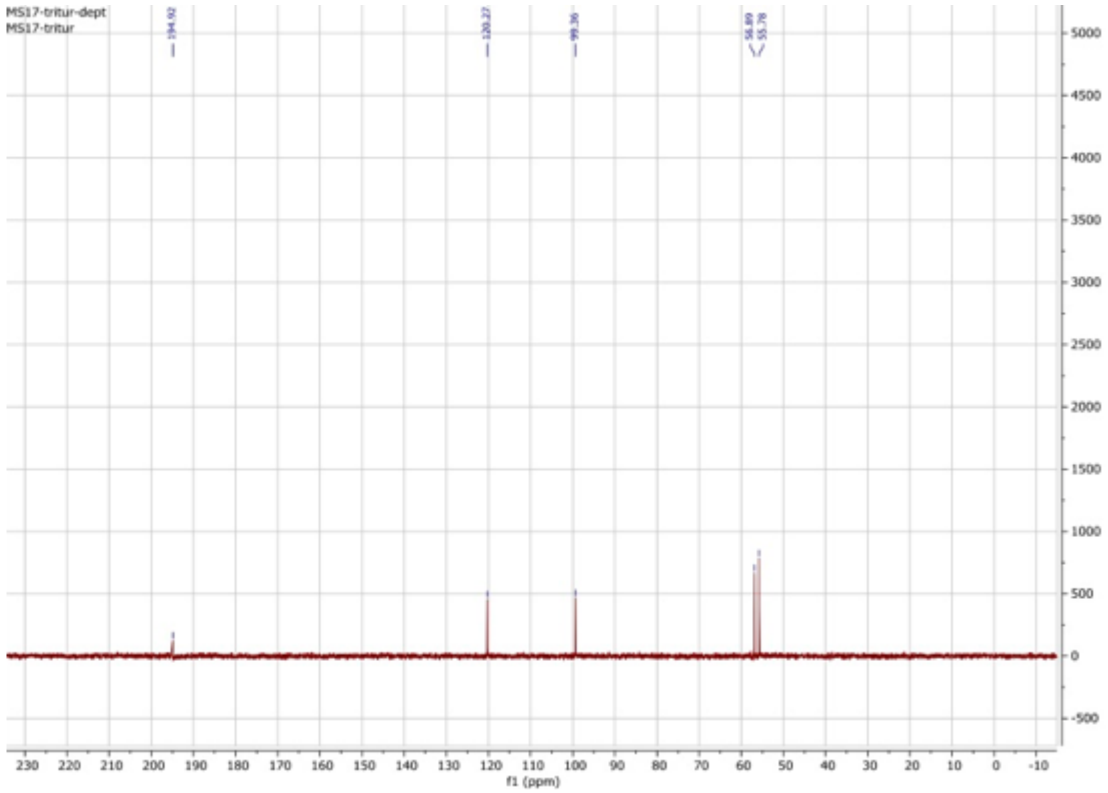

^{13}C NMR (101 MHz, Chloroform-d) δ 194.92, 120.27, 99.36, 56.89, 55.78.

3-bromo-6-hydroxy-2,5-dimethoxybenzaldehyde **(16)**

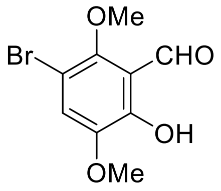

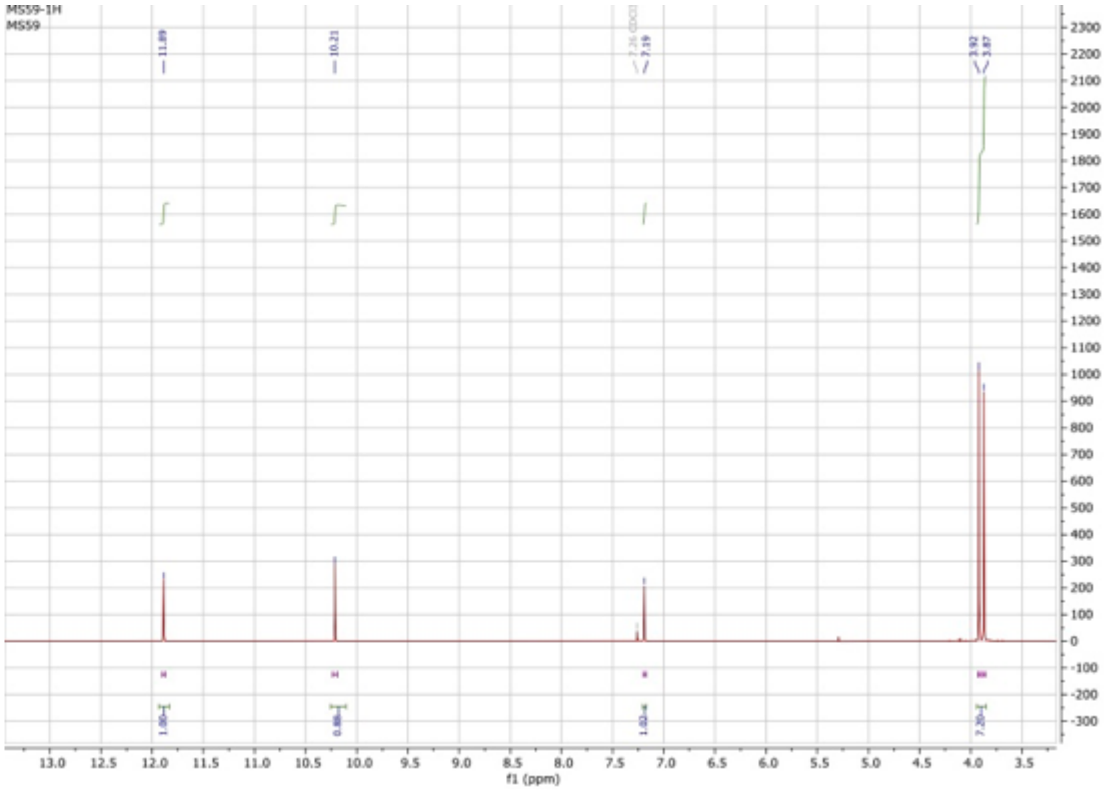

^{1}H NMR (400 MHz, Chloroform-*d*) δ 11.89 (s, 1H), 10.21 (s, 1H), 7.19 (s, 1H), 3.92 (s, 3H), 3.87 (s, 3H).

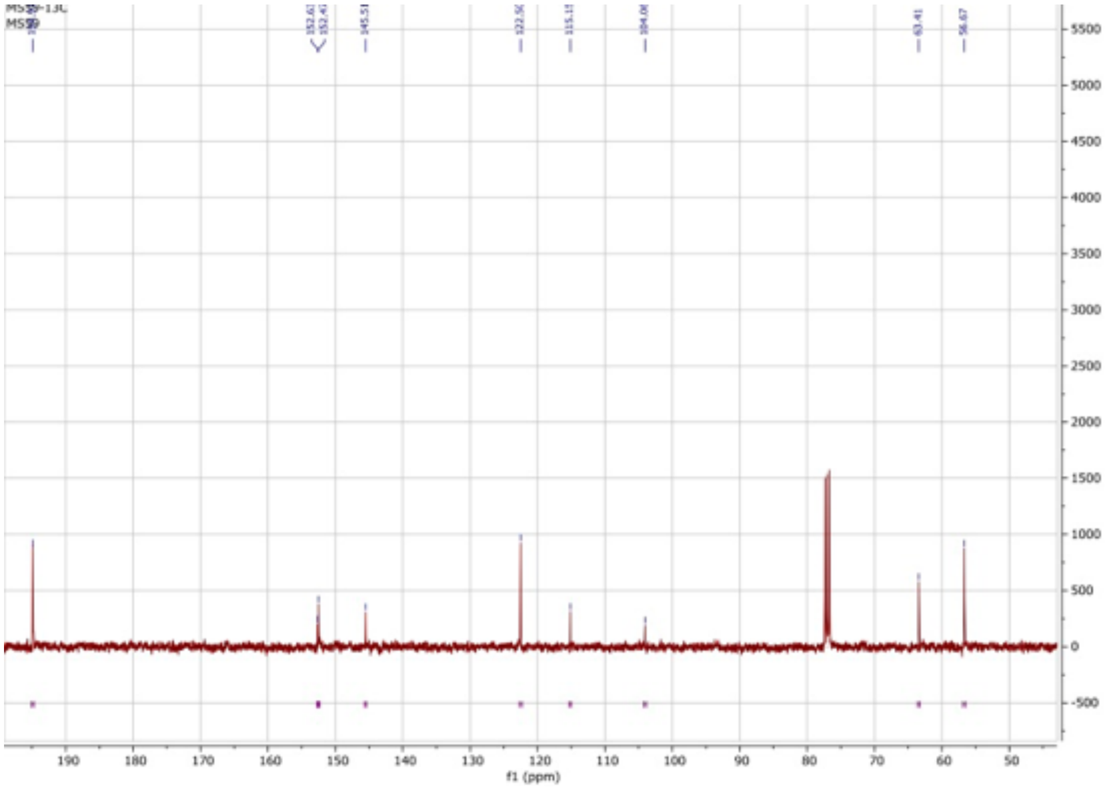

^{13}C NMR (101 MHz, Chloroform-d) δ 194.91, 152.63, 152.47, 145.51, 122.50, 115.15, 104.06, 63.41, 56.67.

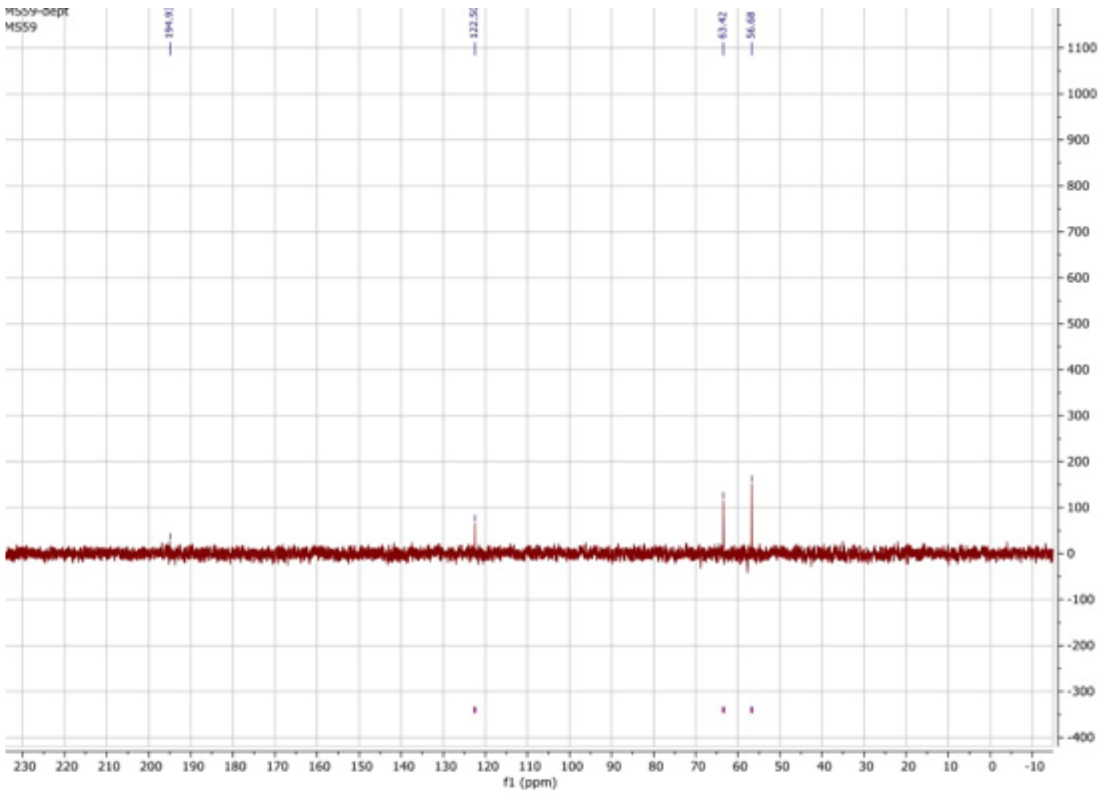

^{13}C NMR (101 MHz, Chloroform-d) δ 194.93, 122.50, 63.42, 56.68.

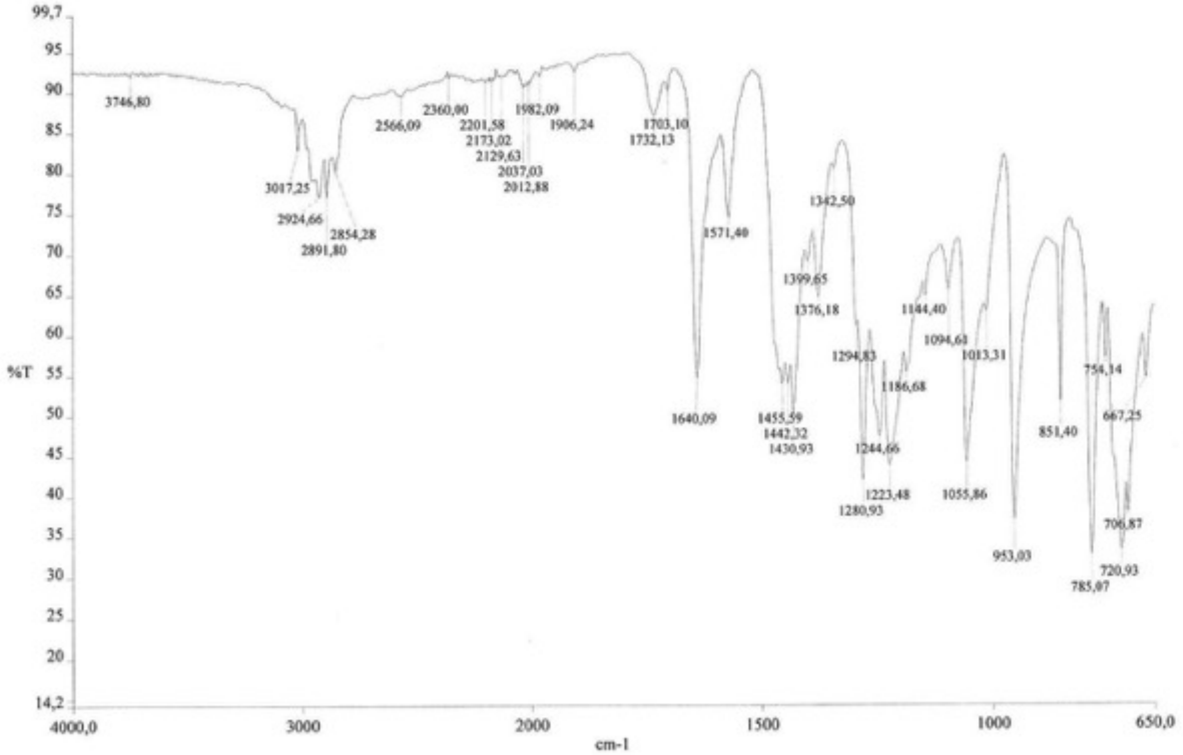

ethyl 2-(4-bromo-2-formyl-3,6-dimethoxyphenoxy)acetate **(17)**

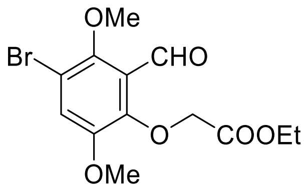

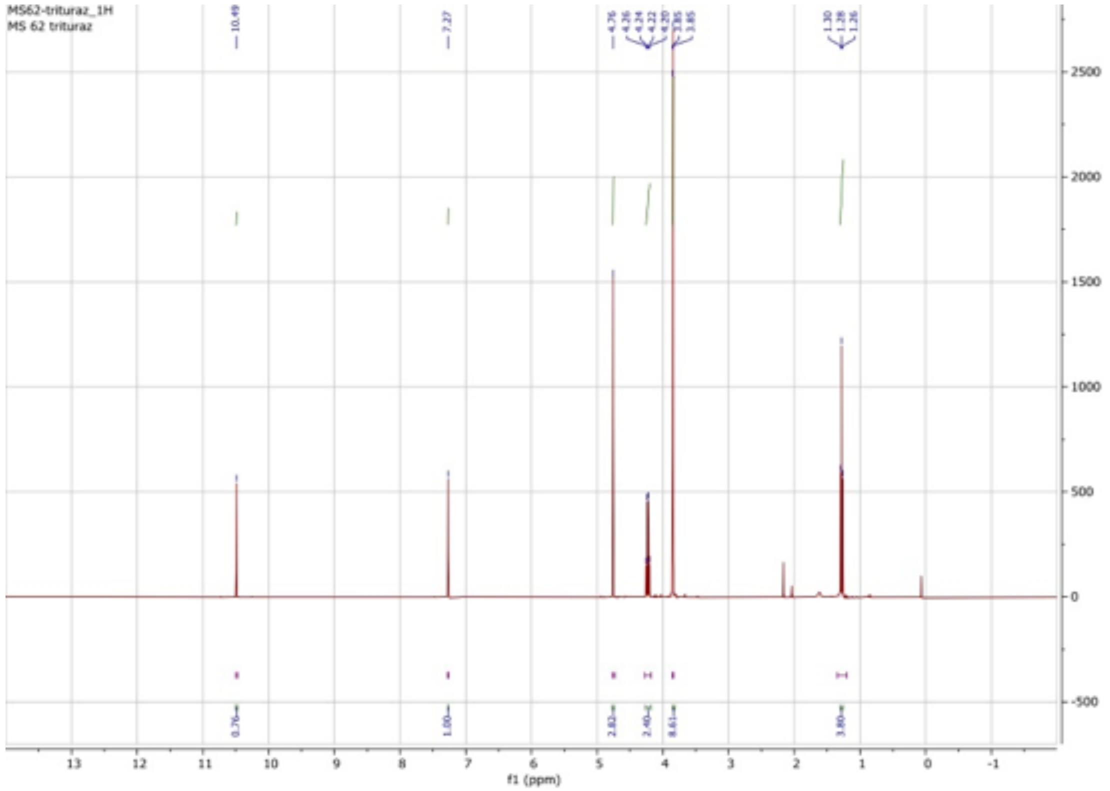

^{1}H NMR (400 MHz, Chloroform-*d*) δ 10.49 (s, 1H), 7.27 (s, 1H), 4.76 (s, 2H), 4.23 (q, *J* = 7.1 Hz, 2H), 3.85 (d, *J* = 2.1 Hz, 6H), 1.28 (t, *J* = 7.1 Hz, 3H).

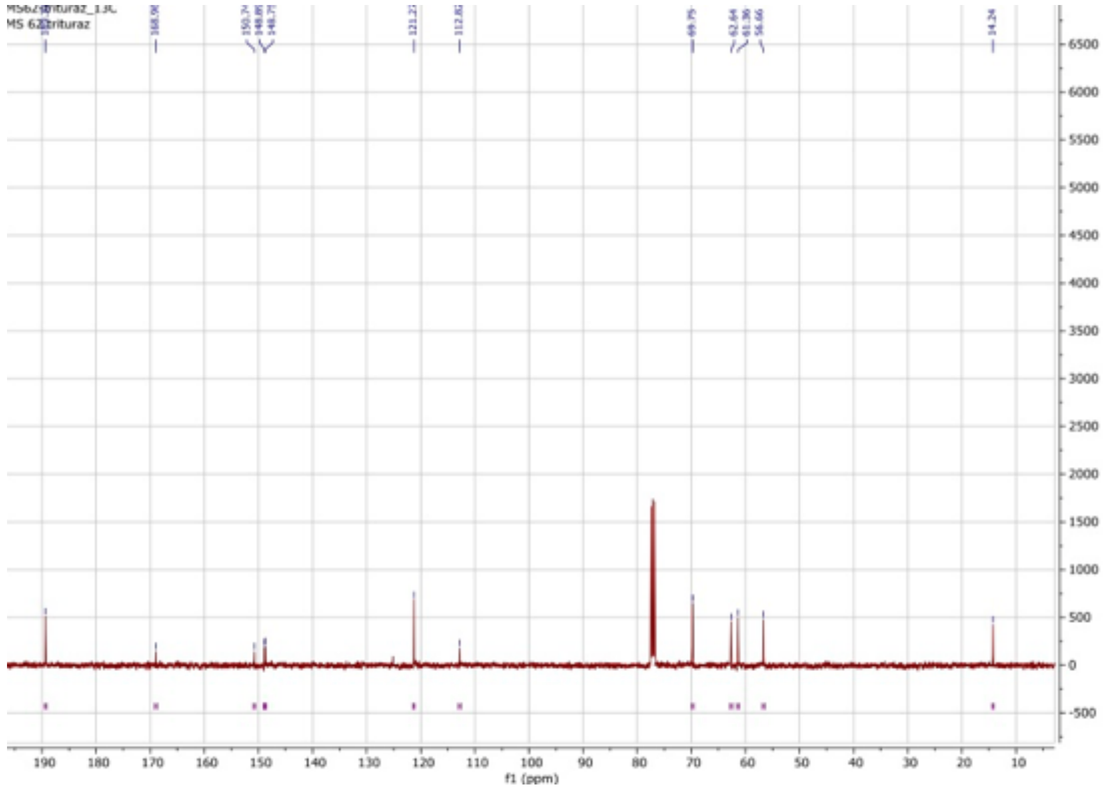

^{13}C NMR (101 MHz, Chloroform-d) δ 189.32, 168.96, 150.74, 148.89, 148.75, 121.27, 112.82, 69.75, 62.64, 61.36, 56.66, 14.24.

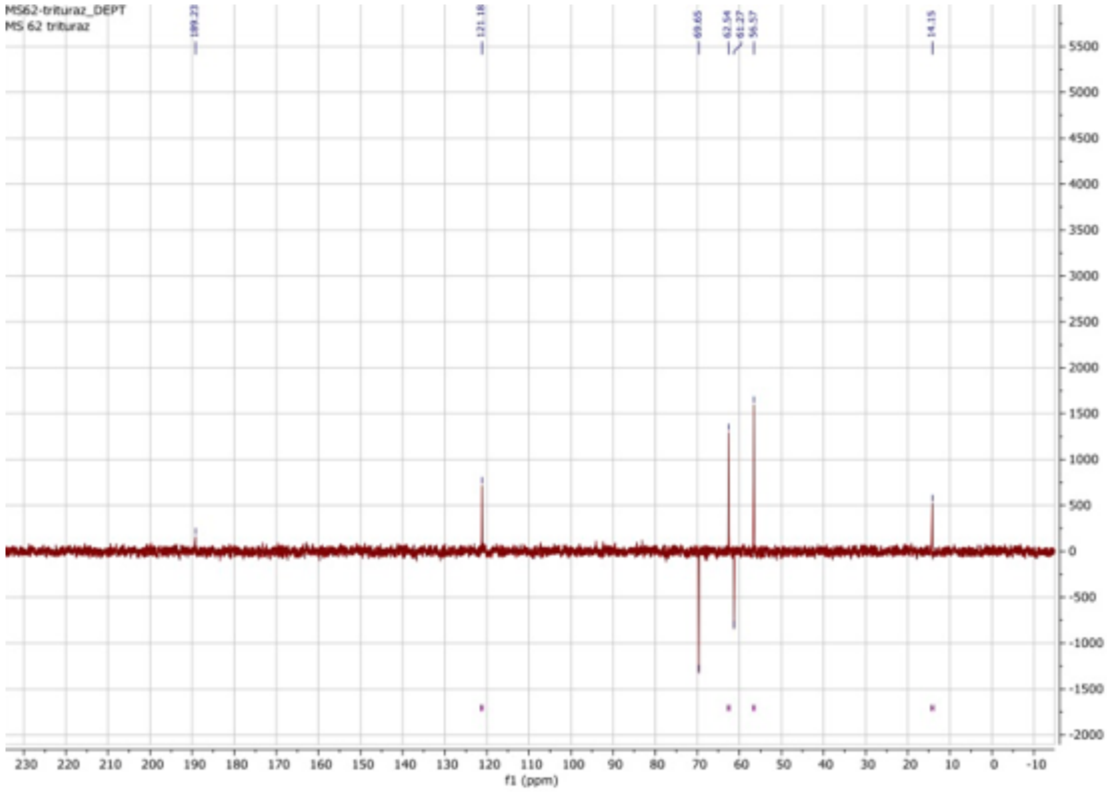

^{13}C NMR (101 MHz, Chloroform-d) δ 189.23, 121.18, 69.65, 62.54, 61.27, 56.57, 14.15.

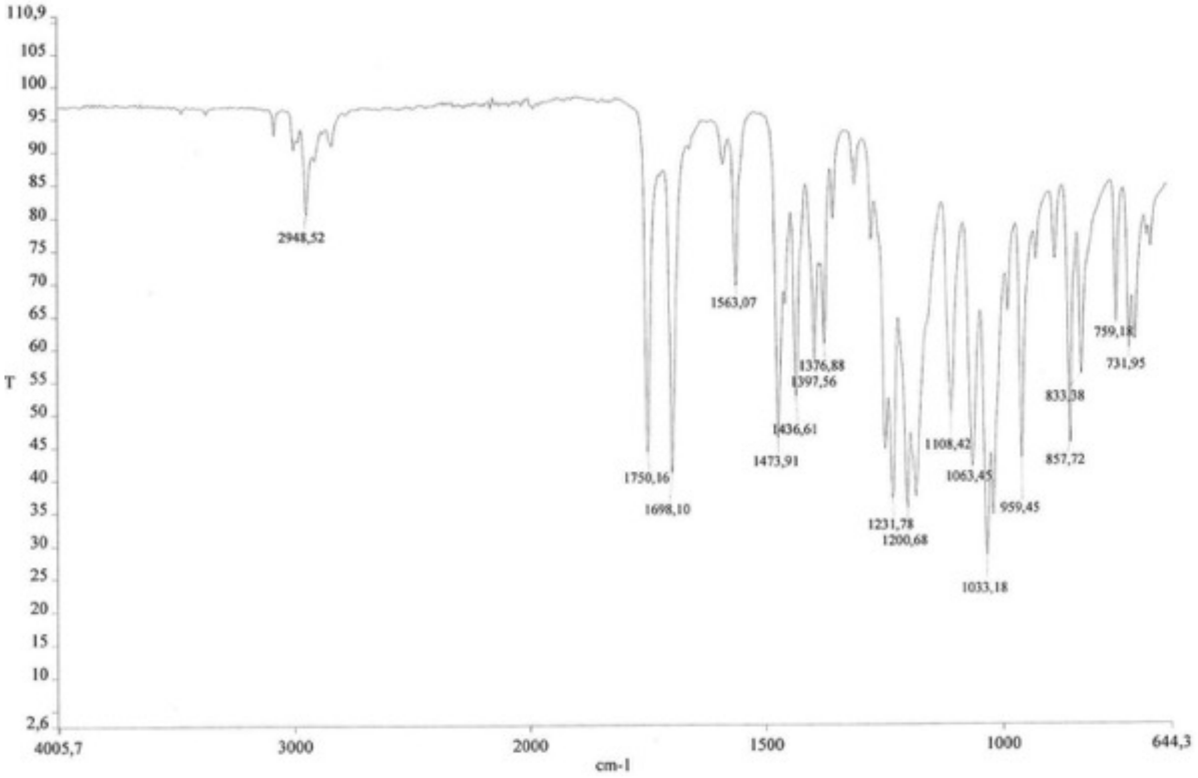

110,9
105
100
95
90
85
80
75
70
65
60
55
50
45
40
35
30
25
20
15
10
2,6
T
2948,52
1563,07
1750,16
1698,10
1473,91
1436,61
1397,56
1376,88
1231,78
1200,68
1108,42
1063,45
1033,18
959,45
857,72
833,38
759,18
731,95
4005,7
3000
2000
1500
1000
644,3
cm-1

2-(3-bromo-6-formyl-2,5-dimethoxyphenoxy)acetic acid

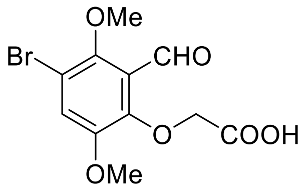

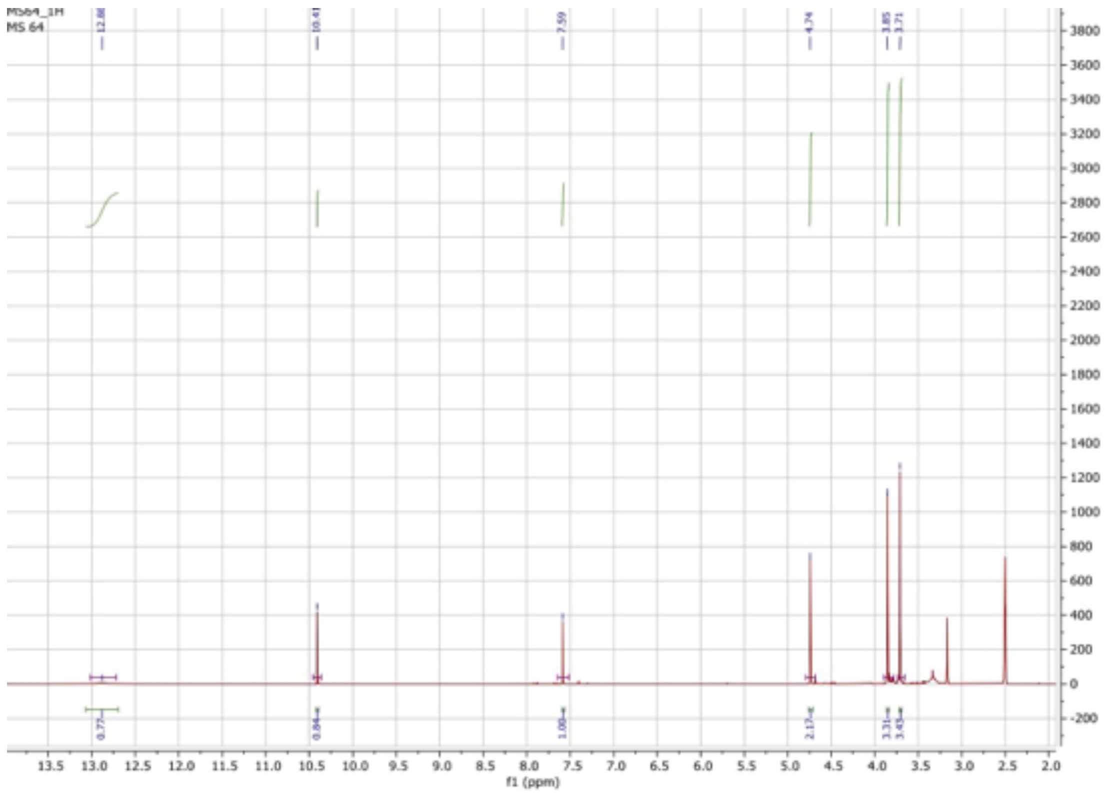

^{1}H NMR (400 MHz, DMSO-d_6) δ 12.88 (s, 1H), 10.41 (s, 1H), 7.59 (s, 1H), 4.74 (s, 2H), 3.85 (s, 3H), 3.71 (s, 3H).

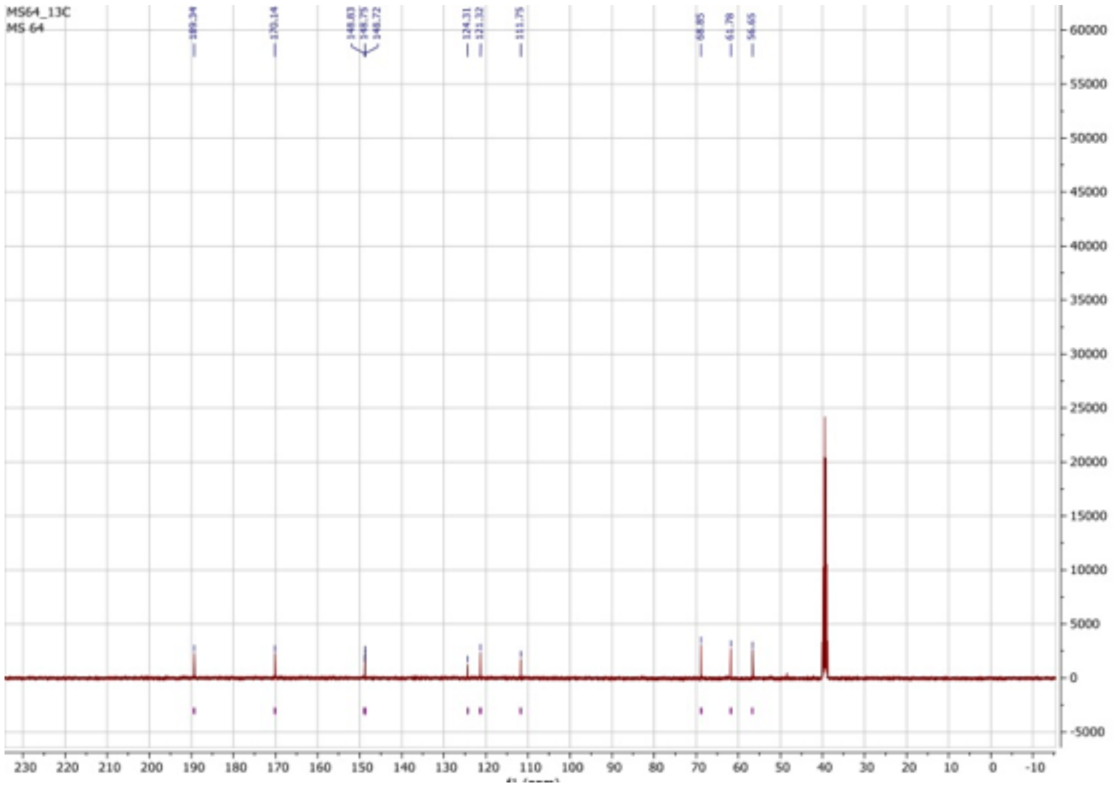

^{13}C NMR (101 MHz, dmso) δ 189.34, 170.14, 148.83, 148.75, 148.72, 124.31, 121.32, 111.75, 68.85, 61.78, 56.65.

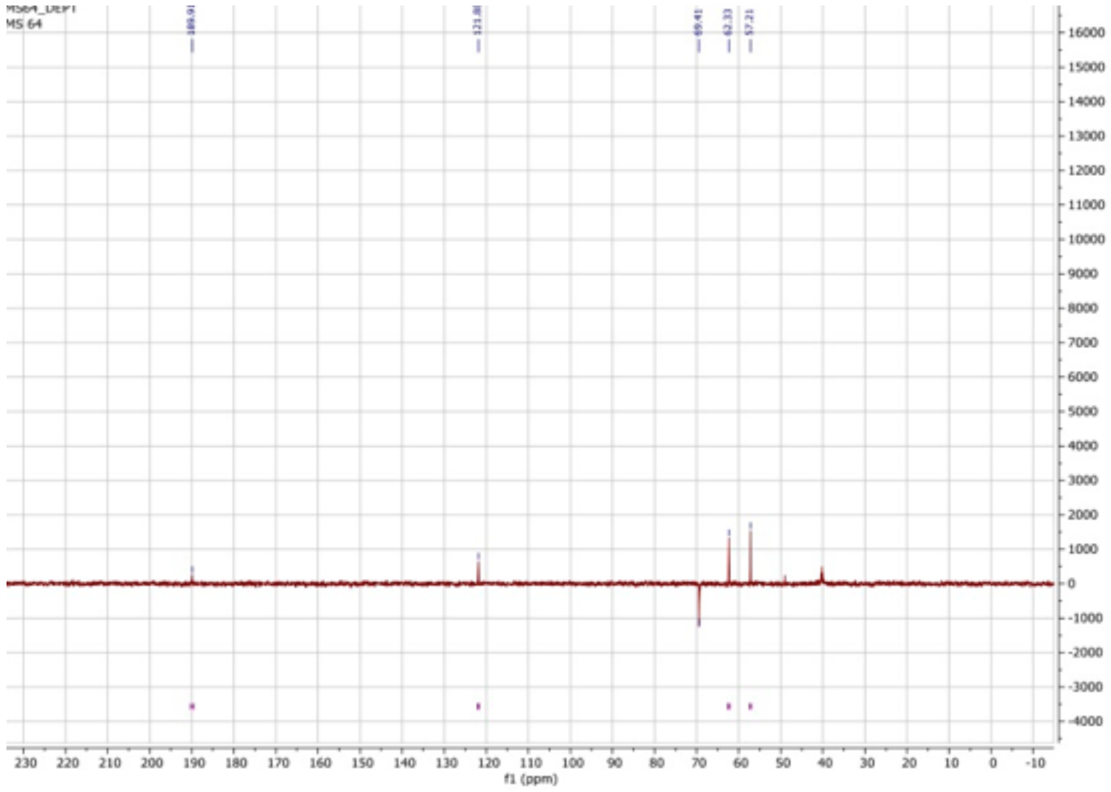

^{13}C NMR (101 MHz, dmso) δ 189.91, 121.88, 69.41, 62.33, 57.21.

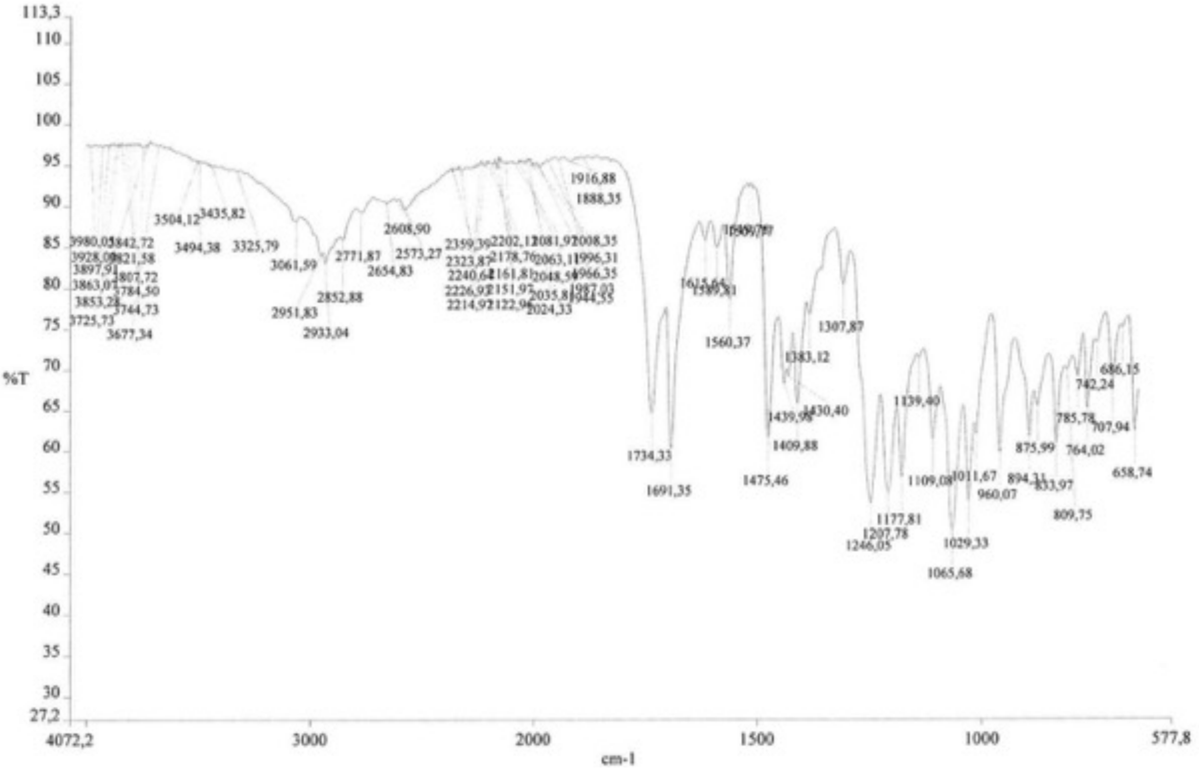

(E)-3-(4,7-dimethoxybenzofuran-5-yl)-1-phenylprop-2-en-1-one **(1)**

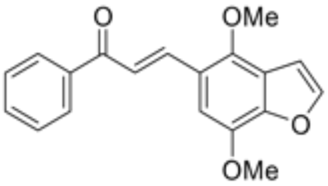

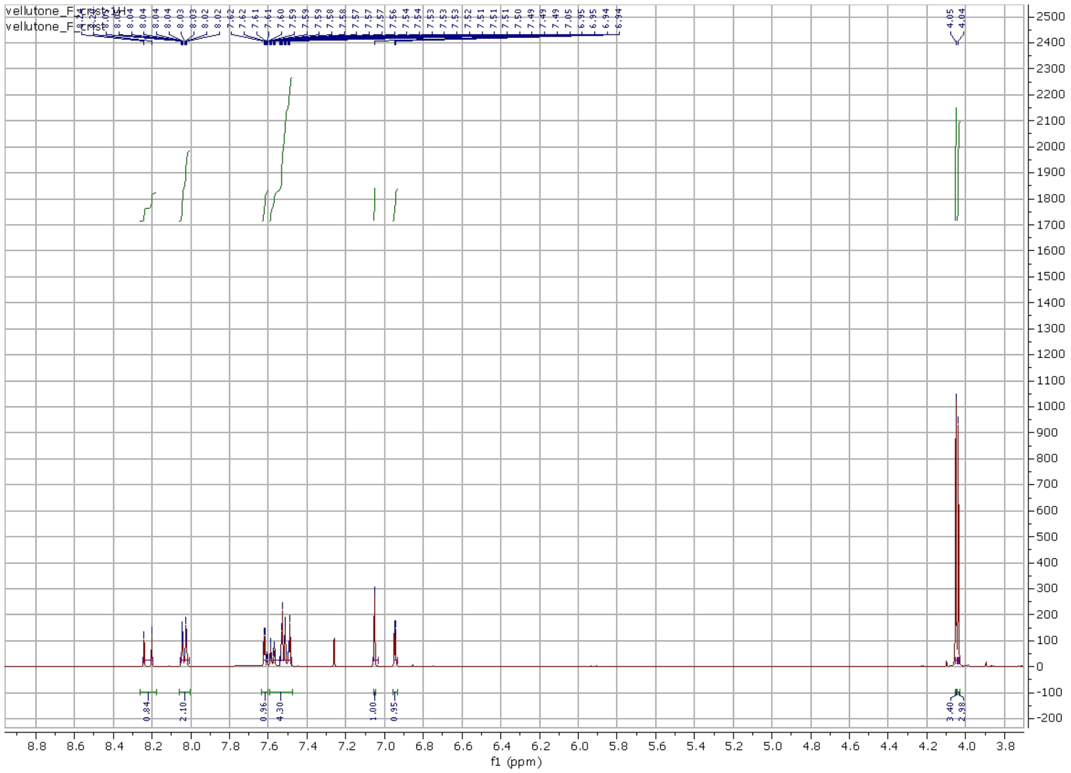

^{1}H NMR (400 MHz, Chloroform-*d*) δ 8.22 (d, *J* = 15.8 Hz, 1H), 8.05 – 8.01 (m, 2H), 7.62 (d, *J* = 2.2 Hz, 1H), 7.59 – 7.48 (m, 4H), 7.05 (s, 1H), 6.95 (dd, *J* = 2.2, 0.6 Hz, 1H), 4.05 (s, 3H), 4.04 (s, 3H).

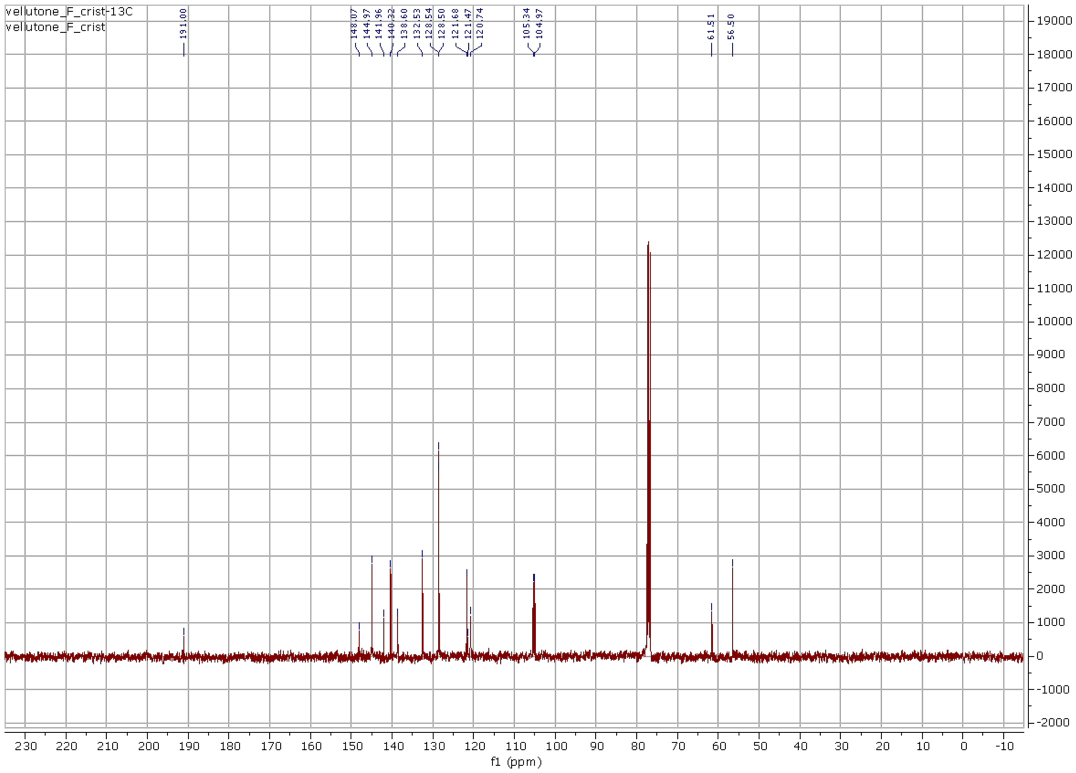

^{13}C NMR (101 MHz, Chloroform-d) δ 191.00, 148.07, 144.97, 141.96, 140.32, 138.60, 132.53, 128.54, 128.50, 121.68, 121.47, 120.74, 105.34, 104.97, 61.51, 56.50.

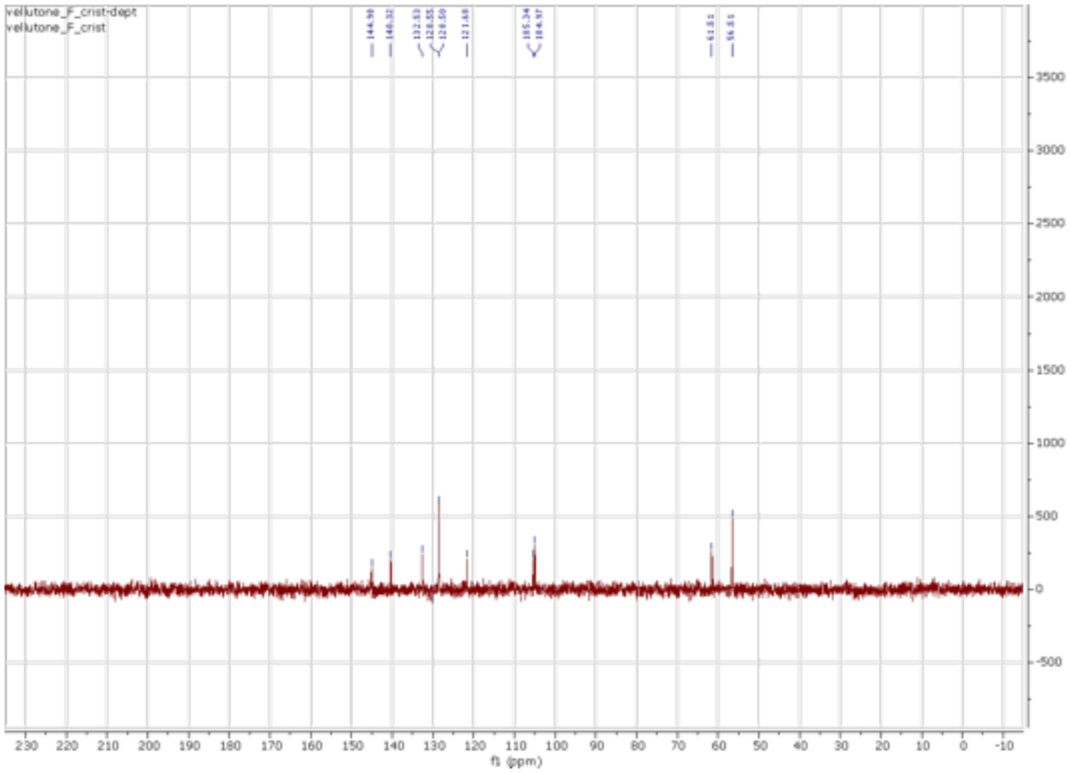

^{13}C NMR (101 MHz, Chloroform-d) δ 144.98, 140.32, 132.53, 128.55, 128.50, 121.68, 105.34, 104.97, 61.51, 56.51.

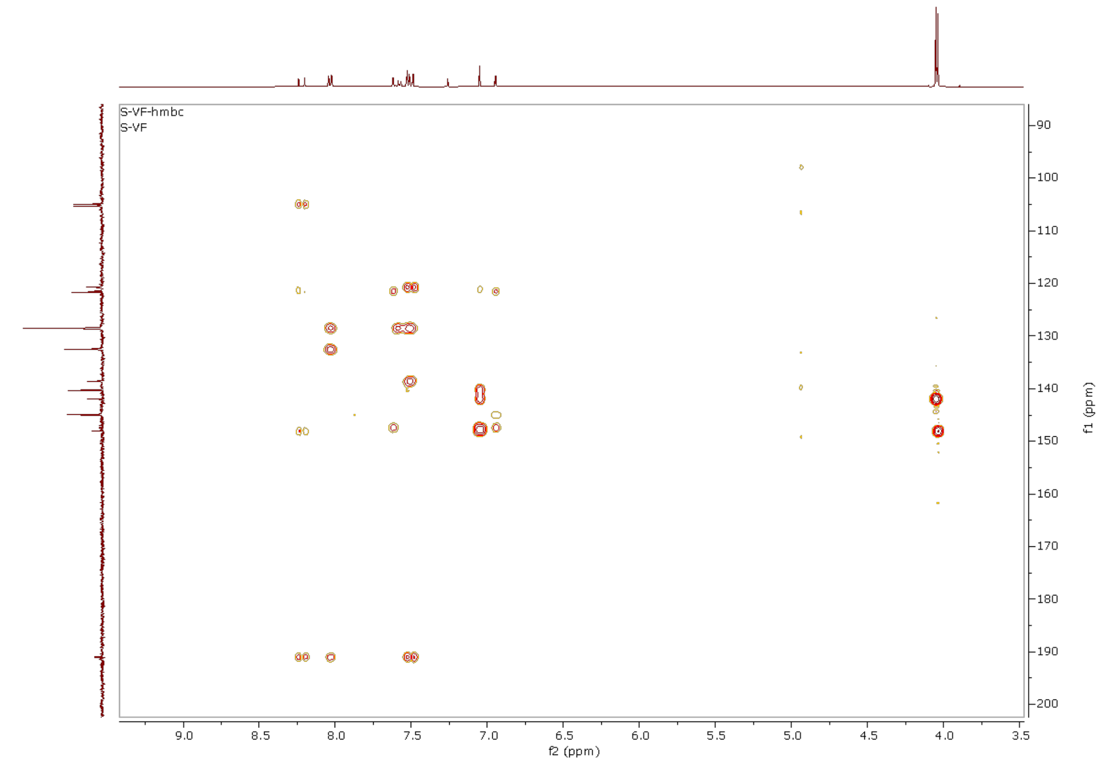

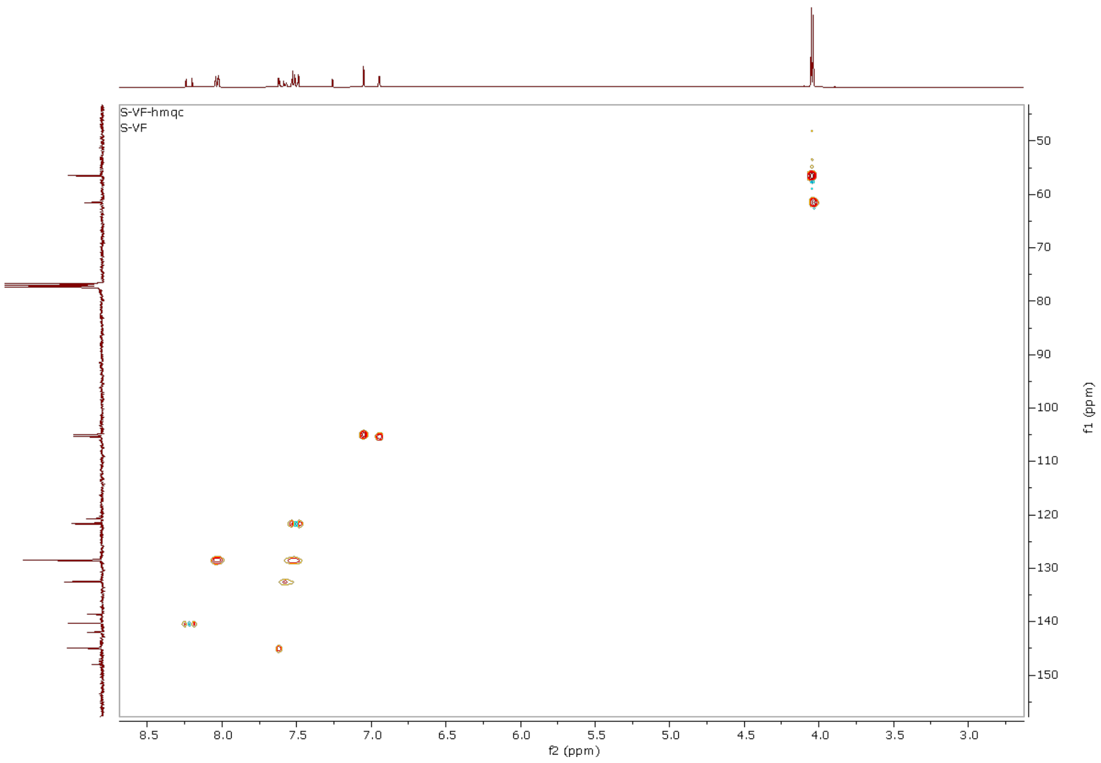

Area % Report

Data File: C:\32Karat\Projects\Default\Data\delia\Martina\vellutone F.dat
Method: C:\32Karat\Projects\Default\Method\standard 0.7 ml.met

Acquired: 05/05/2021 17.37.21
Printed: 04/08/2022 13.15.03

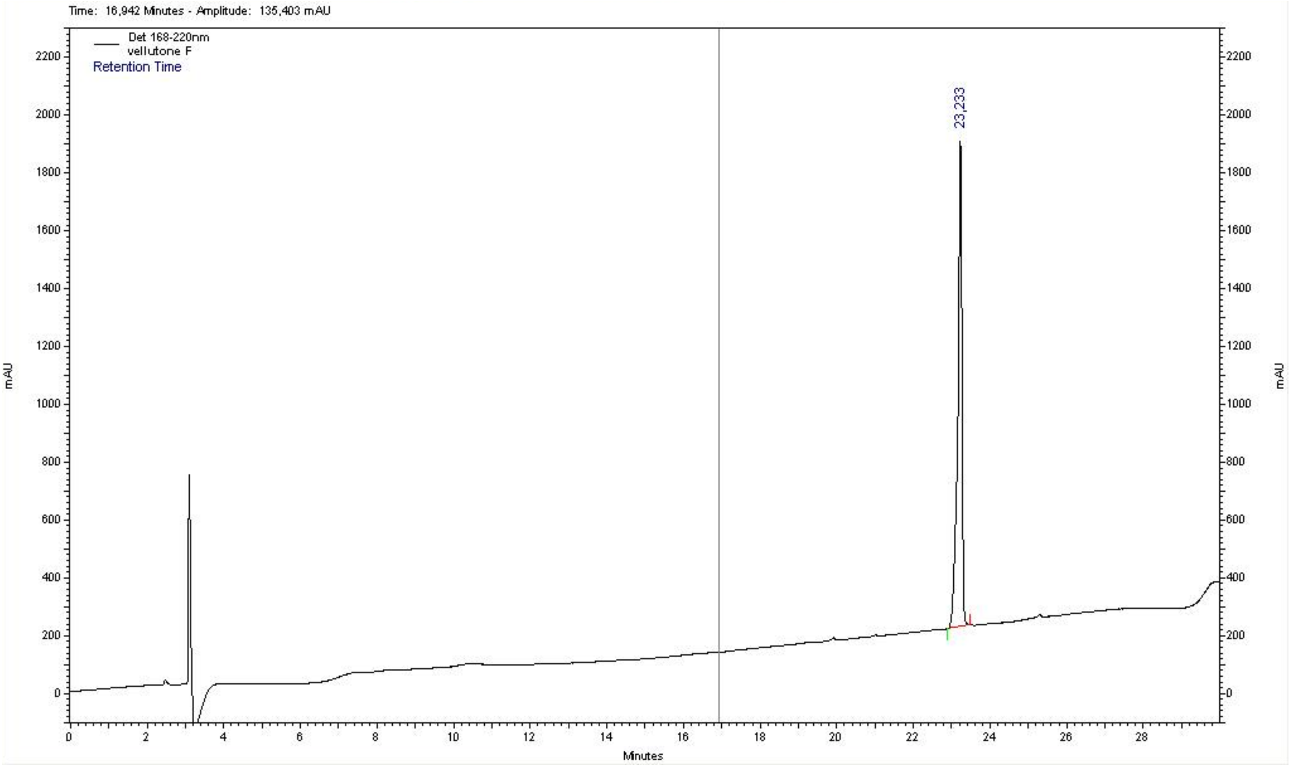

Det 168-220nm
Results

Time	Area	Area %	Height	Height %
23,233	12840863	100,00	1677712	100,00

Totals				
	12840863	100,00	1677712	100,00

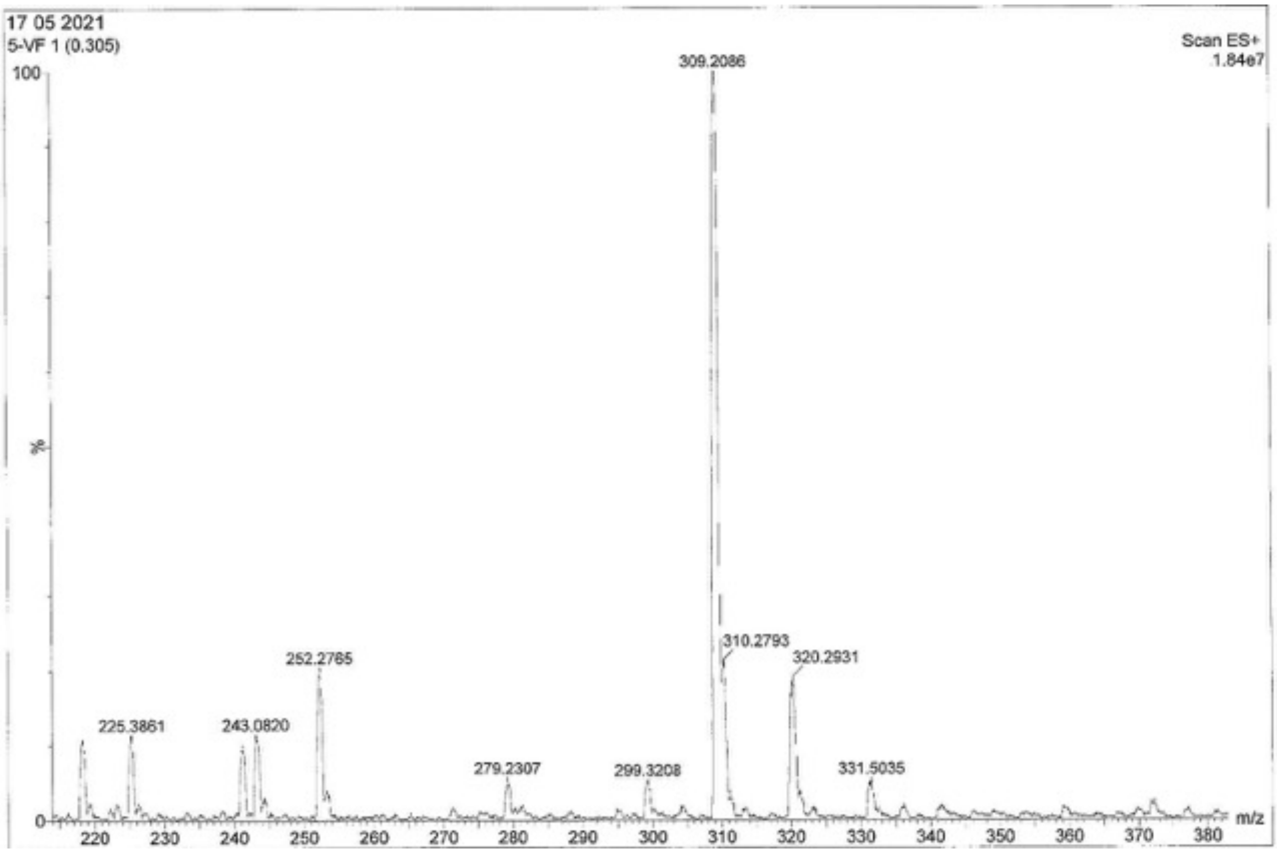
17 05 2021
5-VF 1 (0.305)
Scan ES+
1.84e7
309.2086
310.2793
320.2931
252.2765
225.3861
243.0820
279.2307
299.3208
331.5035
%
m/z

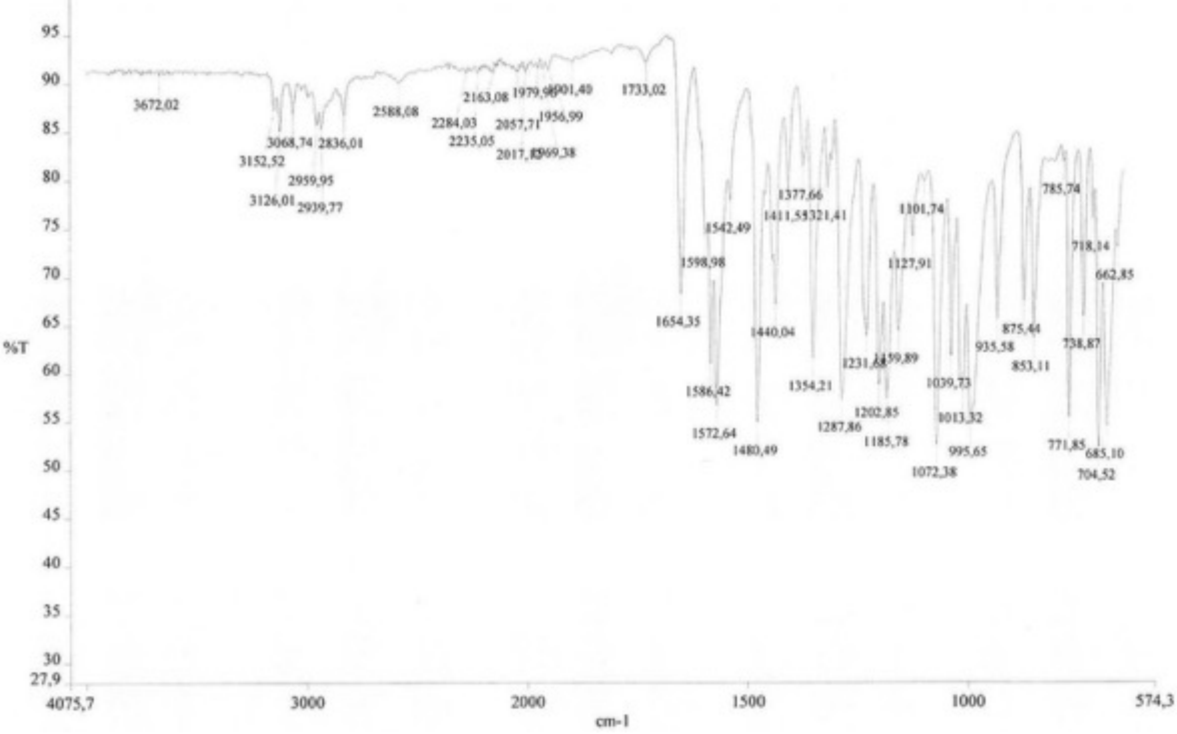
%T
cm-1
3672,02
1733,02
1654,35
1480,49
1072,38
704,52

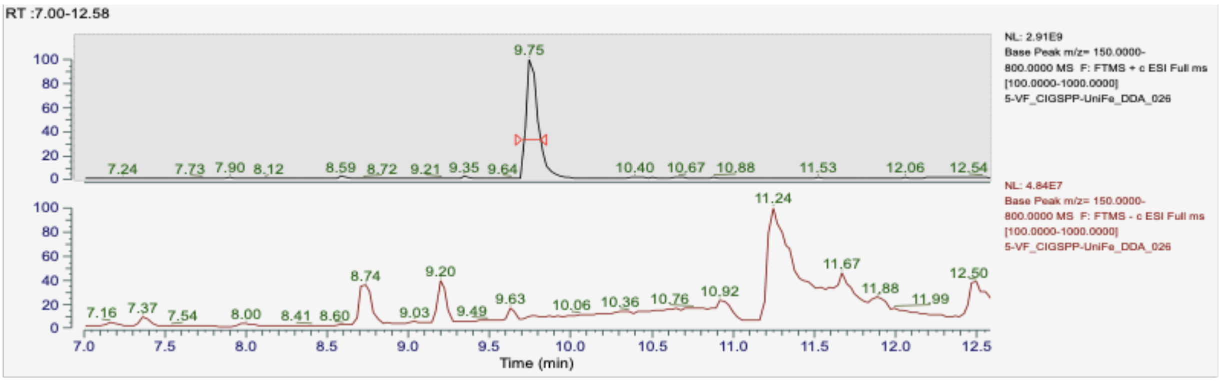

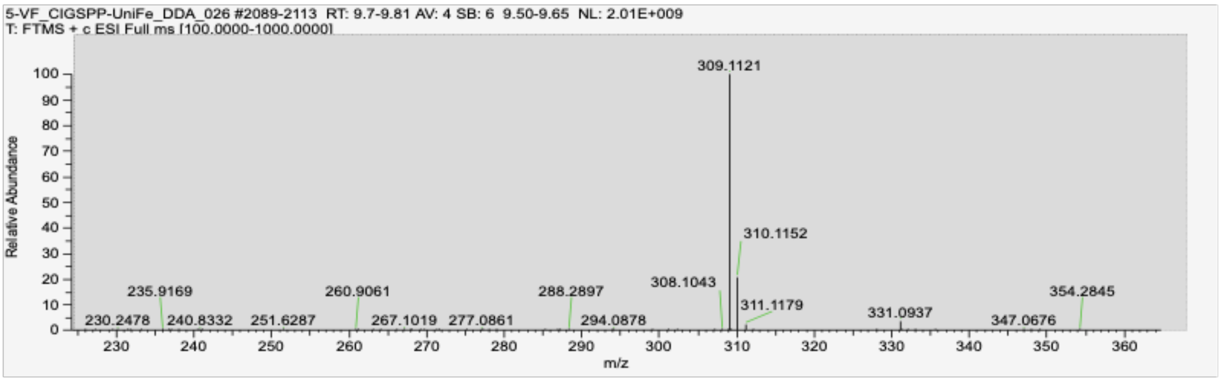

Peak Mass	Display Formula	Combined Fit	RDB	Delta [ppm]	Theo. mass	Rank	Combined Score	# Matched Iso.	# Missed Iso.	MS Cov. [%]	Pattern Cov. [%]
309,1121	$C_{19}H_{17}O_4$	24,8937856024	11,5	-0,15	309,11214	1	95,99	4	4	99,93	98,96
331,0937	$C_{19}H_{16}O_4{}^{23}Na$	17,1572498096	11,5	-1,02	331,09408	3	95,46	3	5	99,81	98,83

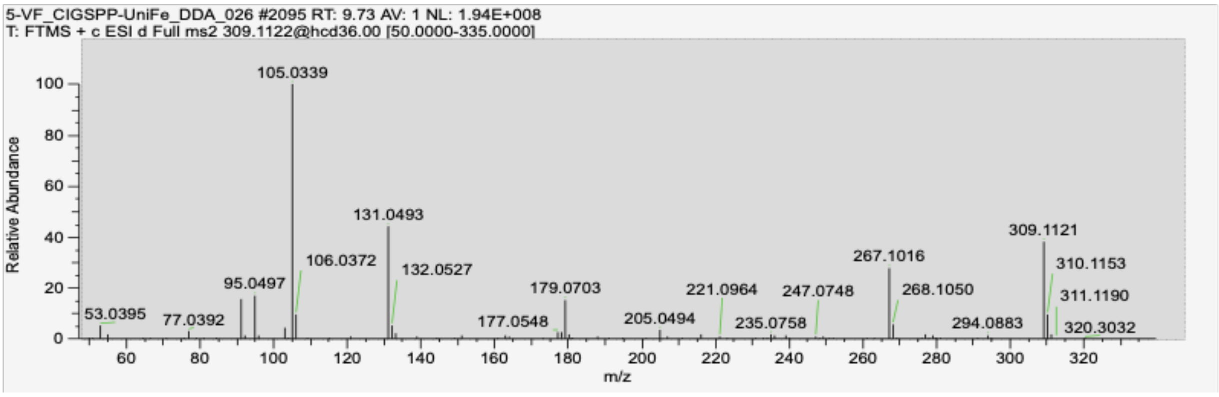

2-(2,2-dimethoxyethoxy)-1,4-dimethoxybenzene **(18)**

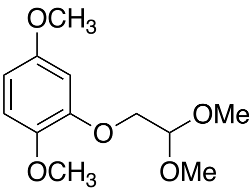

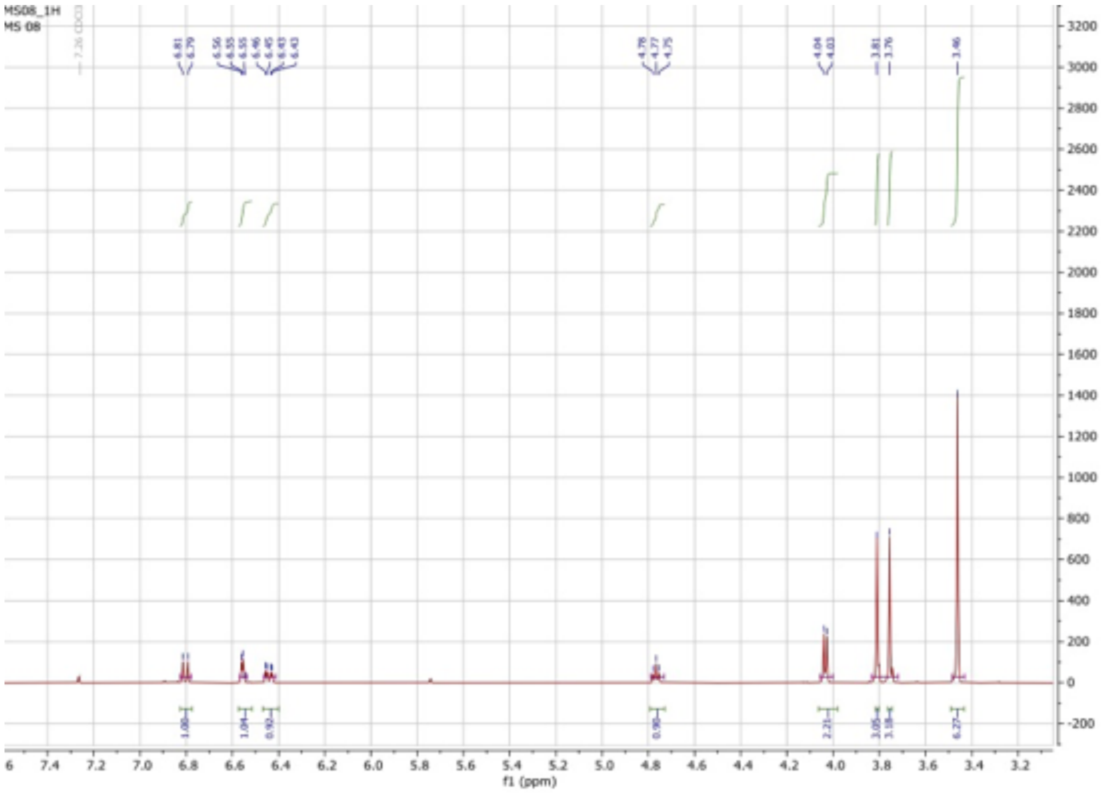

^{1}H NMR (400 MHz, Chloroform-*d*) δ 6.80 (d, *J* = 8.8 Hz, 1H), 6.56 (d, *J* = 2.9 Hz, 1H), 6.44 (dd, *J* = 8.8, 2.8 Hz, 1H), 4.77 (t, *J* = 5.2 Hz, 1H), 4.03 (d, *J* = 5.2 Hz, 2H), 3.78 (d, *J* = 21.9 Hz, 6H), 3.46 (s, 6H).

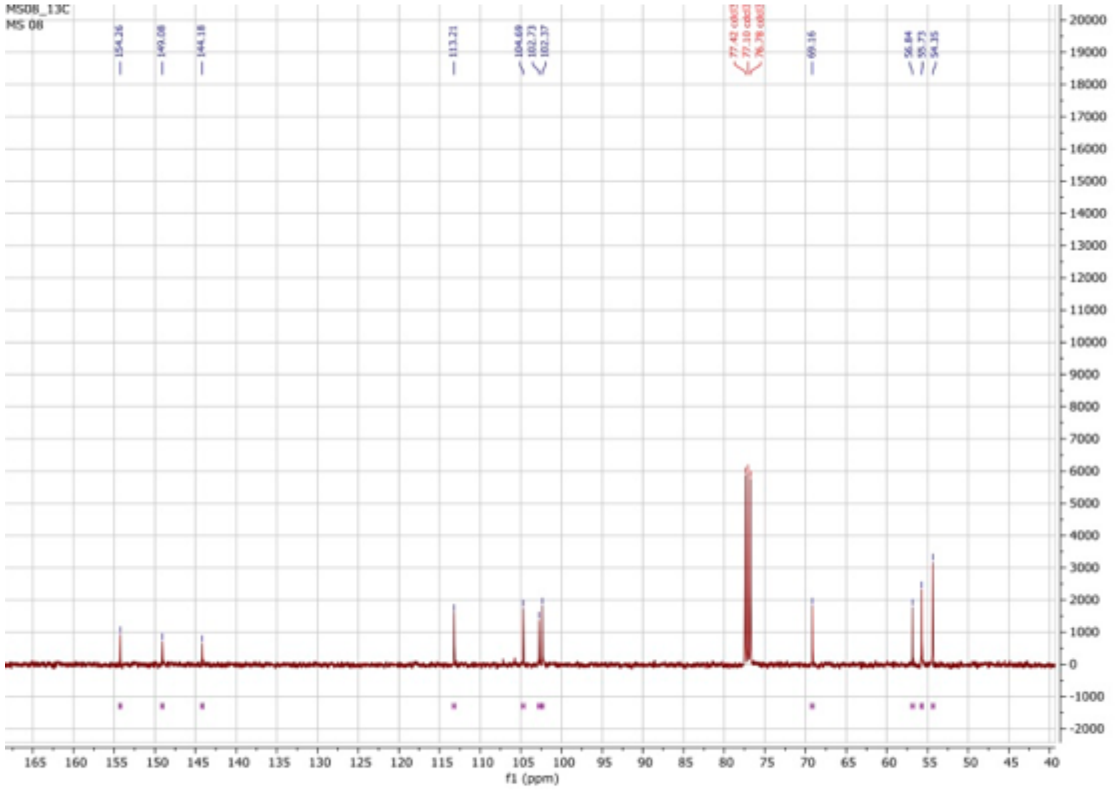

^{13}C NMR (101 MHz, Chloroform-d) δ 154.26, 149.08, 144.18, 113.21, 104.69, 102.73, 102.37, 69.16, 56.84, 55.73, 54.35.

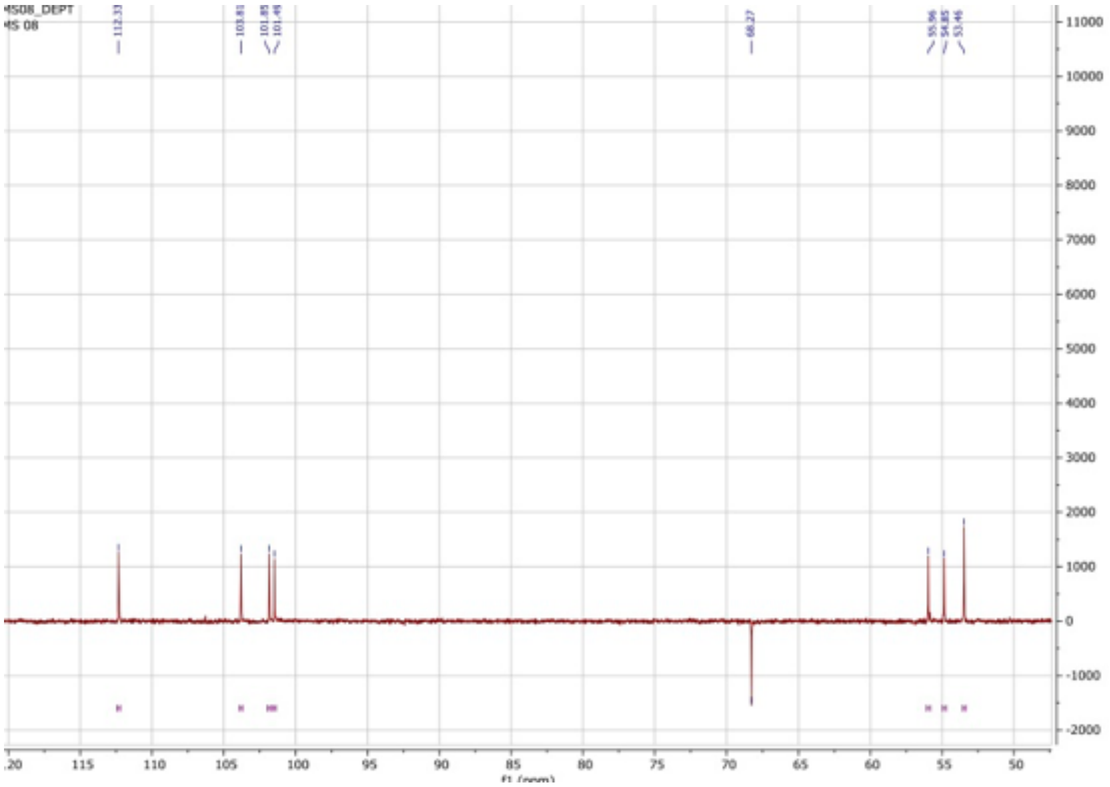

^{13}C NMR (101 MHz, Chloroform-d) δ 112.33, 103.81, 101.85, 101.49, 68.27, 55.96, 54.85, 53.46.

4,7-dimethoxybenzofuran **(19)**

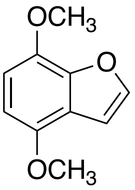

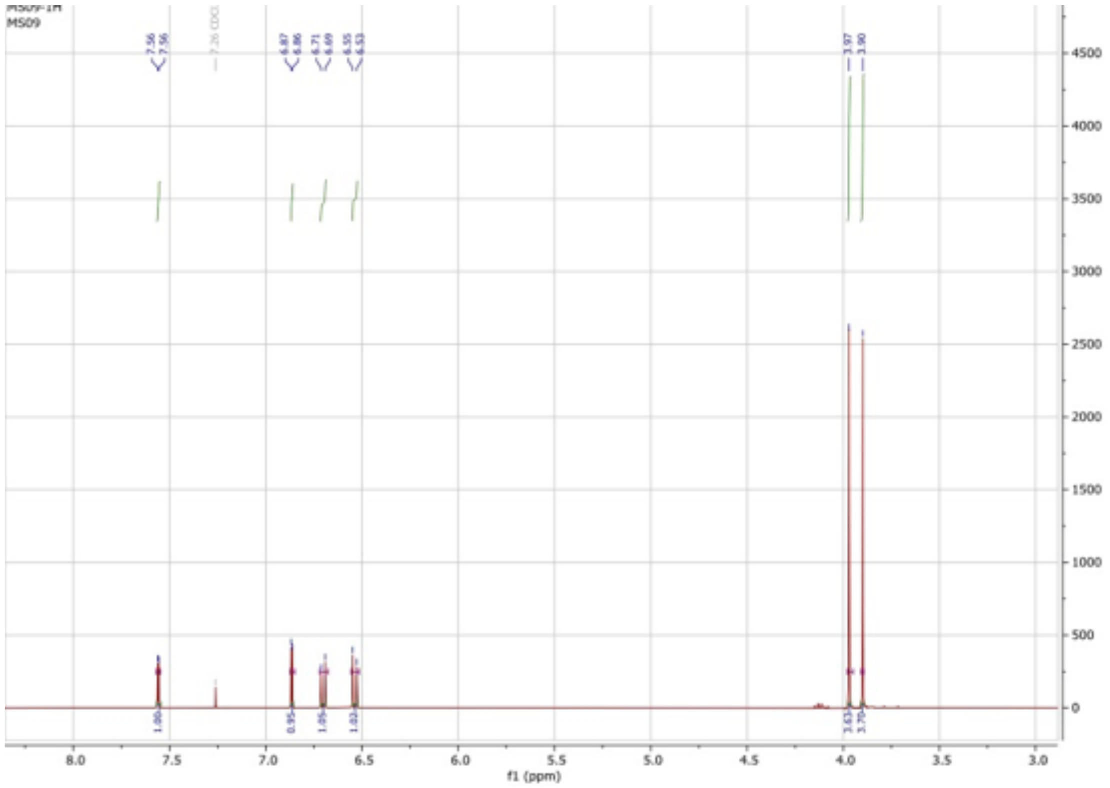

^{1}H NMR (400 MHz, Chloroform-*d*) δ 7.56 (d, *J* = 2.1 Hz, 1H), 6.86 (d, *J* = 2.1 Hz, 1H), 6.70 (d, *J* = 8.5 Hz, 1H), 6.54 (d, *J* = 8.5 Hz, 1H), 3.97 (s, 3H), 3.90 (s, 3H).

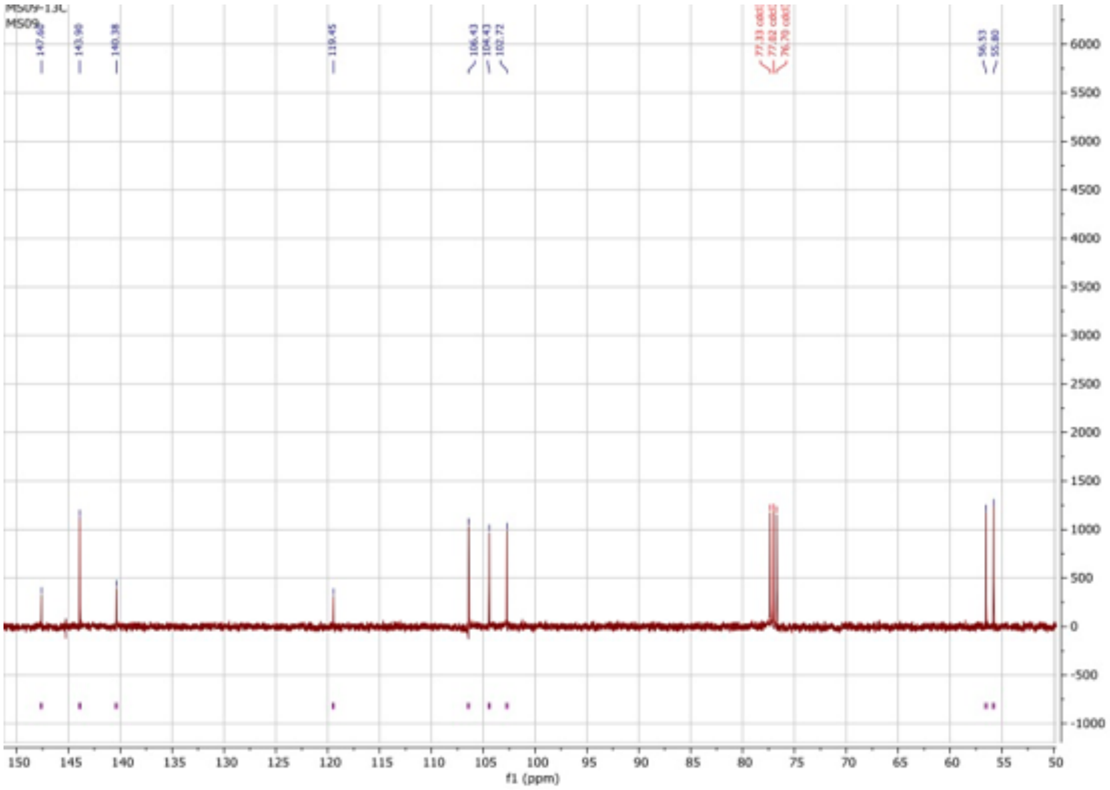

^{13}C NMR (101 MHz, Chloroform-d) δ 147.60, 143.90, 140.38, 119.45, 106.43, 104.43, 102.72, 56.53, 55.80.

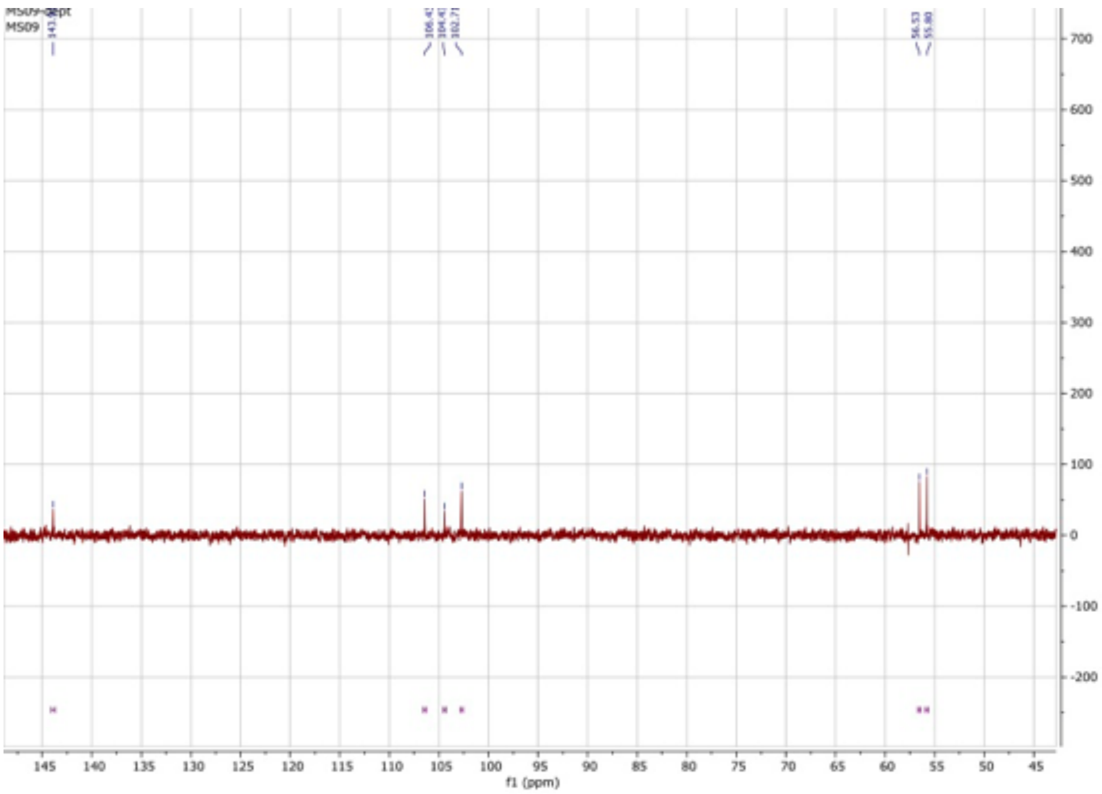

^{13}C NMR (101 MHz, Chloroform-d) δ 143.91, 106.43, 104.43, 102.71, 56.53, 55.80.

4,7-dimethoxybenzofuran-2-carbaldehyde **(20)** + 4,7-dimethoxybenzofuran-5-carbaldehyde **(7)**

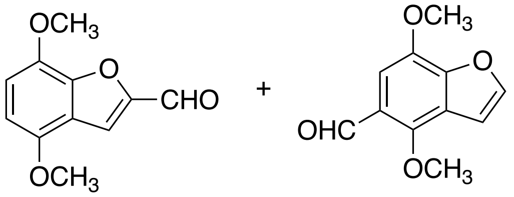

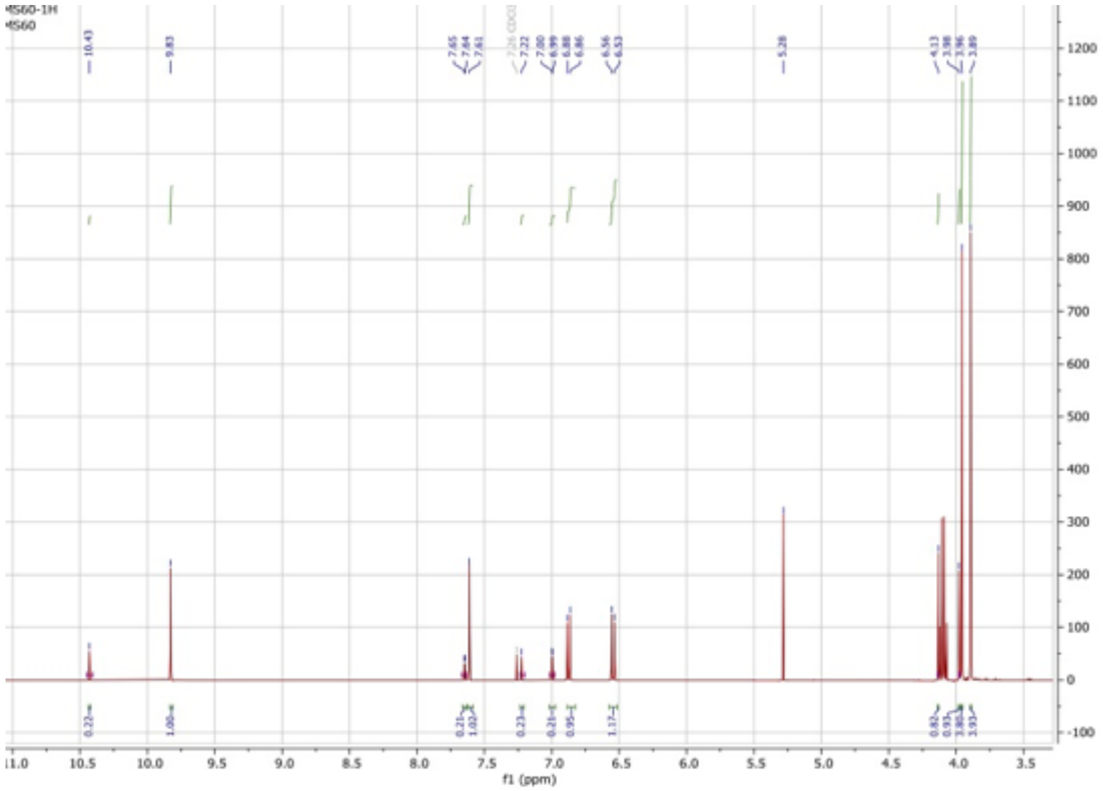

15 ^{1}H NMR (400 MHz, Chloroform-*d*) δ 10.43 (s, 1H), 7.65 (d, *J* = 2.3 Hz, 1H), 7.22 (s, 1H), 7.00 (d, *J* = 2.3 Hz, 1H), 4.13 (s, 3H), 3.98 (s, 3H).

16 ^{1}H NMR (400 MHz, Chloroform-*d*) δ 9.83 (s, 1H), 7.61 (s, 1H), 6.87 (d, *J* = 8.5 Hz, 1H), 6.54 (d, *J* = 8.6 Hz, 1H), 3.96 (s, 3H), 3.89 (s, 3H).

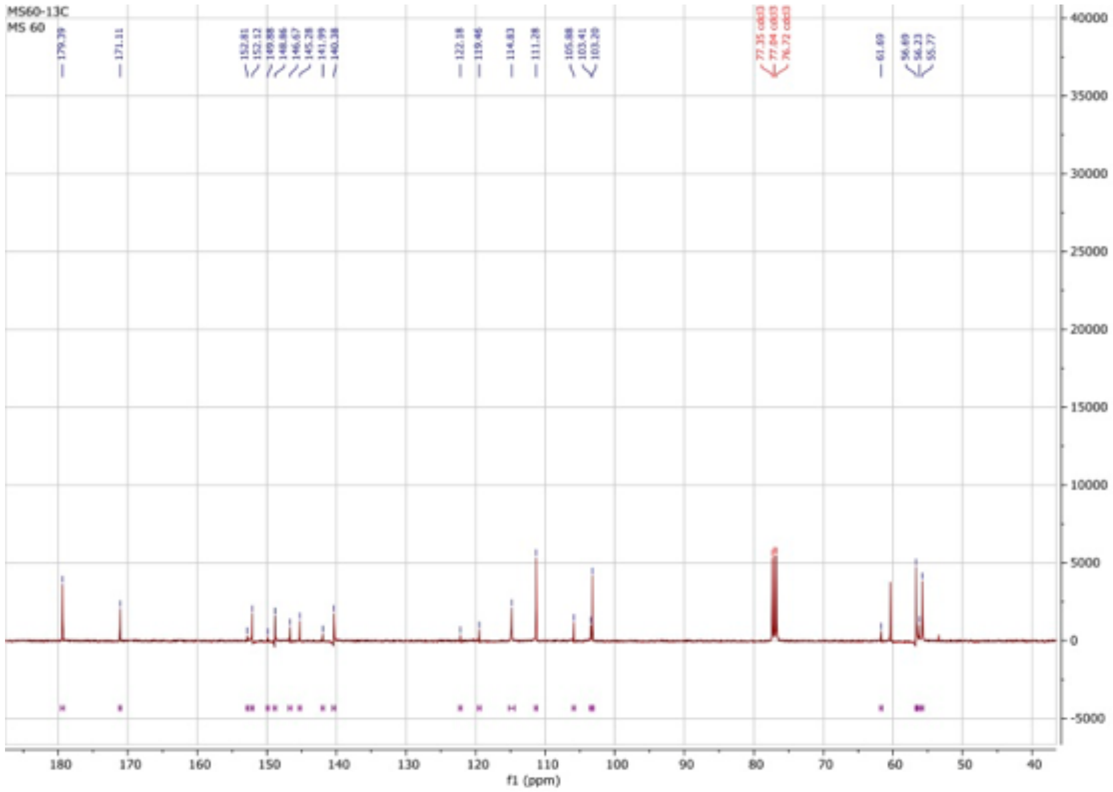

^{13}C NMR (101 MHz, Chloroform-d) δ 189.05, 179.39, 171.11, 152.81, 152.12, 149.88, 148.86, 146.67, 145.28, 141.99, 140.38, 122.18, 119.46, 114.83, 111.28, 105.88, 103.41, 103.20, 77.35, 77.04, 76.72, 61.69, 56.69, 56.23, 55.77.

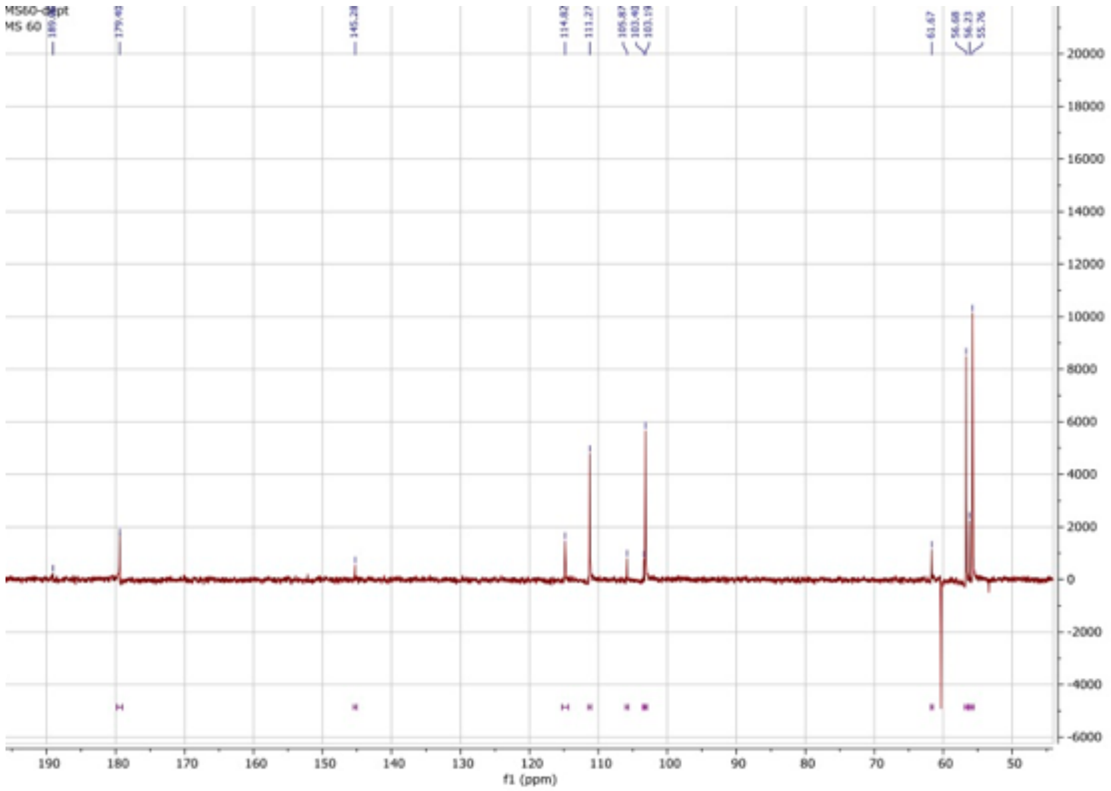

^{13}C NMR (101 MHz, Chloroform-d) δ 189.06, 179.40, 145.28, 114.82, 111.27, 105.87, 103.40, 103.19, 61.67, 56.68, 56.23, 55.76.

Area % Report

Data File: C:\32Karat\Projects\Default\Data\delia\Martina\2-5dimetossibenzofurano.dat
Method: C:\32Karat\Projects\Default\Method\standard 0.7 ml.met
Acquired: 01/04/2021 12.52.29
Printed: 04/08/2022 13.17.44

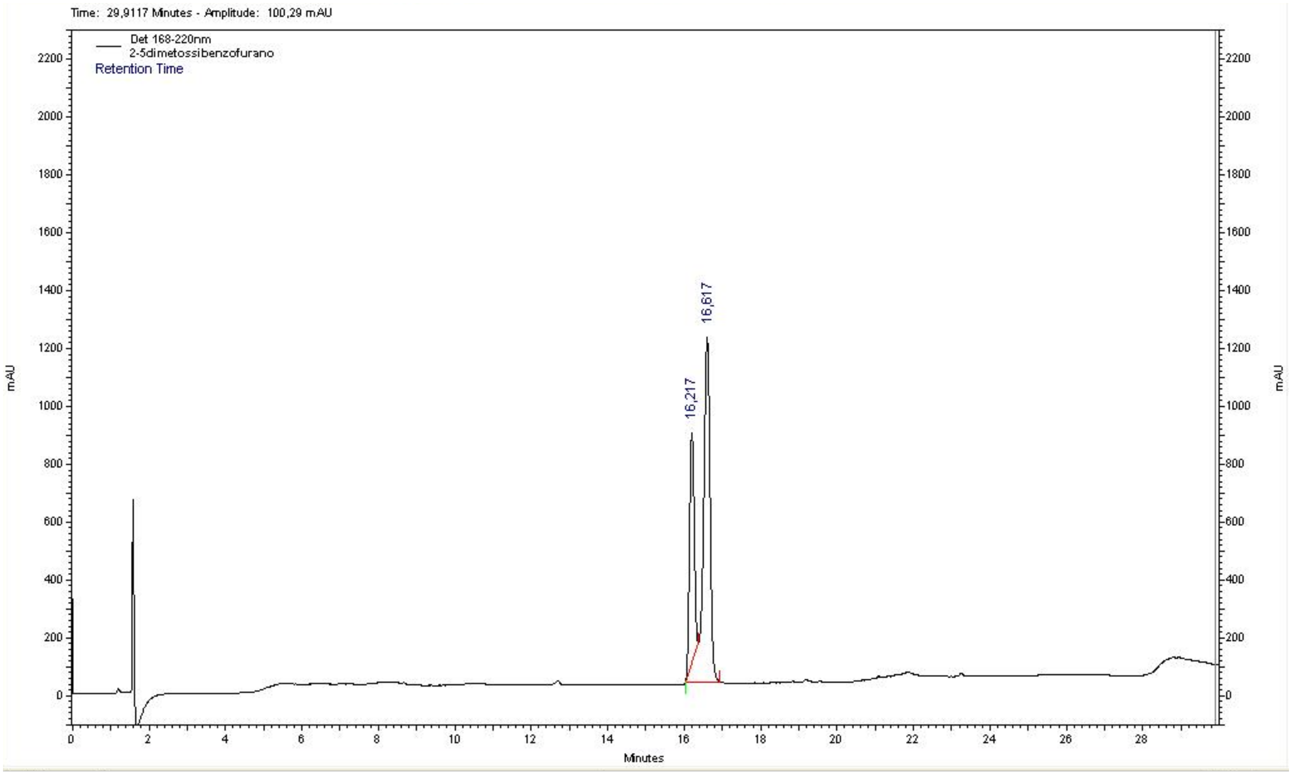

Det 168-220nm
Results

Time	Area	Area %	Height	Height %
16,217	6780940	30,65	796575	40,05
16,617	15339907	69,35	1192260	59,95
Totals	22120847	100,00	1988835	100,00

4,7-dimethoxybenzofuran-6-carbaldehyde **(21)**

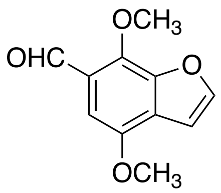

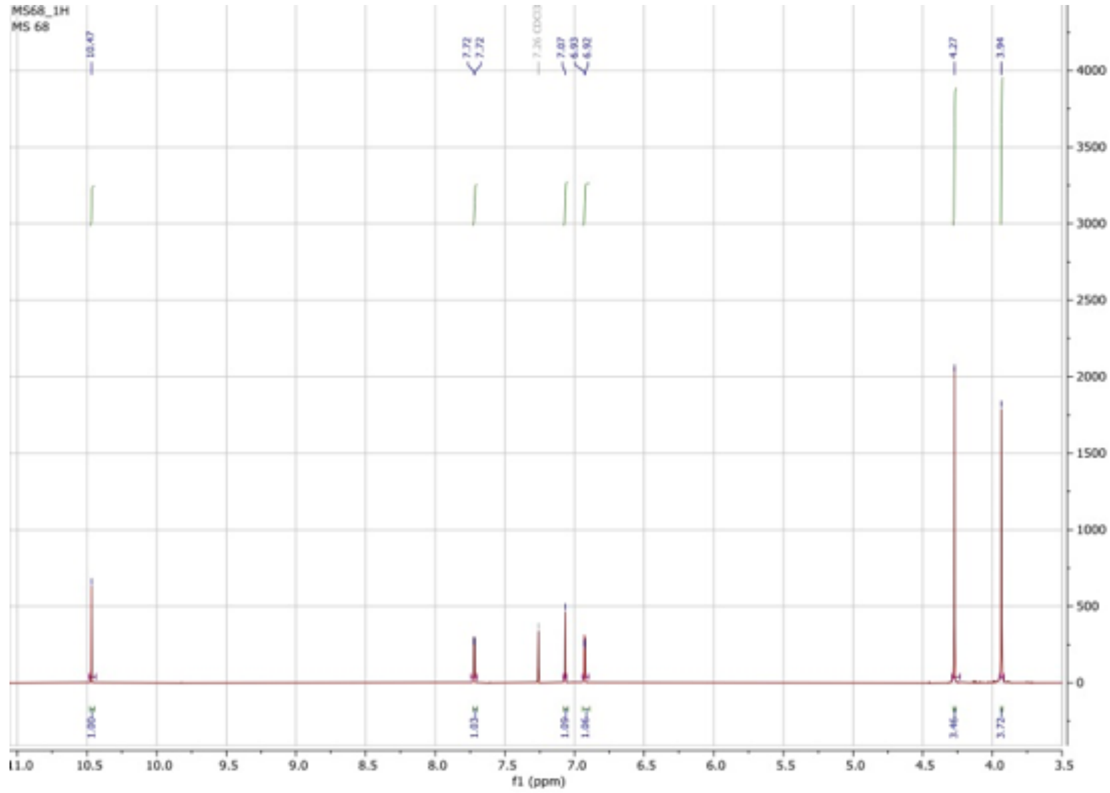

^{1}H NMR (400 MHz, Chloroform-*d*) δ 10.47 (s, 1H), 7.72 (d, *J* = 2.1 Hz, 1H), 7.07 (s, 1H), 6.92 (d, *J* = 2.1 Hz, 1H), 4.27 (s, 3H), 3.94 (s, 3H).

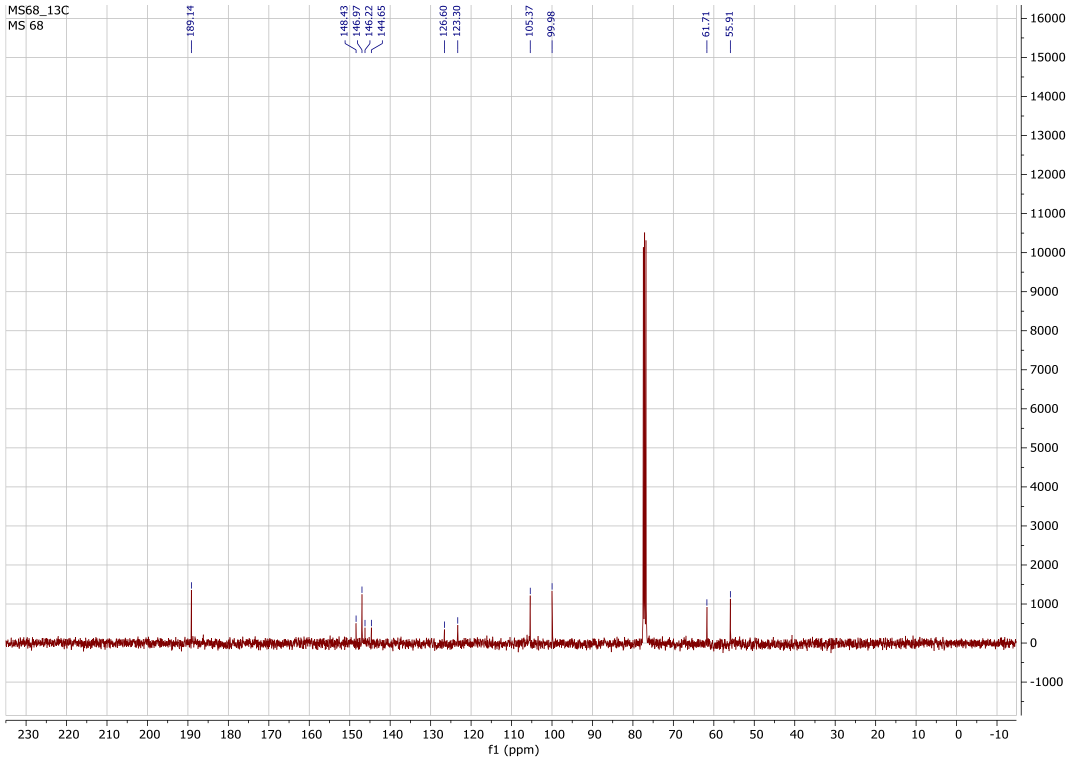

^{13}C NMR (101 MHz, Chloroform-d) δ 189.14, 148.43, 146.97, 146.22, 144.65, 126.60, 123.30, 105.37, 99.98, 61.71, 55.91.

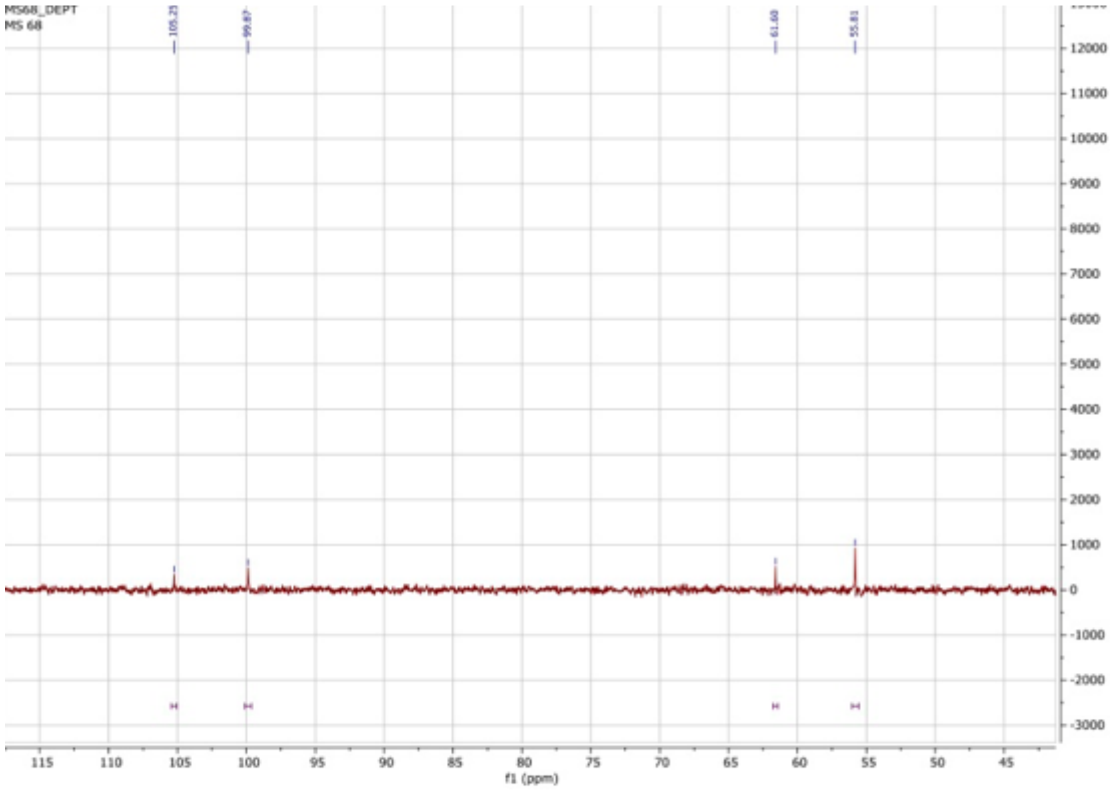

^{13}C NMR (101 MHz, Chloroform-d) δ 105.25, 99.87, 61.60, 55.81.

1-(2.5-dimethoxyphenoxy)propan-2-one **(24)**

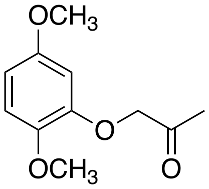

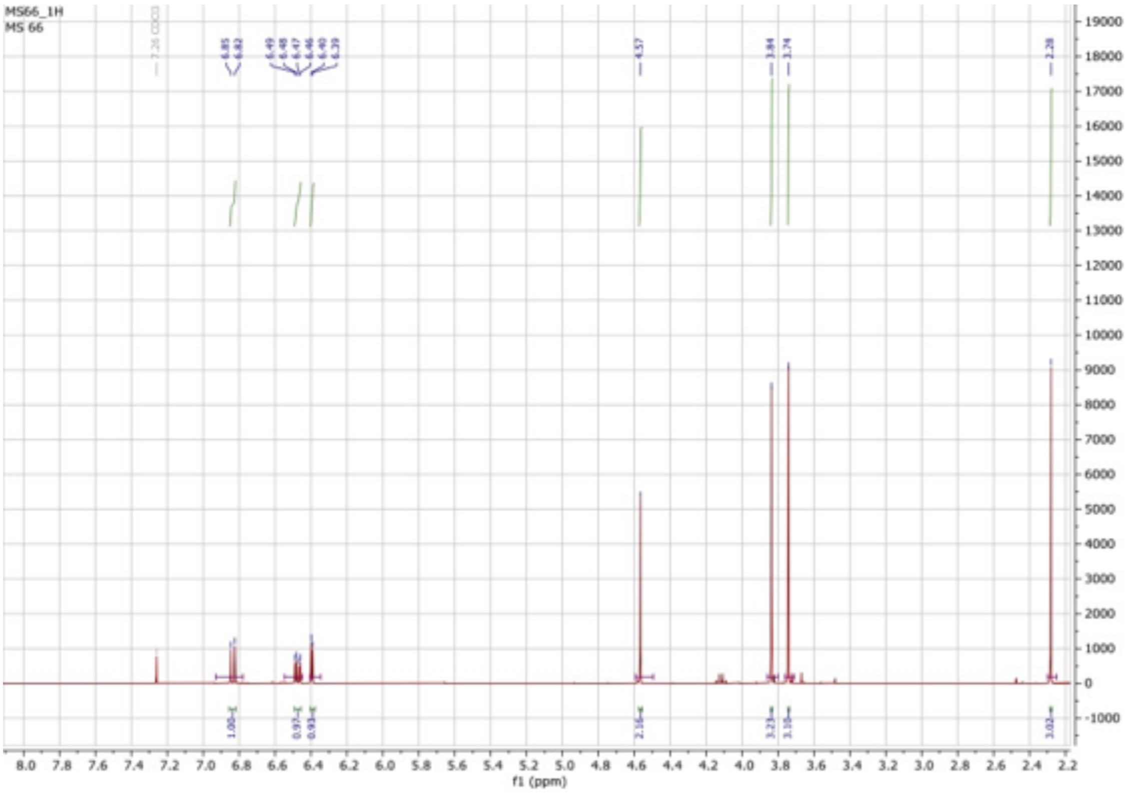

^{1}H NMR (400 MHz, Chloroform-d) δ 6.83 (d, J = 8.8 Hz, 1H), 6.47 (dd, J = 8.8, 2.8 Hz, 1H), 6.39 (d, J = 2.8 Hz, 1H), 4.57 (s, 2H), 3.84 (s, 3H), 3.74 (s, 3H), 2.28 (s, 3H).

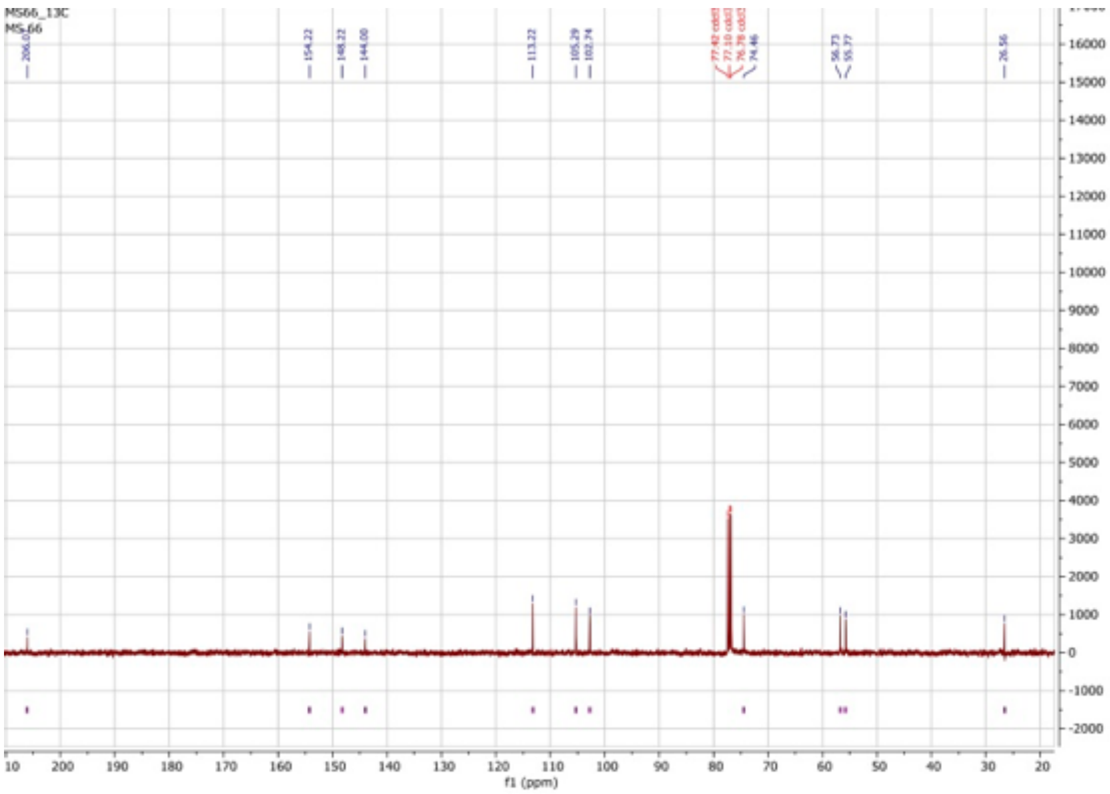

^{13}C NMR (101 MHz, Chloroform-d) δ 206.07, 154.22, 148.22, 144.00, 113.22, 105.29, 102.74, 74.46, 56.73, 55.77, 26.56.

.

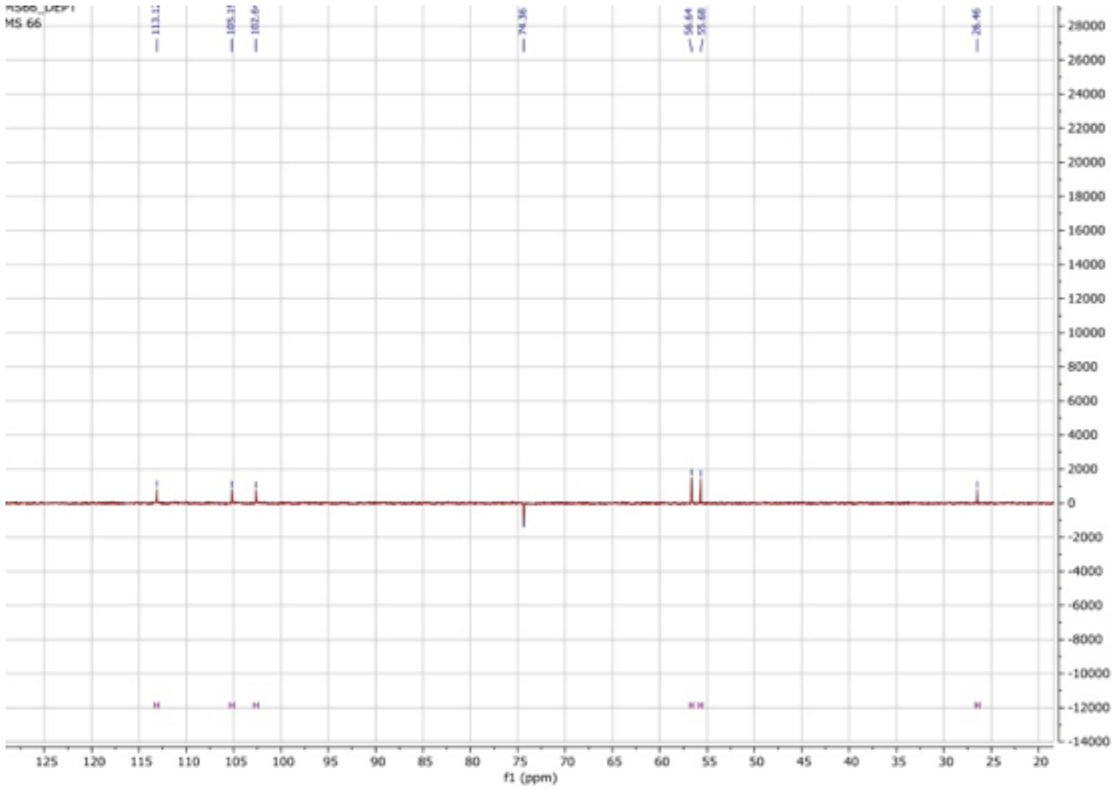

^{13}C NMR (101 MHz, Chloroform-d) δ 113.12, 105.19, 102.64, 74.36, 56.64, 55.68, 26.46.

4,7-dimethoxy-3-methylbenzofuran **(25)**

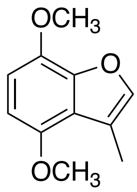

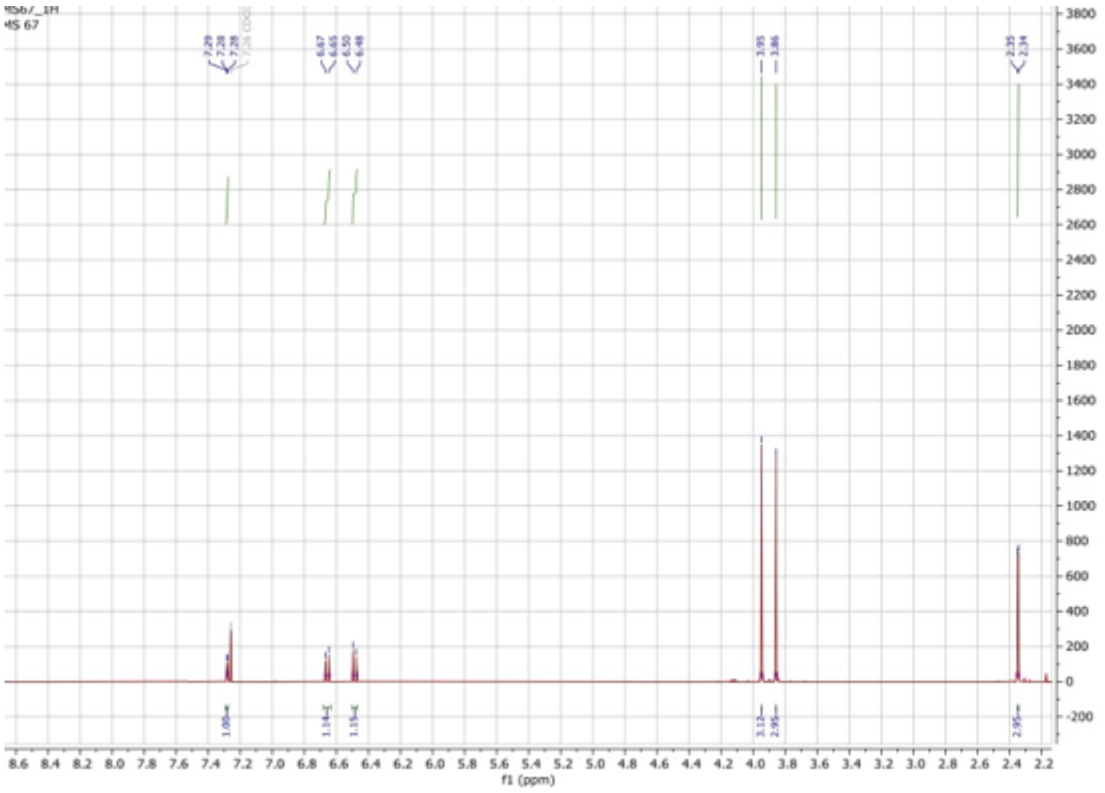

^{1}H NMR (400 MHz, Chloroform-d) δ 7.28 (d, *J* = 1.4 Hz, 1H), 6.66 (d, *J* = 8.5 Hz, 1H), 6.49 (d, *J* = 8.5 Hz, 1H), 3.95 (s, 3H), 3.86 (s, 3H), 2.35 (d, *J* = 1.3 Hz, 3H).

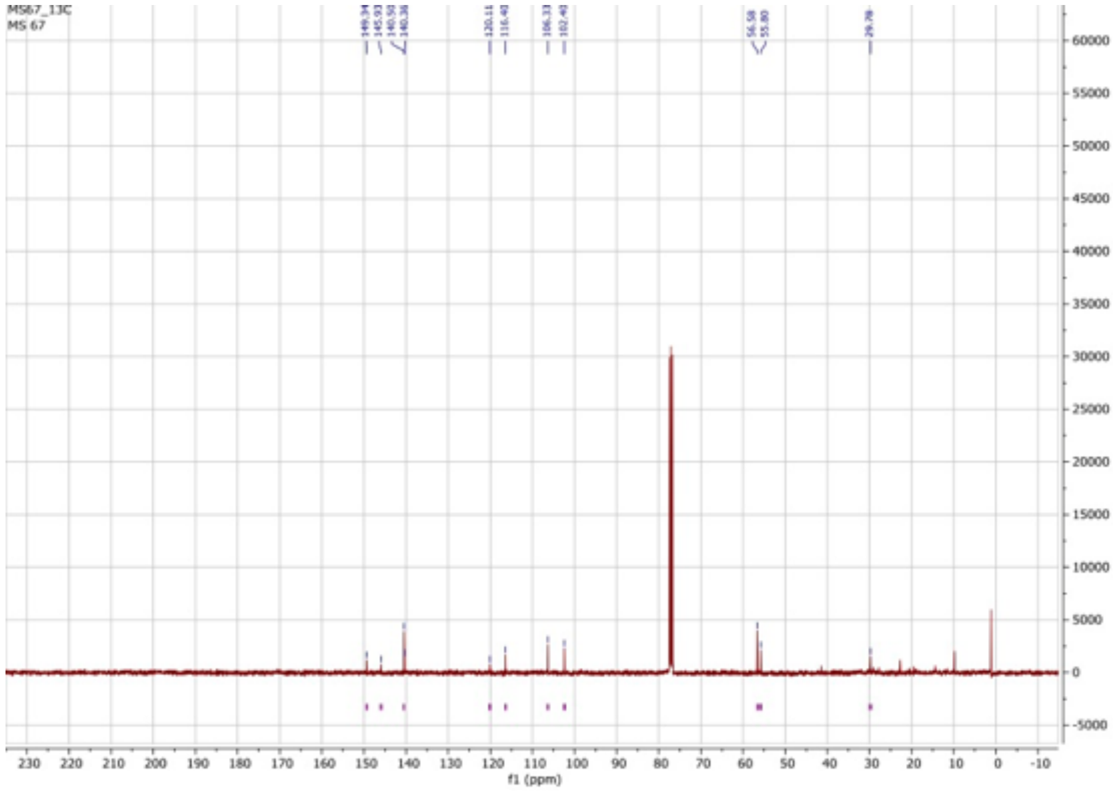

^{13}C NMR (101 MHz, Chloroform-d) δ 149.34, 145.93, 140.50, 140.36, 120.11, 116.40, 106.33, 102.40, 56.58, 55.80, 29.78.

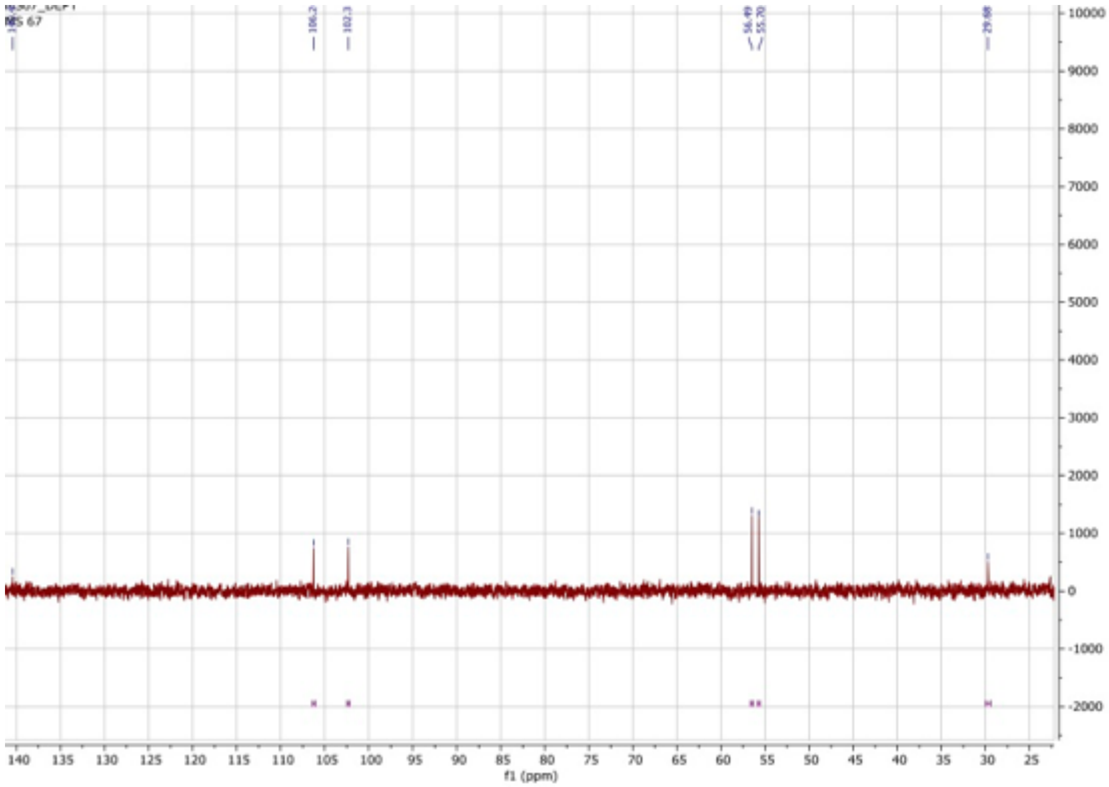

^{13}C NMR (101 MHz, Chloroform-d) δ 140.41, 106.24, 102.31, 56.49, 55.70, 29.68.

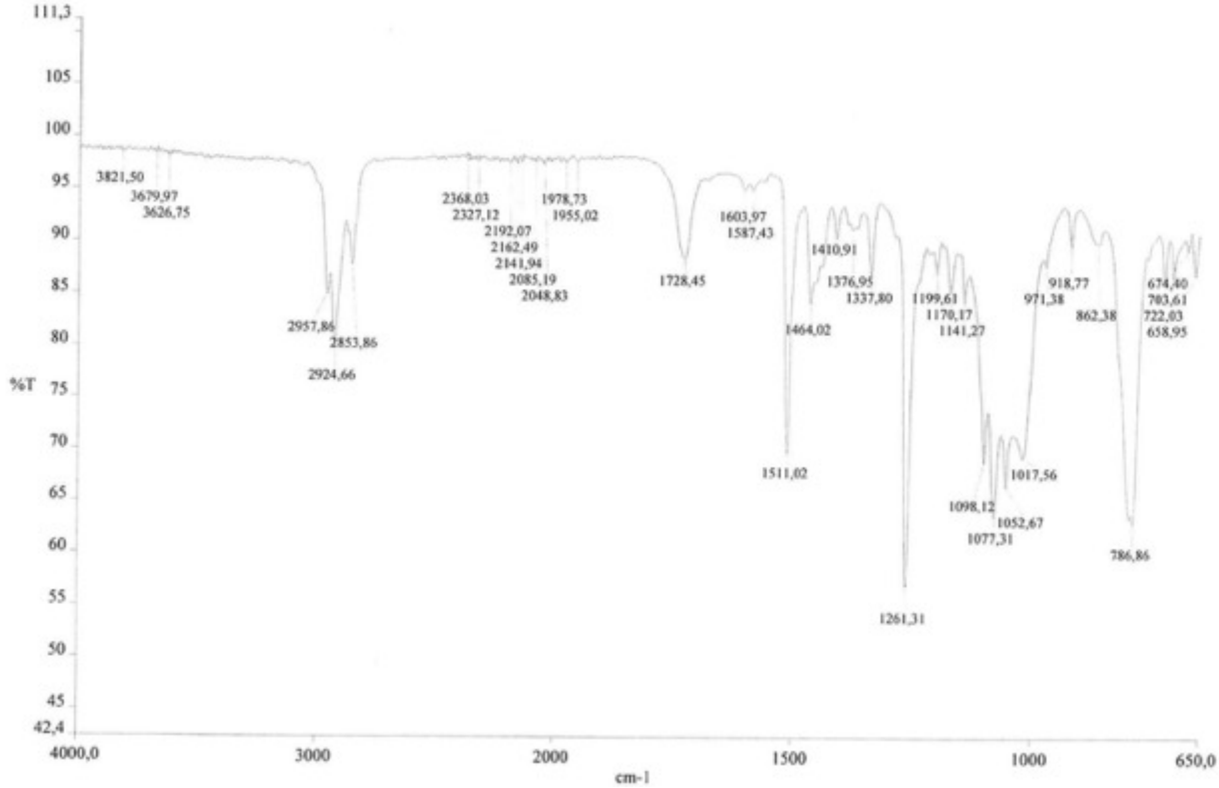
%T
cm-1
111,3
105
100
95
90
85
80
75
70
65
60
55
50
45
42,4
4000,0
3000
2000
1500
1000
650,0
3821,50
3679,97
3626,75
2957,86
2924,66
2853,86
2368,03
2327,12
2192,07
2162,49
2141,94
2085,19
2048,83
1978,73
1955,02
1728,45
1603,97
1587,43
1511,02
1464,02
1410,91
1376,95
1337,80
1261,31
1199,61
1170,17
1141,27
1098,12
1077,31
1052,67
1017,56
971,38
918,77
862,38
786,86
674,40
703,61
722,03
658,95

(4,7-dimethoxybenzofuran-3- yl)methanol **(26)**

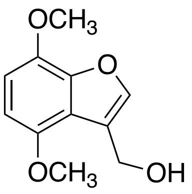

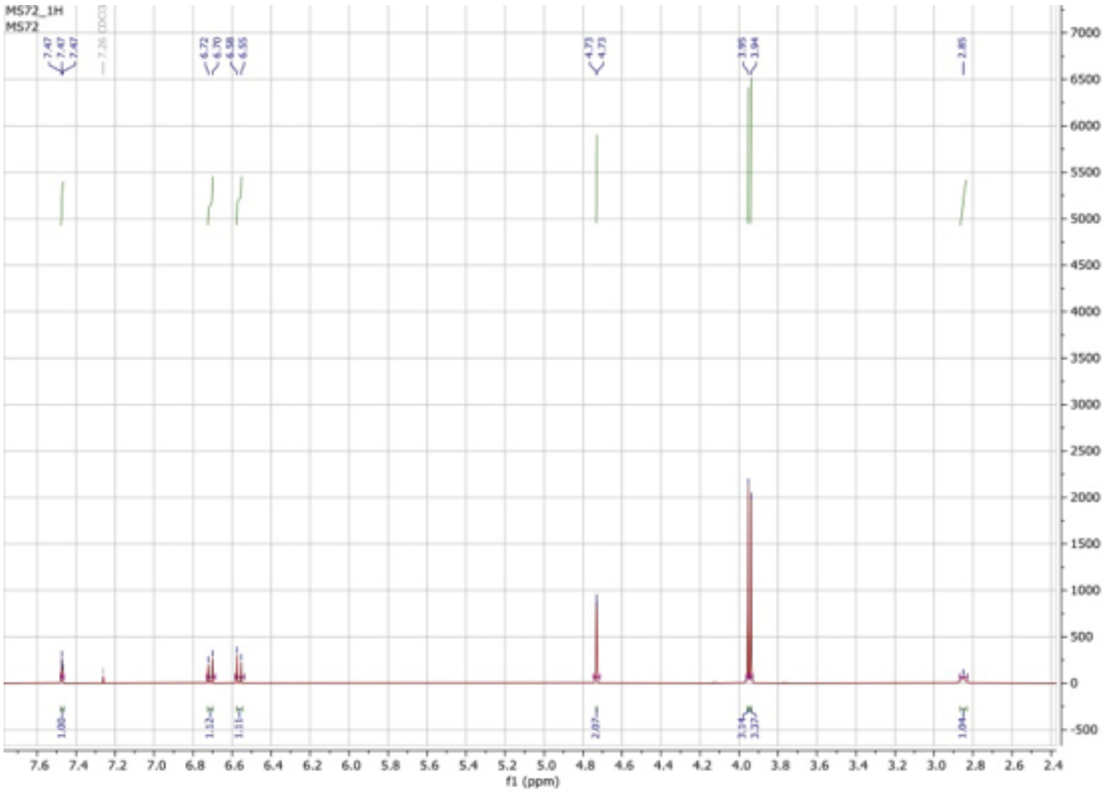

^{1}H NMR (400 MHz, Chloroform-d) δ 7.47 (d, *J* = 1.0 Hz, 1H), 6.71 (d, *J* = 8.6 Hz, 1H), 6.56 (d, *J* = 8.6 Hz, 1H), 4.73 (d, *J* = 0.9 Hz, 2H), 3.95 (s, 3H), 3.94 (s, 3H), 2.85 (s, 1H).

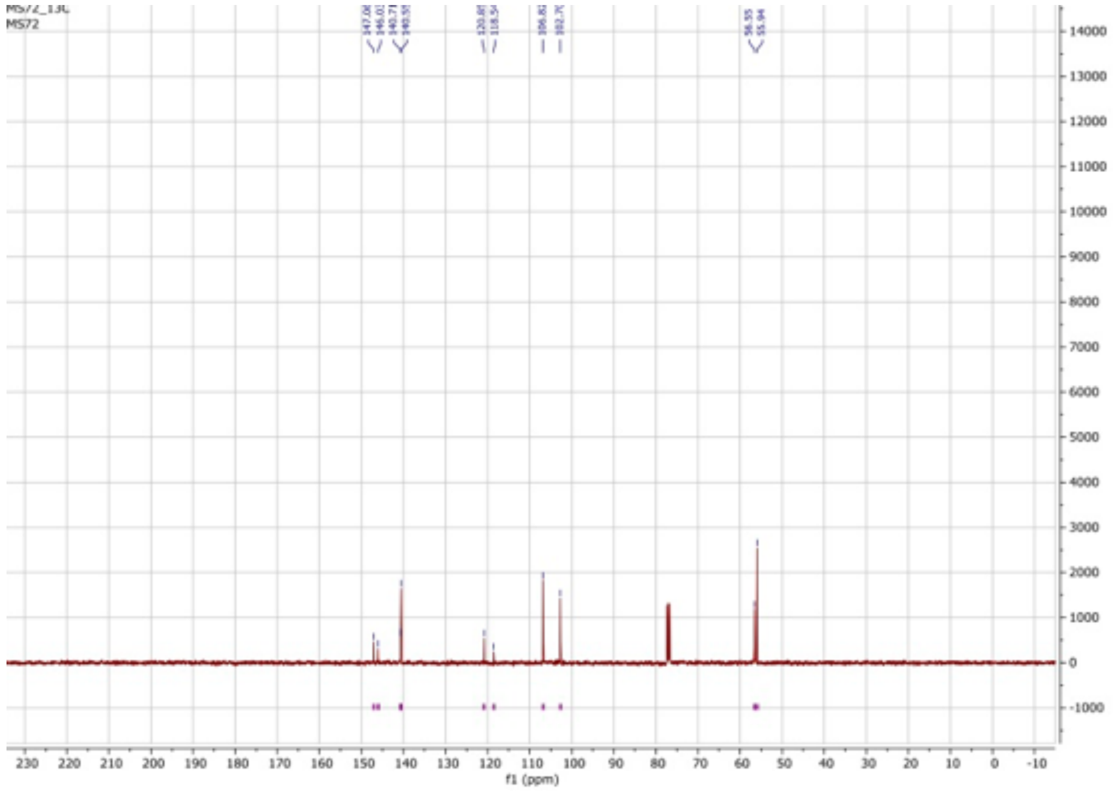

^{13}C NMR (101 MHz, Chloroform-d) δ 147.06, 146.03, 140.71, 140.55, 120.85, 118.54, 106.82, 102.70, 56.55, 55.94.

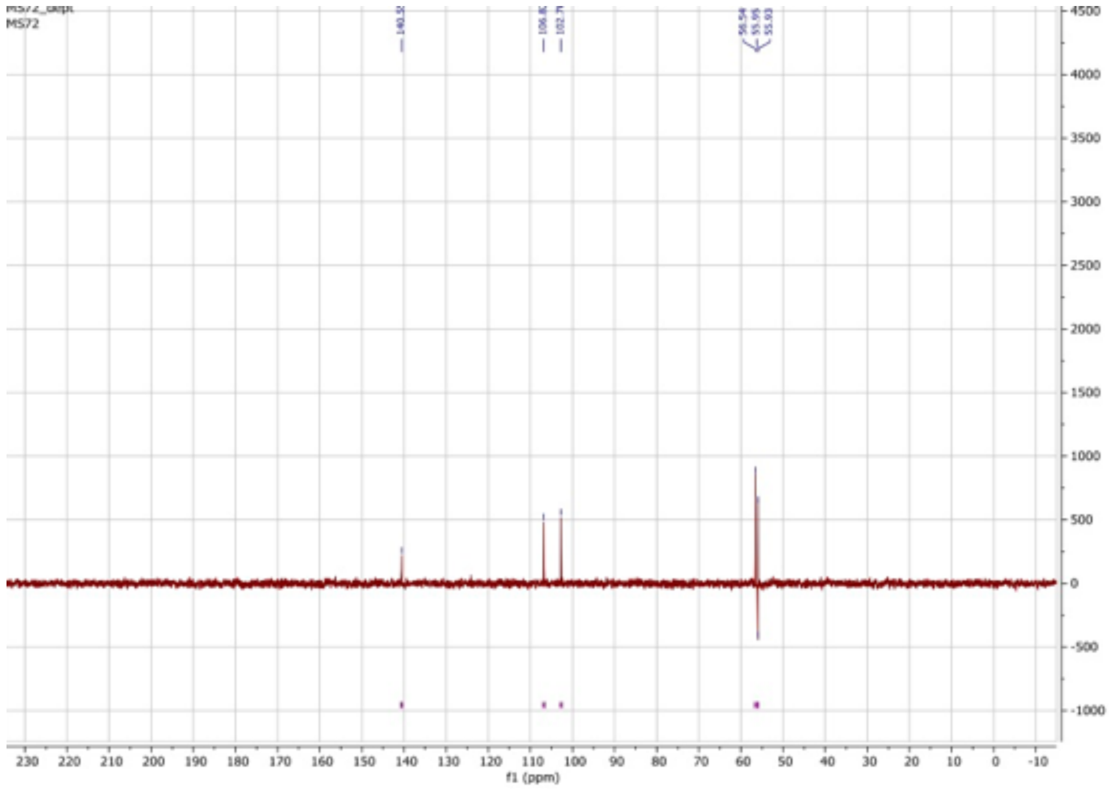

^{13}C NMR (101 MHz, Chloroform-d) δ 140.55, 106.82, 102.70, 56.54, 55.95, 55.93.

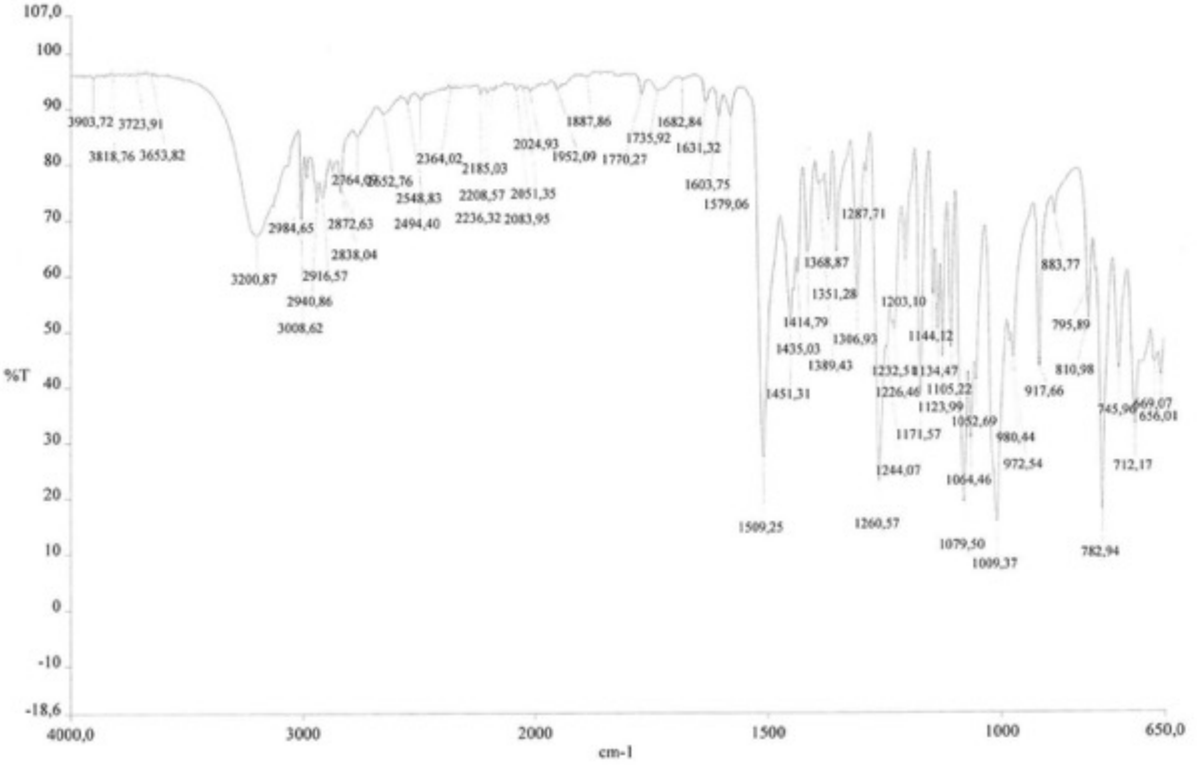
%T
cm-1
4000,0
3000
2000
1500
1000
650,0
107,0
-18,6
3200,87
1509,25
1260,57
1079,50
1009,37
782,94

4,7-dimethoxybenzofuran-3-carbaldehyde (**27**)

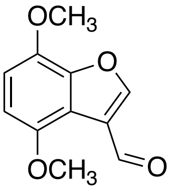

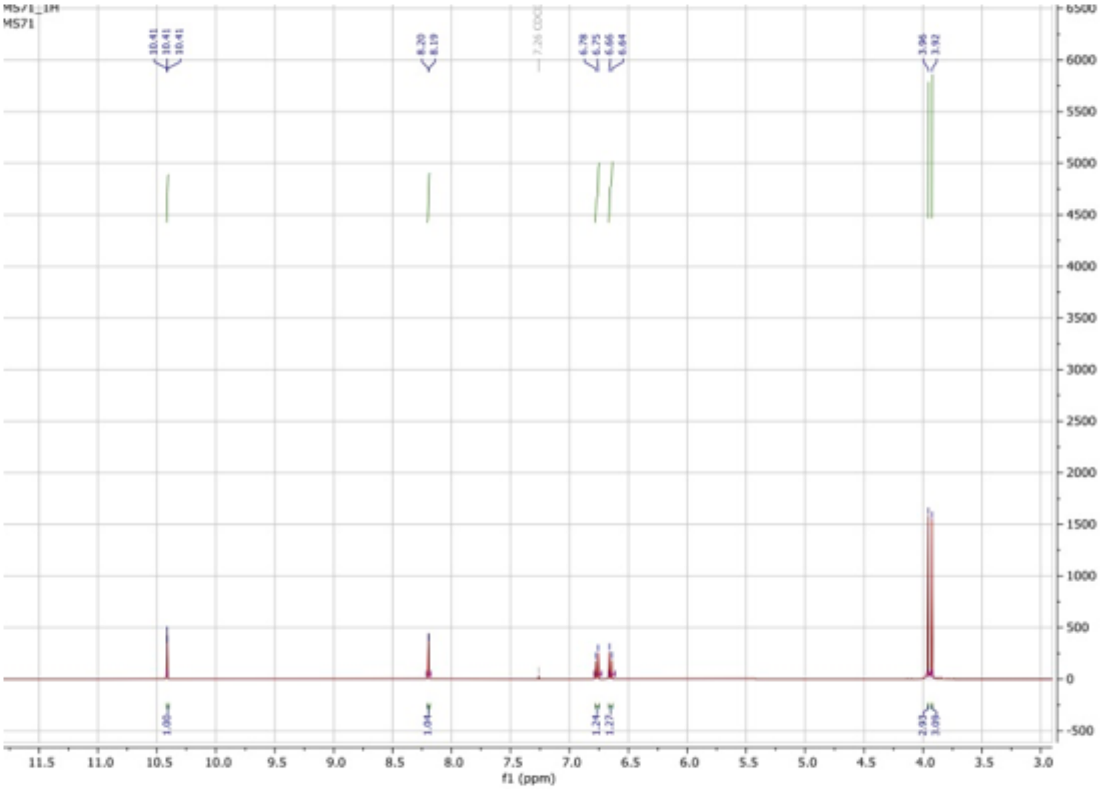

^{1}H NMR (400 MHz, Chloroform-d) δ 10.41 – 10.41 (m, 1H), 8.21 – 8.18 (m, 1H), 6.77 (d, *J* = 8.6 Hz, 1H), 6.65 (d, *J* = 8.6 Hz, 1H), 3.96 (s, 3H), 3.92 (s, 3H).

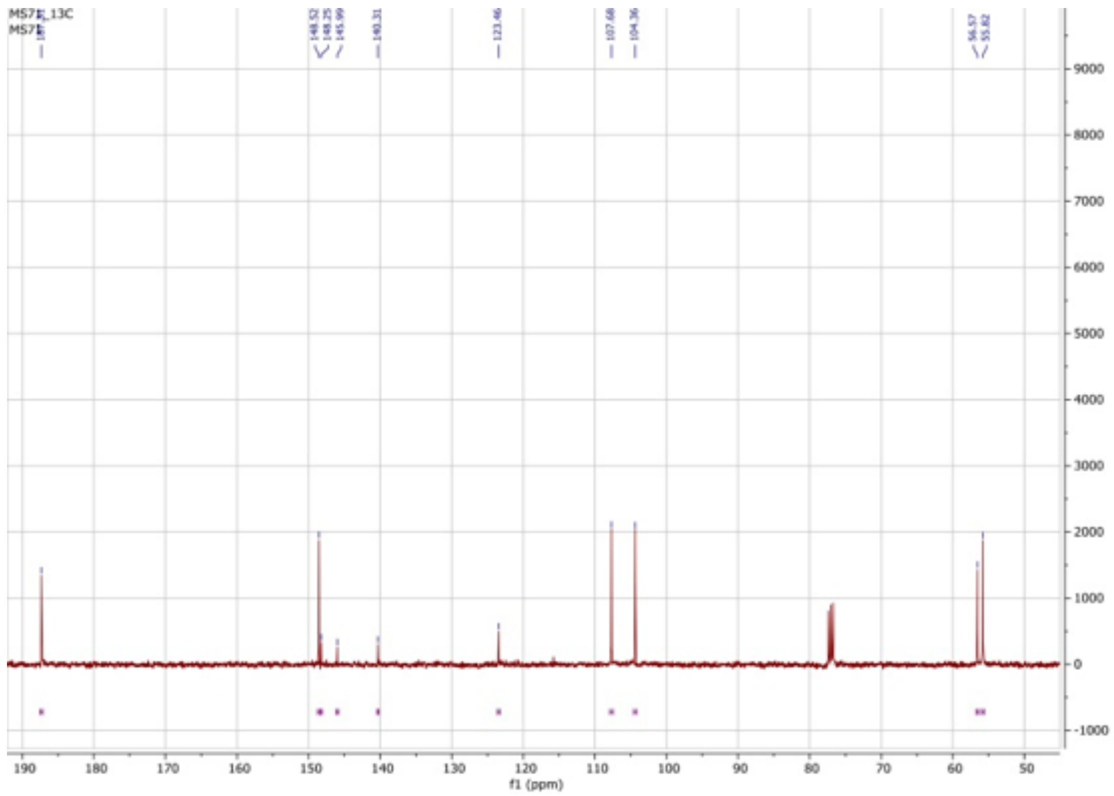

^{13}C NMR (101 MHz, Chloroform-d) δ 187.31, 148.52, 148.25, 145.99, 140.31, 123.46, 107.68, 104.36, 56.57, 55.82.

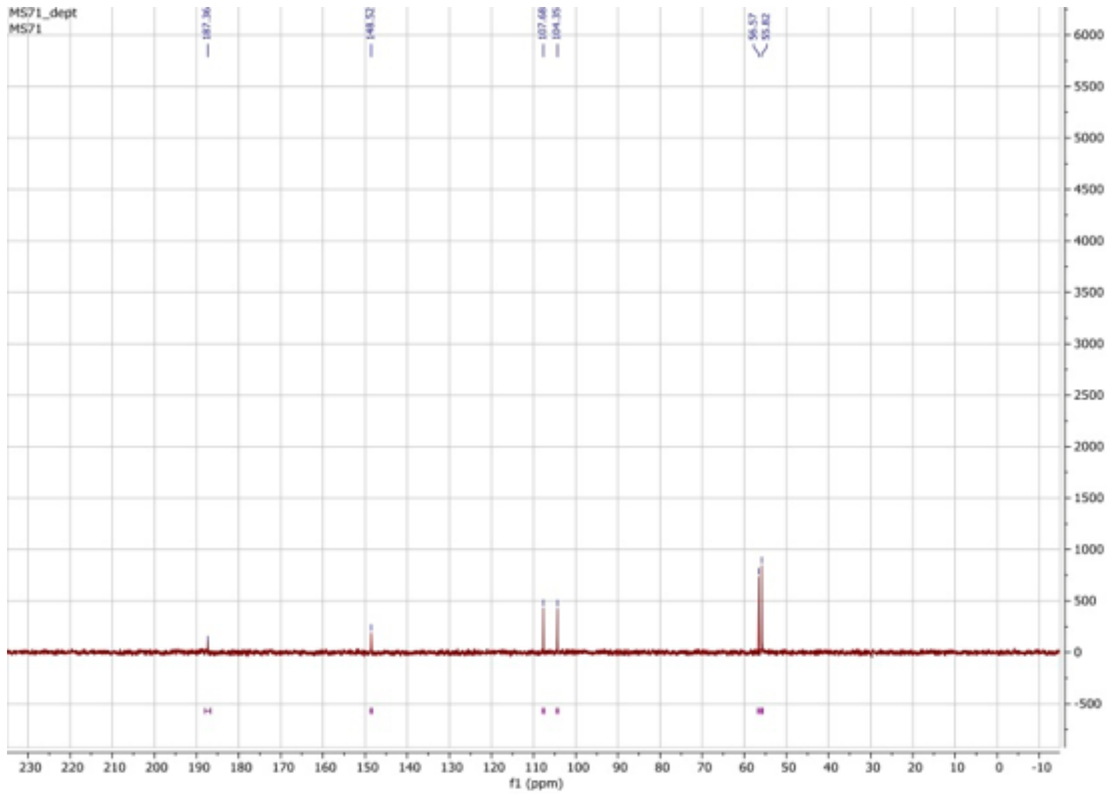

^{13}C NMR (101 MHz, Chloroform-d) δ 187.36, 148.52, 107.68, 104.35, 56.57, 55.82.

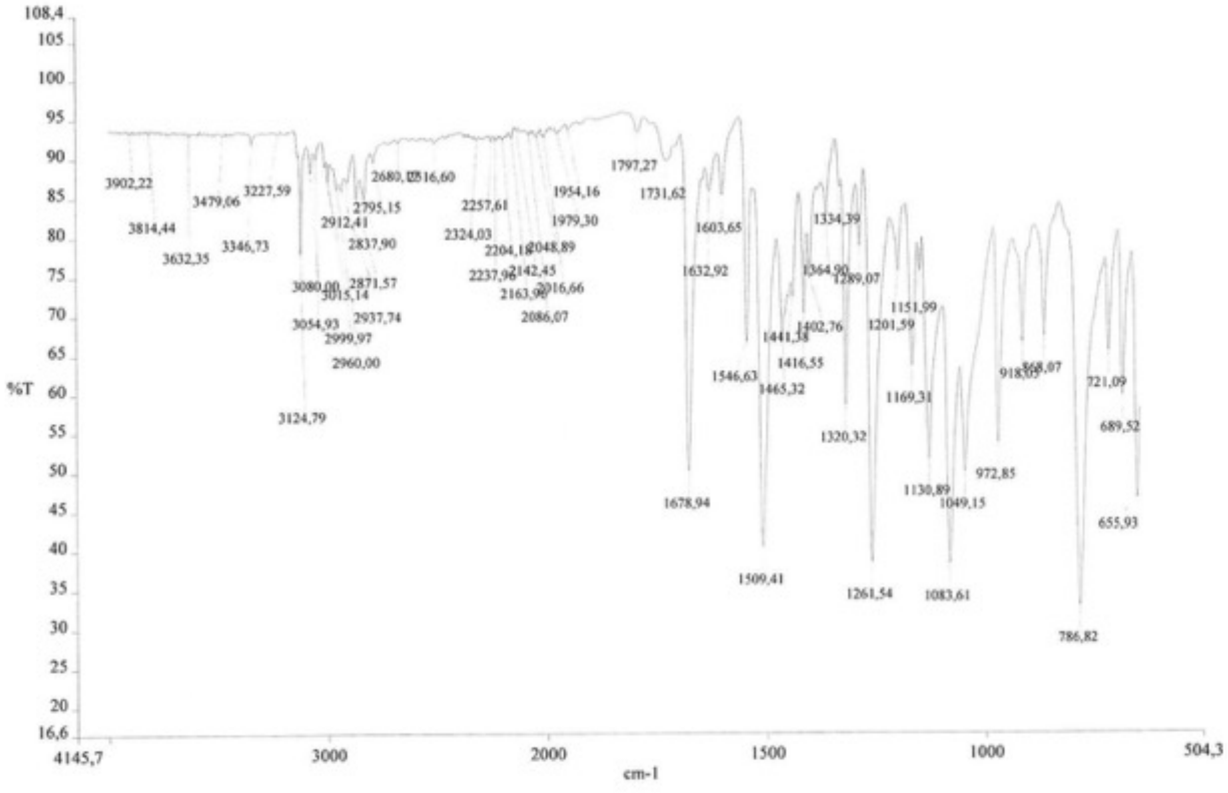
%T
cm-1
4145,7
504,3
1678,94
1509,41
1261,54
1083,61
786,82
3124,79

(E)-3-(4,7-dimethoxybenzofuran-6-yl)-1-phenylprop-2-en-1-one **(23)**

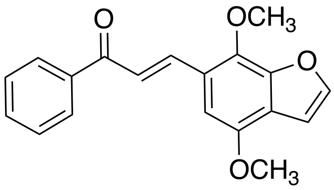

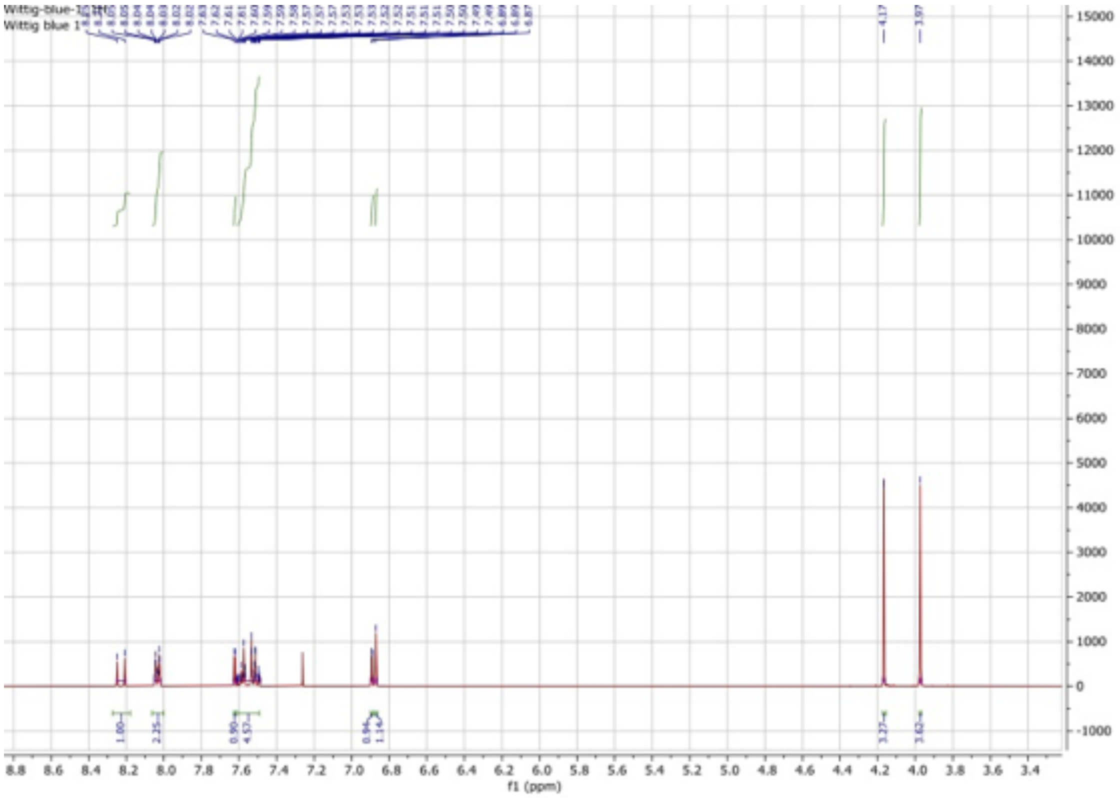

^{1}H NMR (400 MHz, Chloroform-*d*) δ 8.23 (d, J = 15.9 Hz, 1H), 8.05 – 8.02 (m, 2H), 7.62 (d, J = 2.1 Hz, 1H), 7.61 – 7.49 (m, 4H), 6.89 (d, J = 2.1 Hz, 1H), 6.87 (s, 1H), 4.17 (s, 3H), 3.97 (s, 3H).

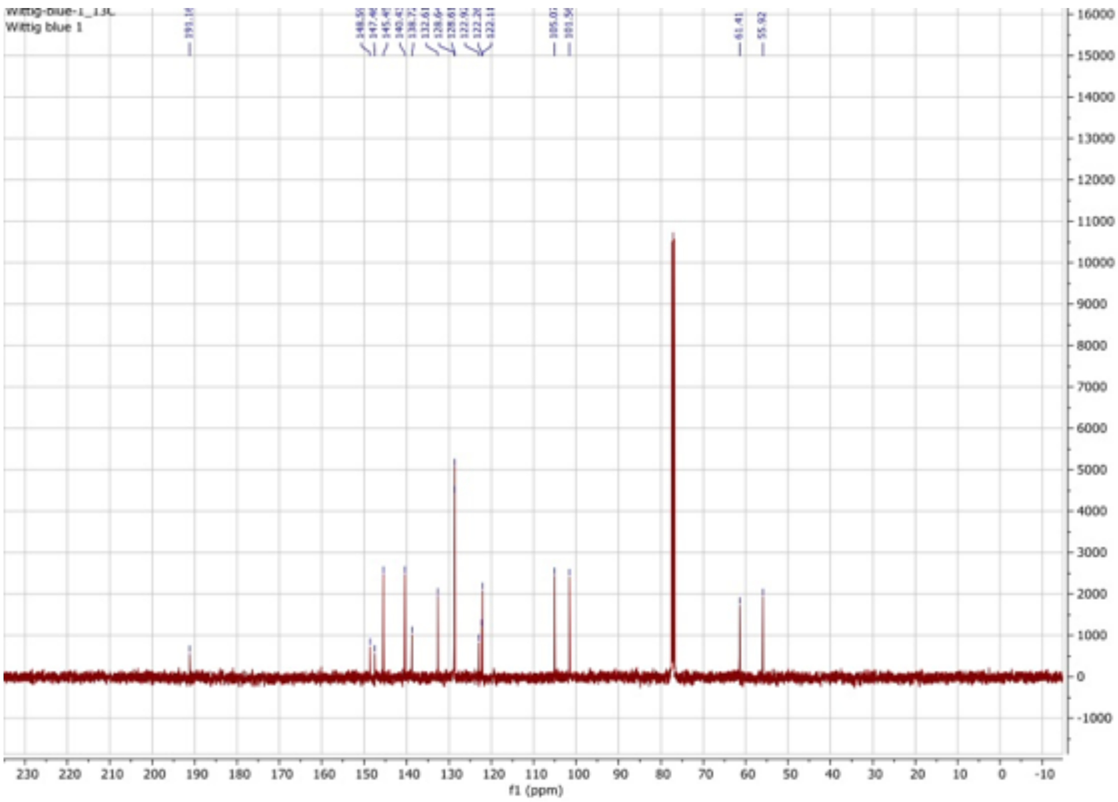

^{13}C NMR (101 MHz, Chloroform-d) δ 191.16, 148.59, 147.46, 145.45, 140.43, 138.72, 132.61, 128.64, 128.61, 122.92, 122.26, 122.11, 105.07, 101.56, 61.41, 55.92.

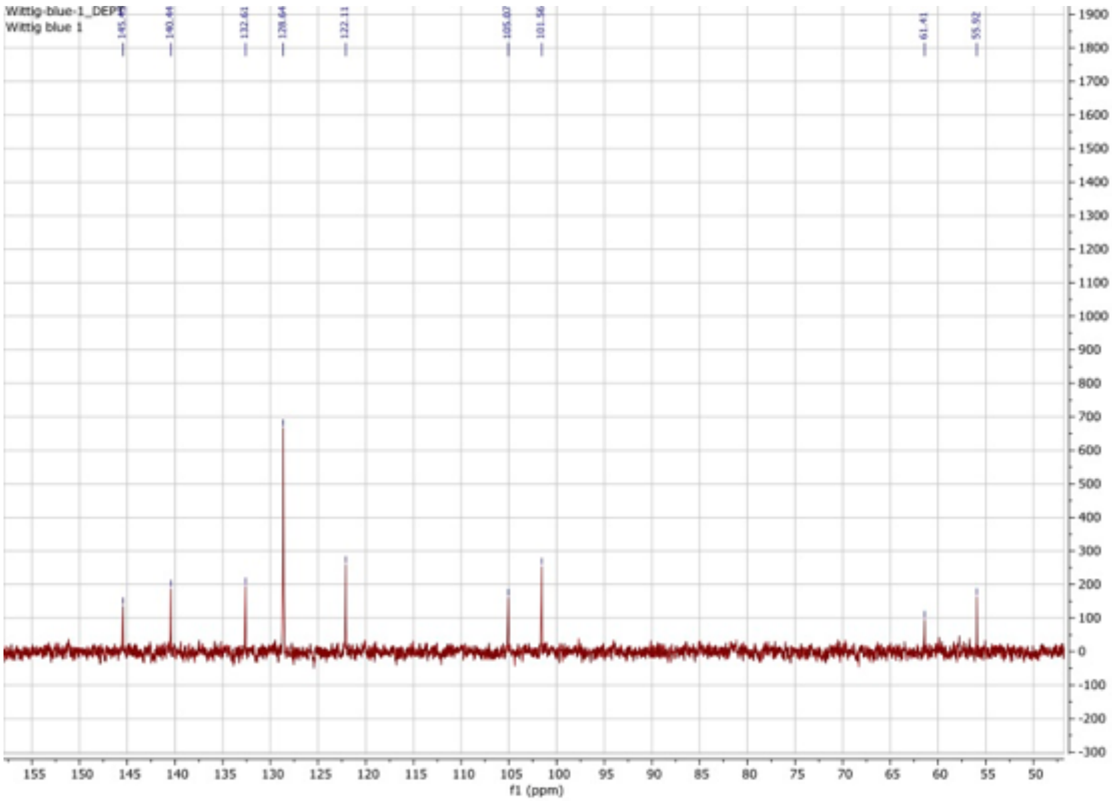

^{13}C NMR (101 MHz, Chloroform-d) δ 145.45, 140.44, 132.61, 128.64, 122.11, 105.07, 101.56, 61.41, 55.92.

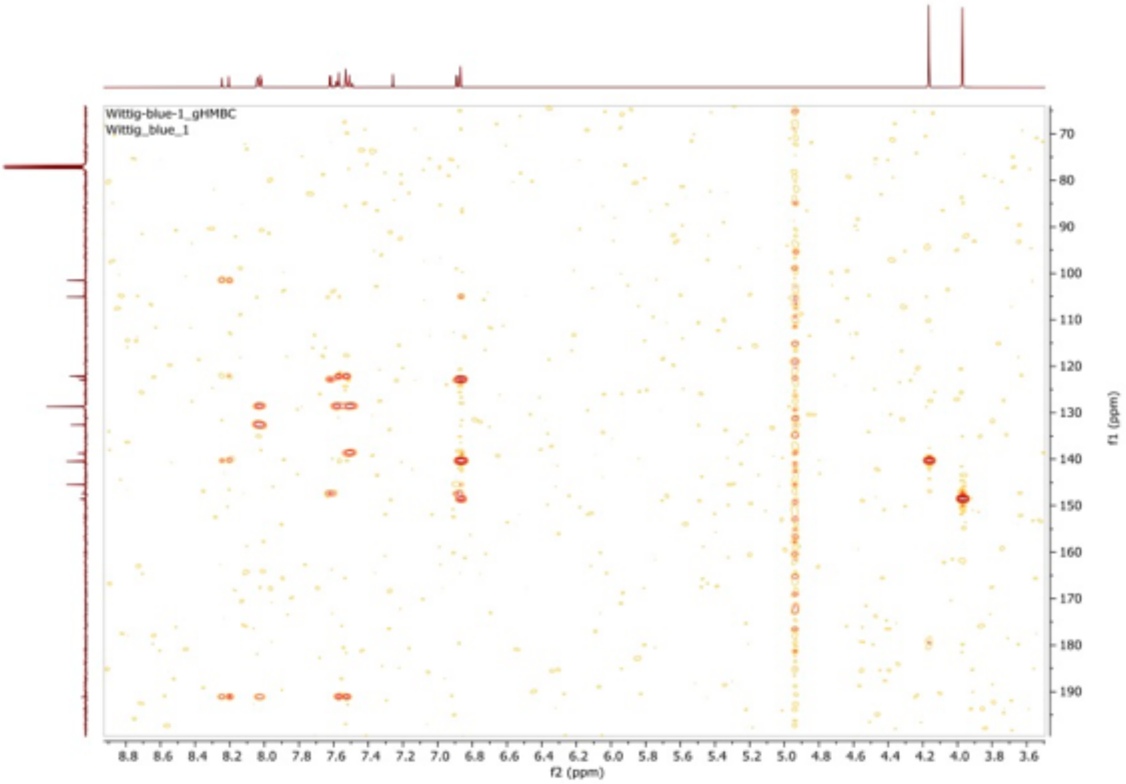
Wittig-blue-1_gHMBC
Wittig_blue_1
f2 (ppm)
f1 (ppm)

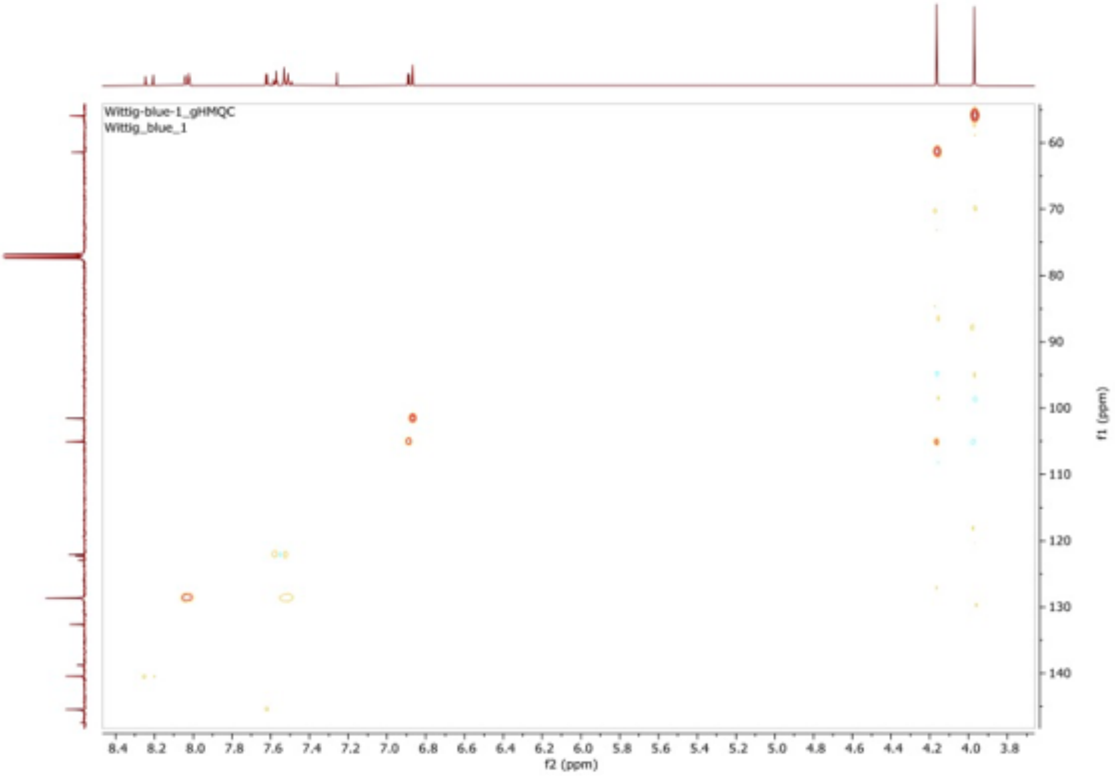
Wittig-blue-1_gHMQC
Wittig_blue_1
f2 (ppm)
f1 (ppm)

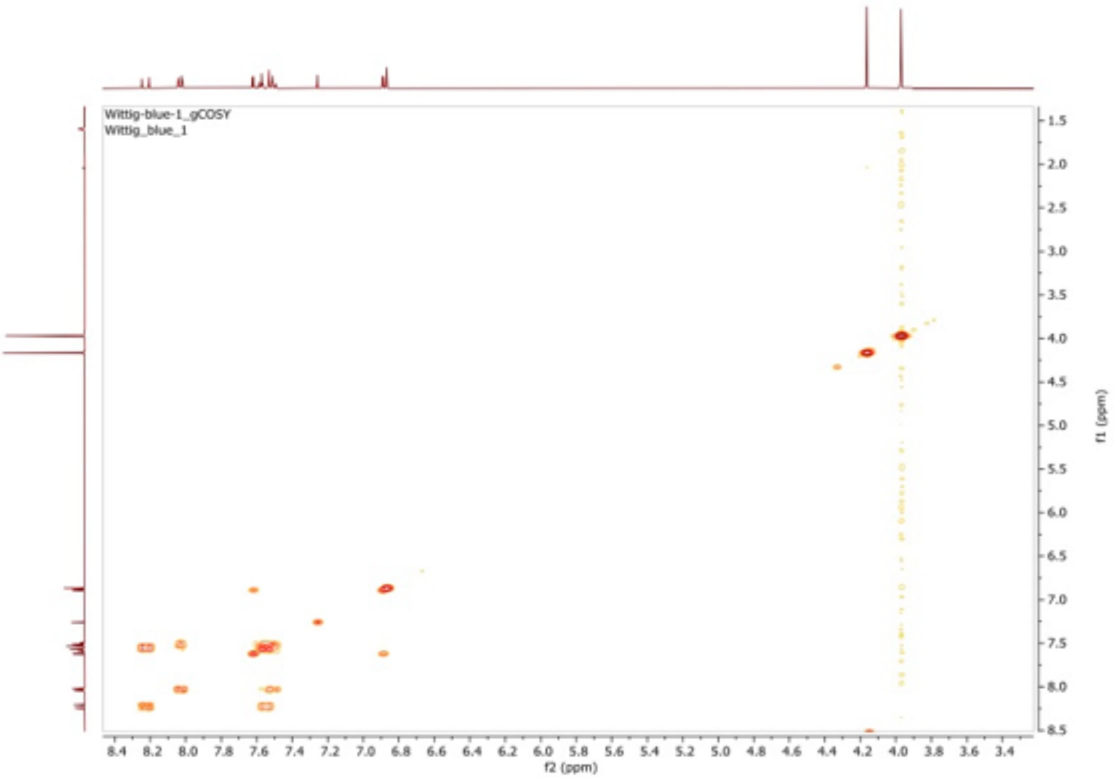
Wittig-blue-1_gCOSY
Wittig_blue_1
f2 (ppm)
f1 (ppm)

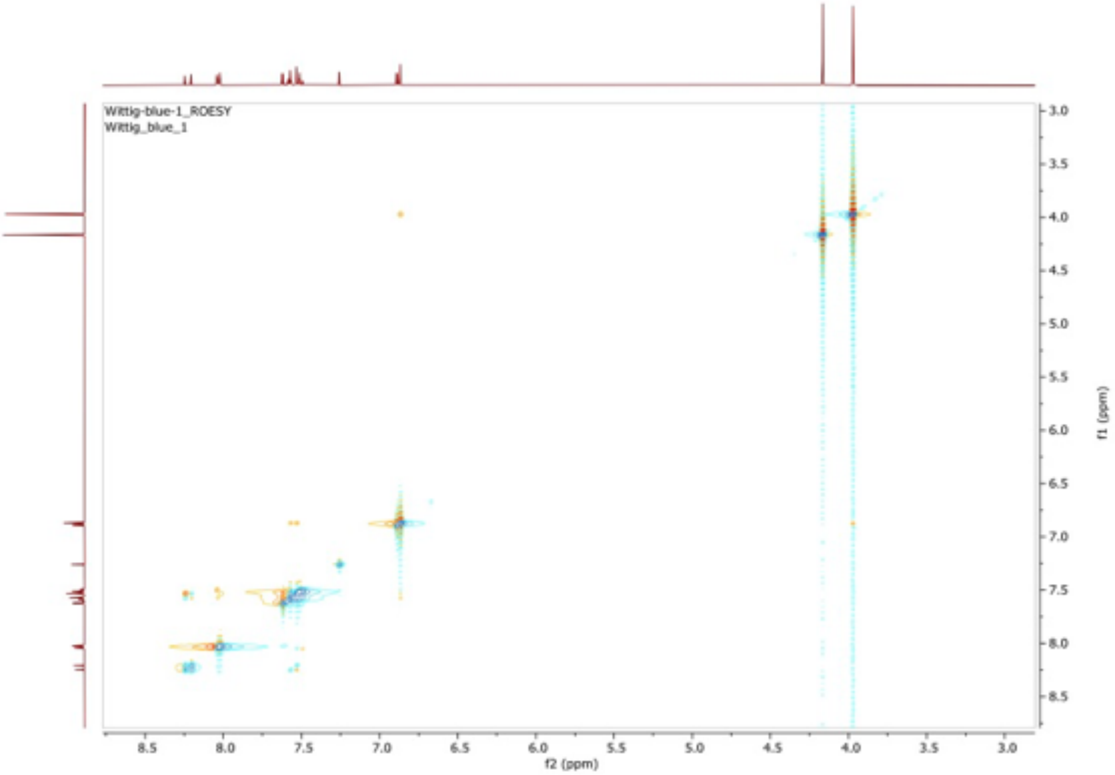
Wittig-blue-1_ROESY
Wittig_blue_1
f2 (ppm)
f1 (ppm)

Area % Report

Data File: C:\32Karat\Projects\Default\Data\delia\Martina\vell T 050521.dat
Method: C:\32Karat\Projects\Default\Method\standard 0.7 ml.met
Acquired: 05/05/2021 15.47.23
Printed: 04/08/2022 13.20.32

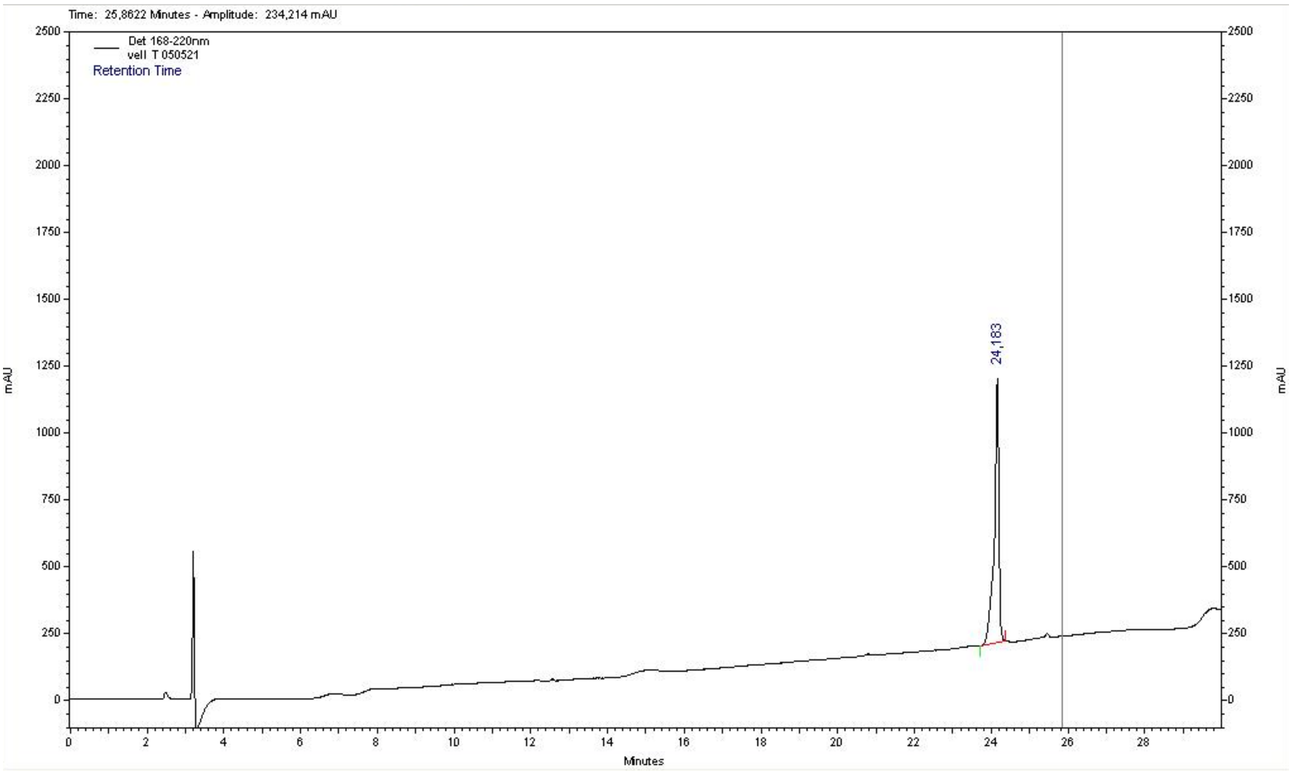

Det 168-220nm
Results

Time	Area	Area %	Height	Height %
24,183	8149082	100,00	987191	100,00

Totals				
	8149082	100,00	987191	100,00

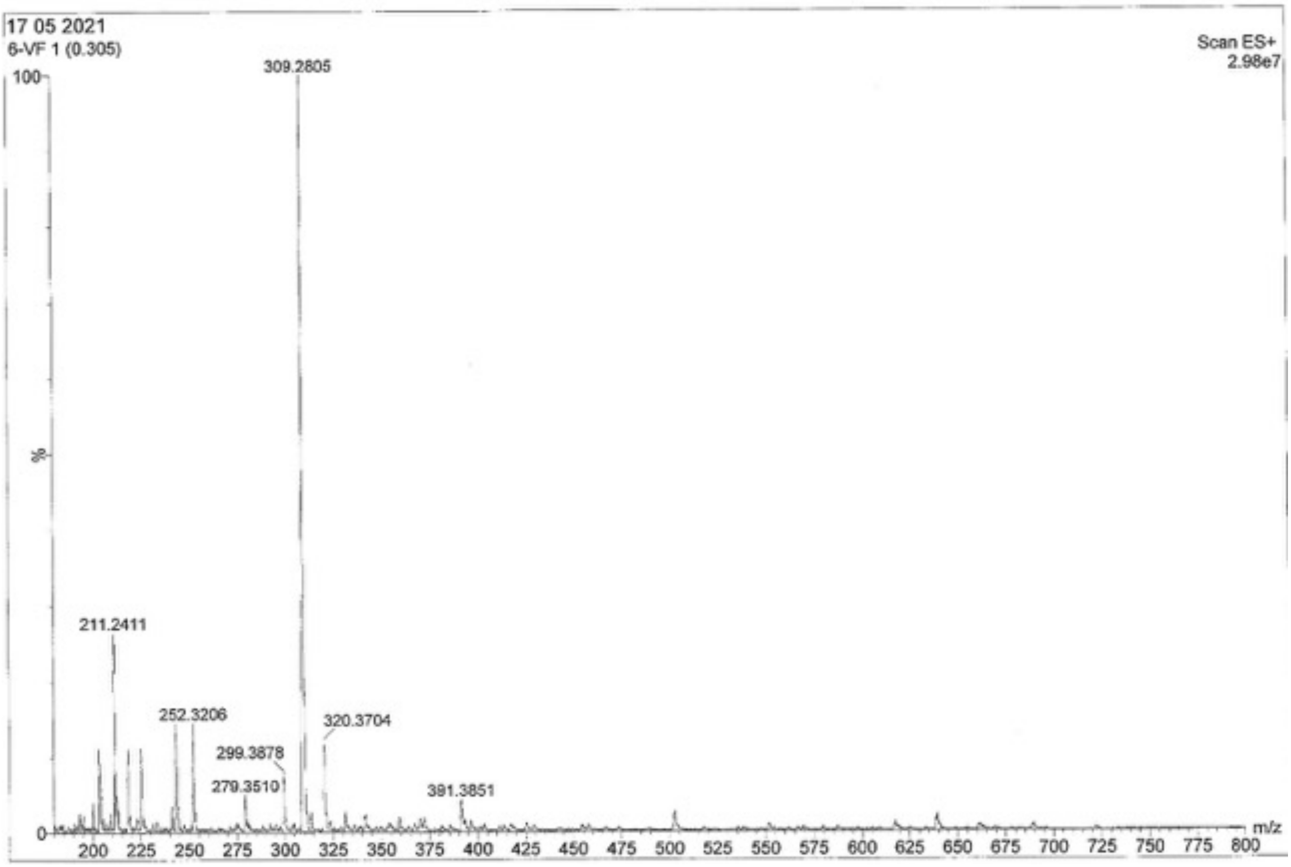

17 05 2021
6-VF 1 (0.305)
Scan ES+
2.98e7
309.2805
211.2411
252.3206
320.3704
299.3878
279.3510
391.3851
%
m/z

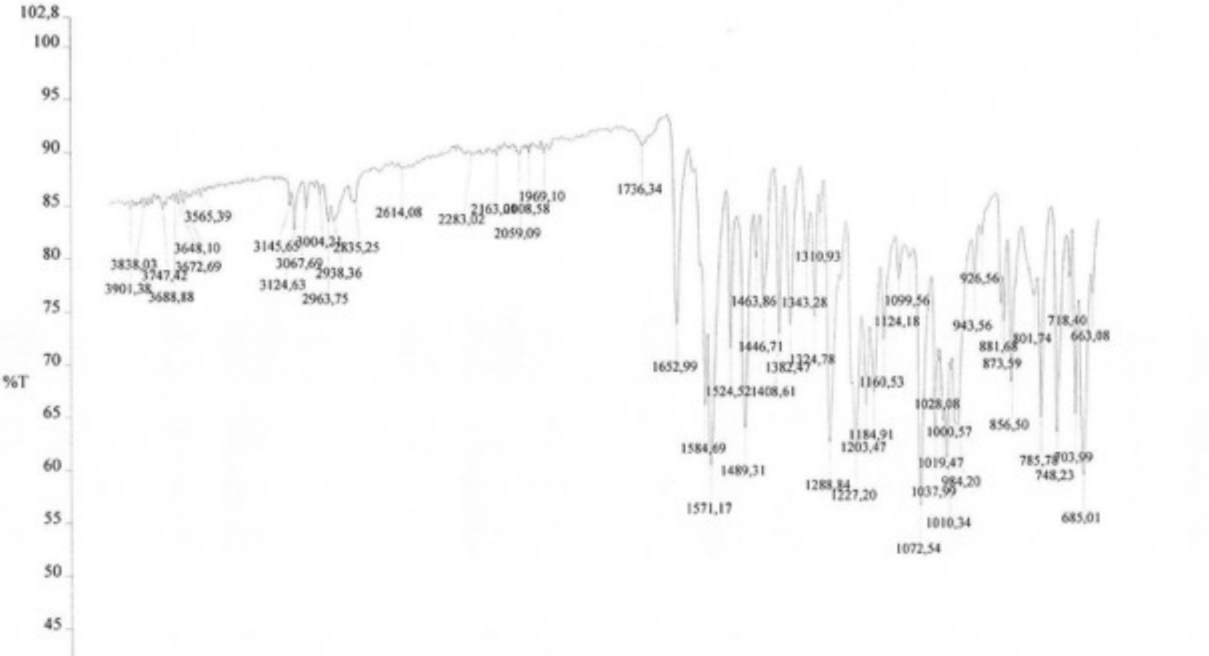

%T
cm-1
1736,34
1652,99
1571,17
1584,69
1489,31
1072,54
1010,34
685,01

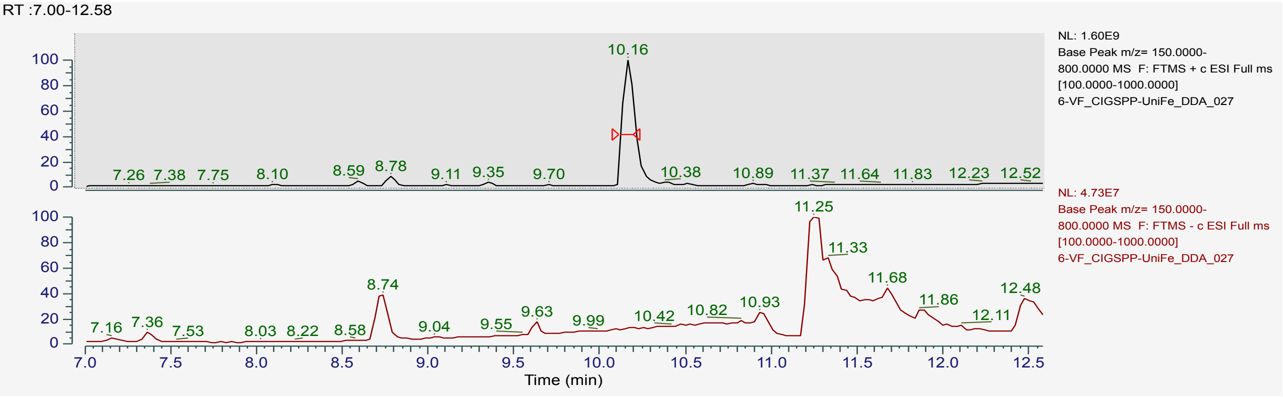

6-VF_CIGSPP-UniFe_DDA_027 #2177-2198 RT: 10.11-10.2 AV: 4 SB: 7 9.85-10.03 NL: 9.90E+008
T: FTMS + c ESI Full ms [100.0000-1000.0000]

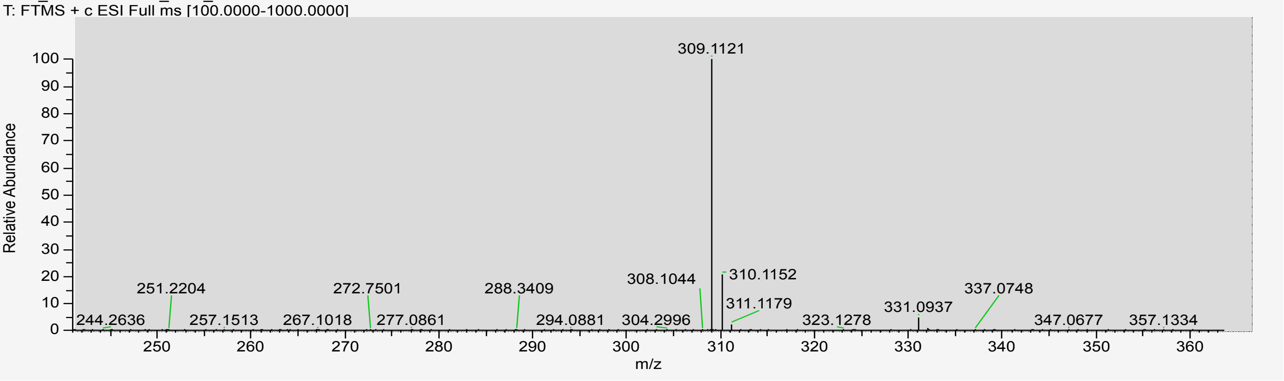

Peak Mass	Display Formula	Combined Fit	RDB	Delta [ppm]	Theo. mass	Rank	Combined Score	# Matched Iso.	# Missed Iso.	MS Cov. [%]	Pattern Cov. [%]
309,1121	$C_{19}H_{17}O_4$	18,7690501150057	11,5	-0,21	309,11214	1	95,49	4	4	99,75	98,82
331,0937	$C_{19}H_{16}O_4{}^{23}Na$	12,9725080889243	11,5	-1,02	331,09408	7	93,67	2	6	98,15	97,22

6-VF_CIGSPP-UniFe_DDA_027 #2184 RT: 10.14 AV: 1 NL: 1.30E+008
T: FTMS + c ESI d Full ms2 309.1120@hcd36.00 [50.0000-335.0000]

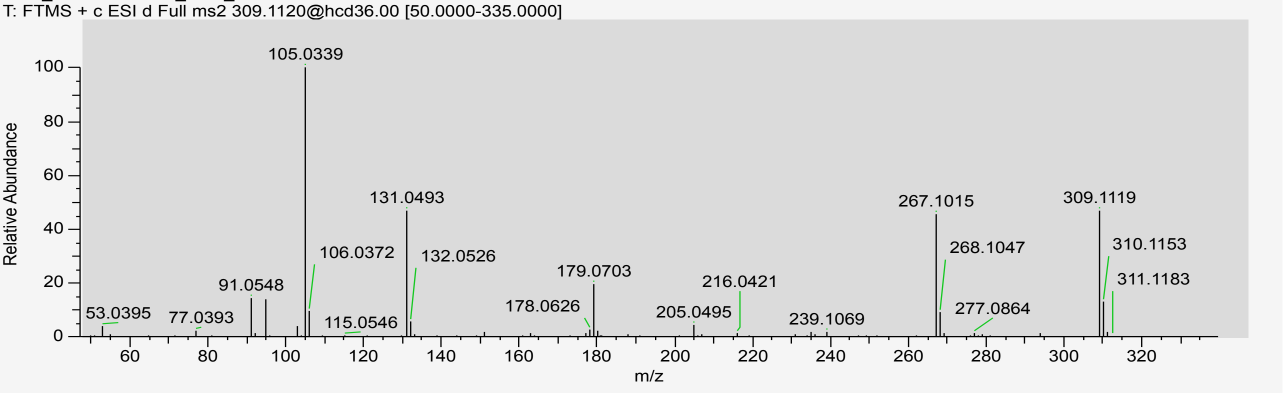

(E)-3-(4,7-dimethoxybenzofuran-2-yl)-1-phenylprop-2-en-1-one **(22)**

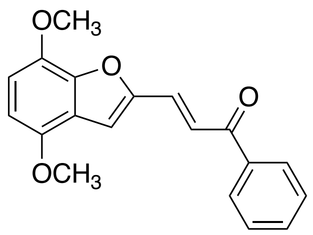

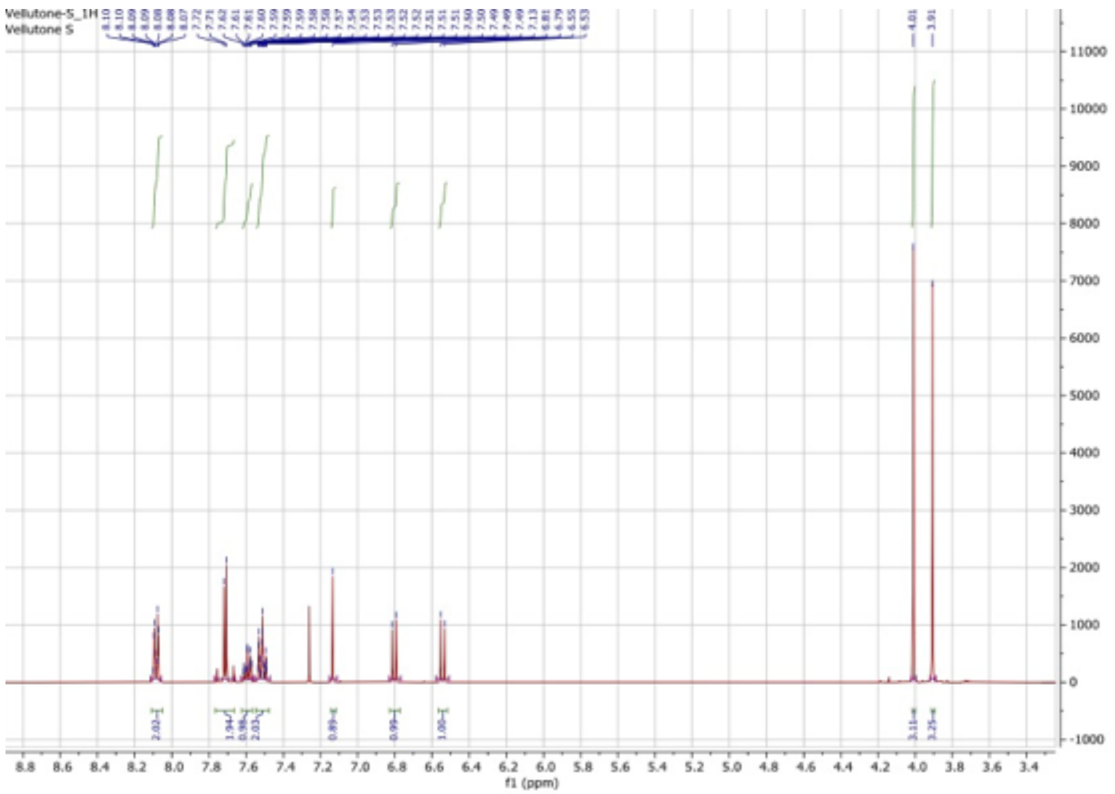

^{1}H NMR (400 MHz, Chloroform-*d*) δ 8.12 – 8.06 (m, 2H), 7.71 (d, *J* = 4.9 Hz, 2H), 7.59 (ddt, *J* = 8.3, 6.6, 1.4 Hz, 1H), 7.55 – 7.47 (m, 2H), 7.13 (s, 1H), 6.80 (d, *J* = 8.6 Hz, 1H), 6.54 (d, *J* = 8.6 Hz, 1H), 4.01 (s, 3H), 3.91 (s, 3H).

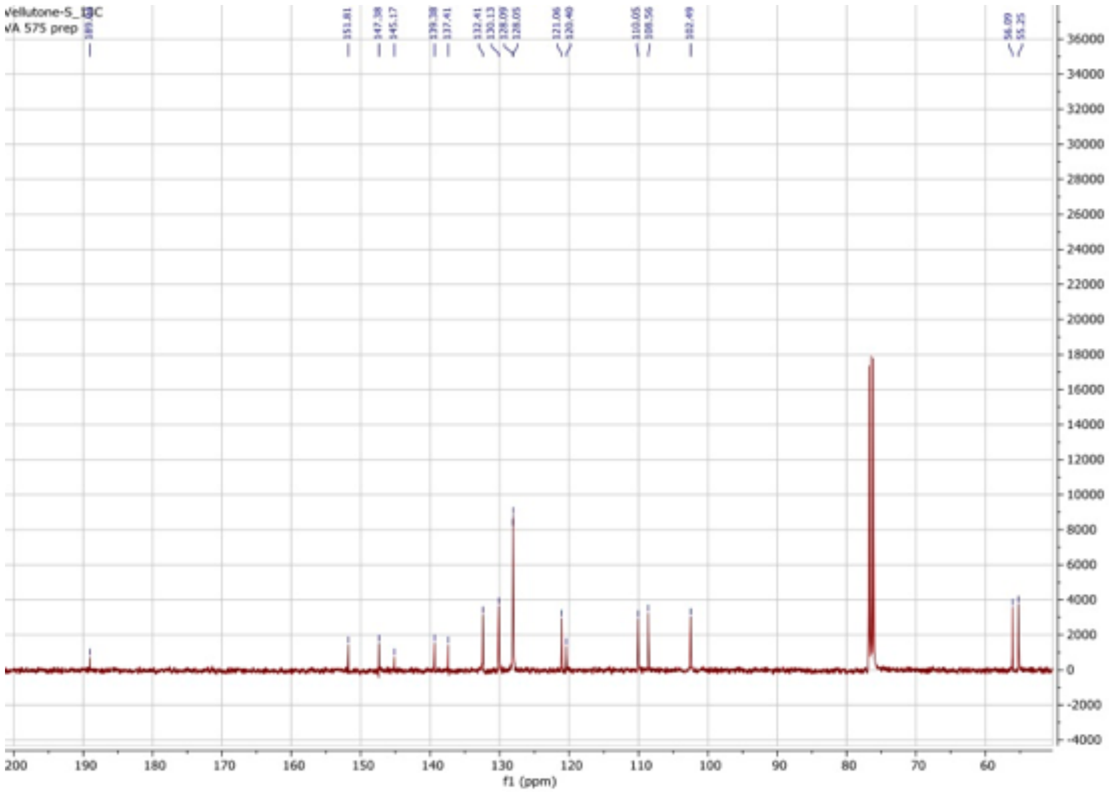

^{13}C NMR (101 MHz, Chloroform-d) δ 189.04, 151.81, 147.38, 145.17, 139.38, 137.41, 132.41, 130.13, 128.09, 128.05, 121.06, 120.40, 110.05, 108.56, 102.49, 56.09, 55.25.

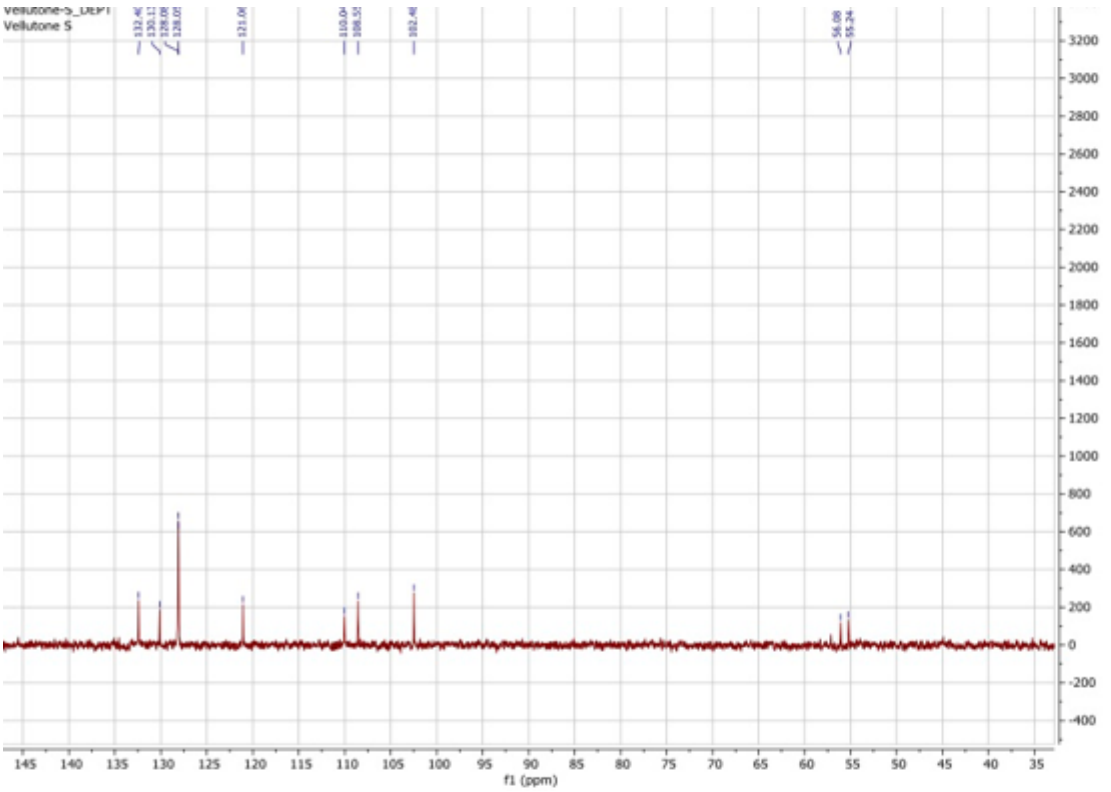

^{13}C NMR (101 MHz, Chloroform-d) δ 132.40, 130.13, 128.08, 128.05, 121.06, 110.04, 108.55, 102.48, 56.08, 55.24.

Area % Report

Data File: C:\32Karat\Projects\Default\Data\delia\Martina\vellutone S.dat
Method: C:\32Karat\Projects\Default\Method\standard 0.7 ml.met
Acquired: 15/04/2021 15.58.12
Printed: 04/08/2022 13.12.03

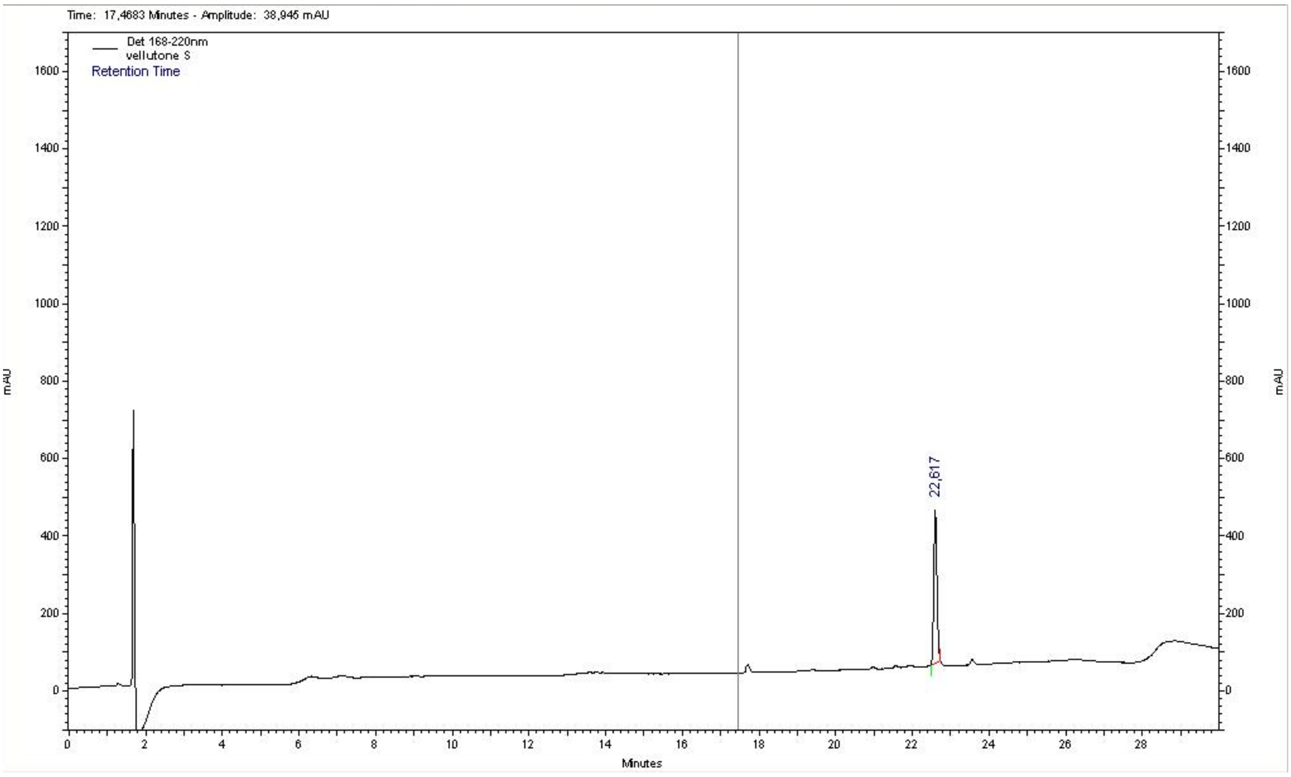

Det 168-220nm
Results

Time	Area	Area %	Height	Height %
22,617	2249606	100,00	392360	100,00

Totals				
	2249606	100,00	392360	100,00

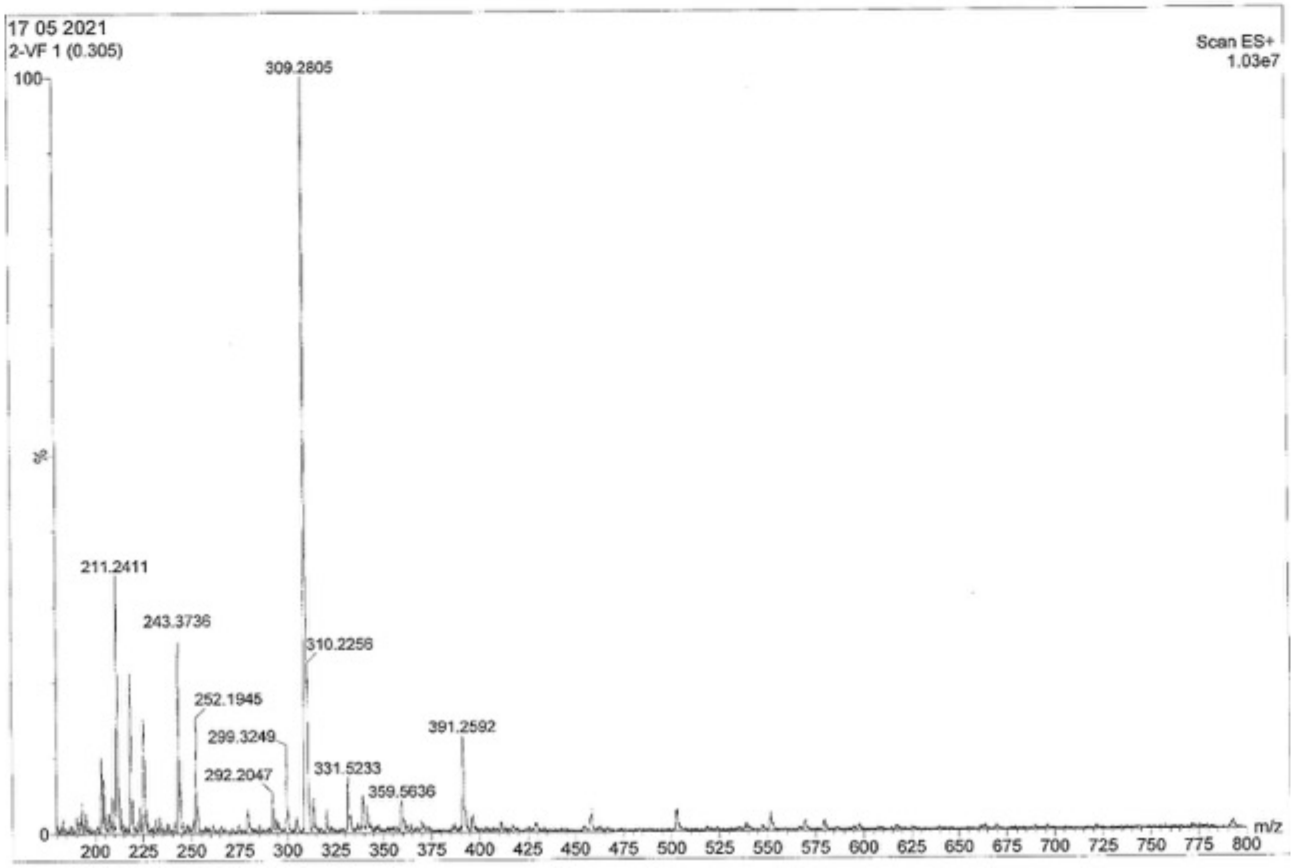
17 05 2021
2-VF 1 (0.305)
Scan ES+
1.03e7
309.2805
211.2411
243.3736
310.2256
252.1945
299.3249
391.2592
292.2047
331.5233
359.5636
%
m/z

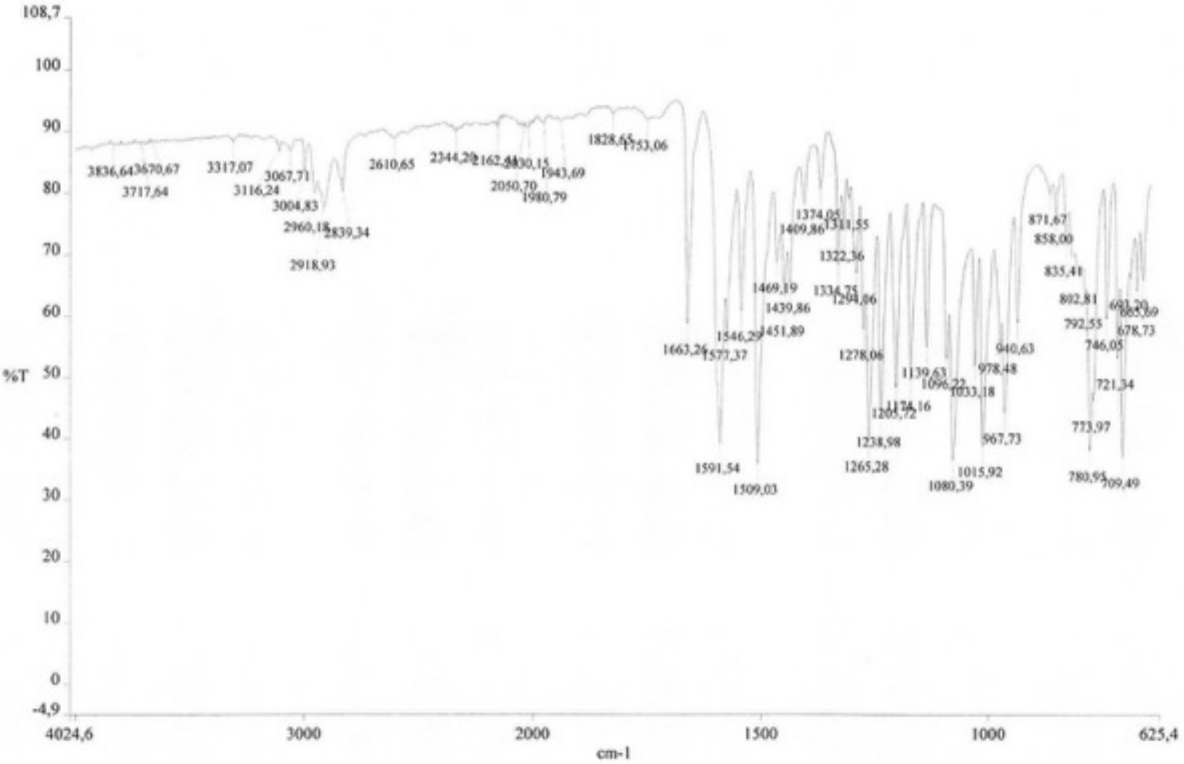
%T
cm-1
1591,54
1509,03
1663,26
1265,28
1080,39
1015,92
2918,93

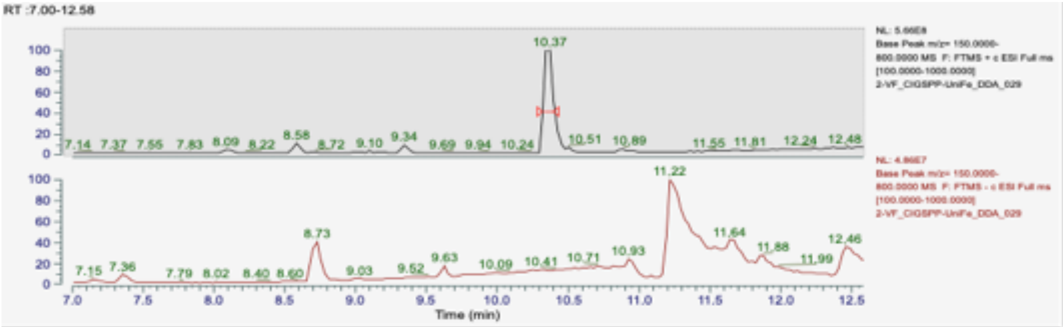

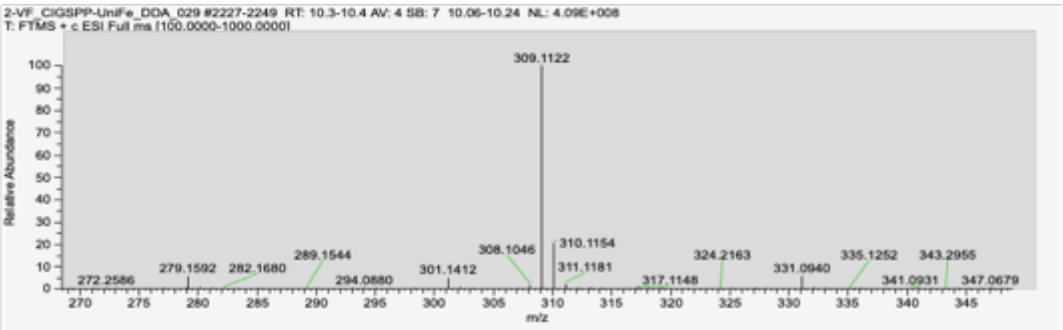

Peak Mass	Display Formula	Combined Fit	RDB	Delta [ppm]	Theo. mass	Rank	Combined Score	# Matched Iso.	# Missed Iso.	MS Cov. [%]	Pattern Cov. [%]
309,1122	$C_{19}H_{17}O_4$	25,1421183814927	11,5	0,19	309,11214	1	95,94	4	4	99,87	98,96
331,094	$C_{19}H_{16}O_4{}^{23}Na$	17,8402298795552	11,5	-0,37	331,09408	3	95,07	3	5	99,37	98,83

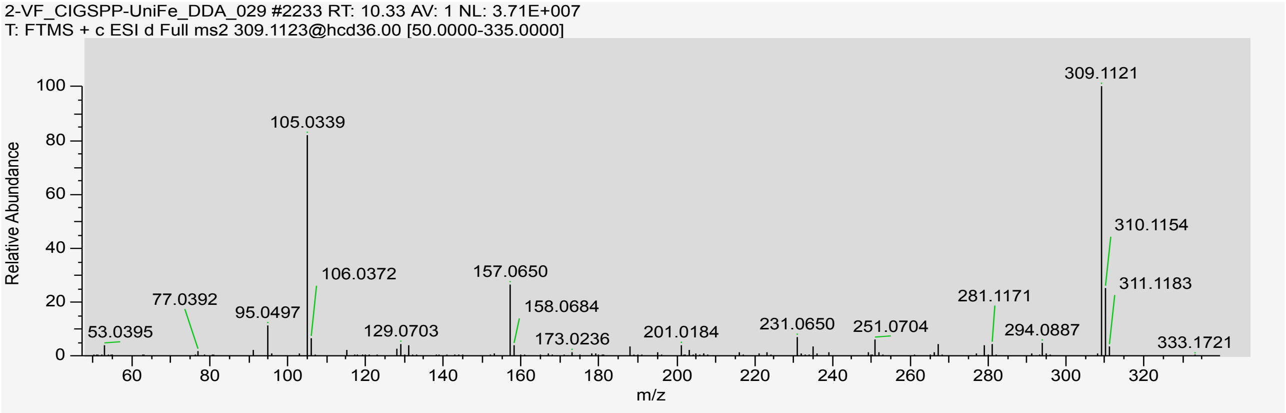

(*E*)-3-(4,7-dimethoxybenzofuran-3-yl)-1-phenylprop-2-en-1-one **(28)**

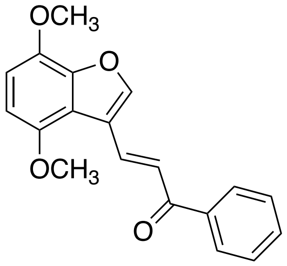

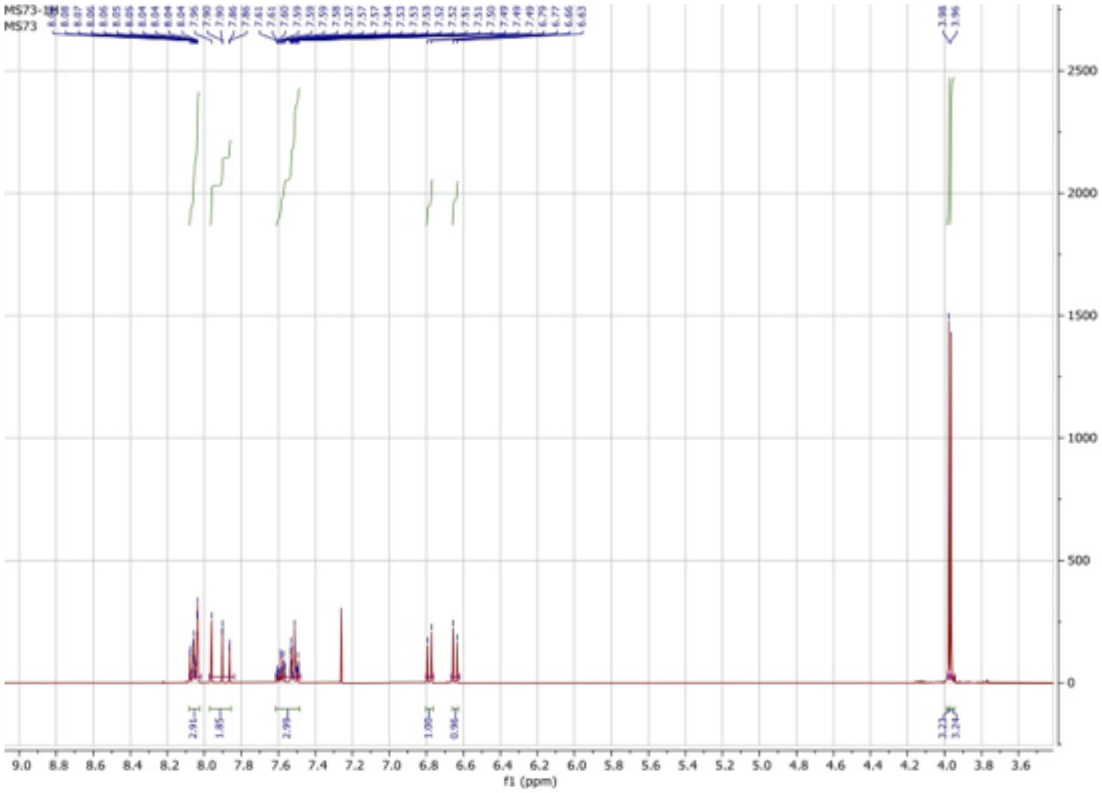

^{1}H NMR (400 MHz, Chloroform-*d*) δ 8.08 – 8.02 (m, 3H), 7.97 – 7.84 (m, 2H), 7.61 – 7.48 (m, 3H), 6.78 (d, *J* = 8.6 Hz, 1H), 6.65 (d, *J* = 8.7 Hz, 1H), 3.98 (s, 3H), 3.96 (s, 3H).

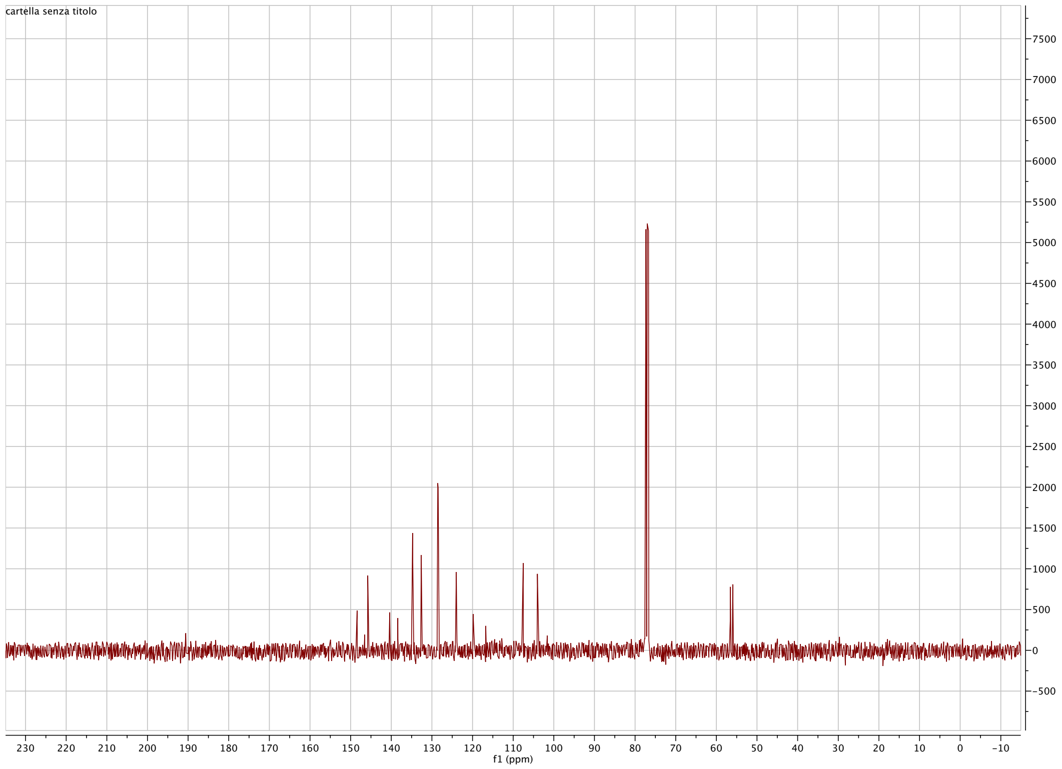

^{13}C NMR (101 MHz, Chloroform-d) δ 190.58, 148.41, 146.56, 145.78, 140.38, 138.38, 134.75, 132.62, 128.56, 128.46, 124.00, 119.85, 116.75, 107.53, 104.04, 56.56, 55.96.

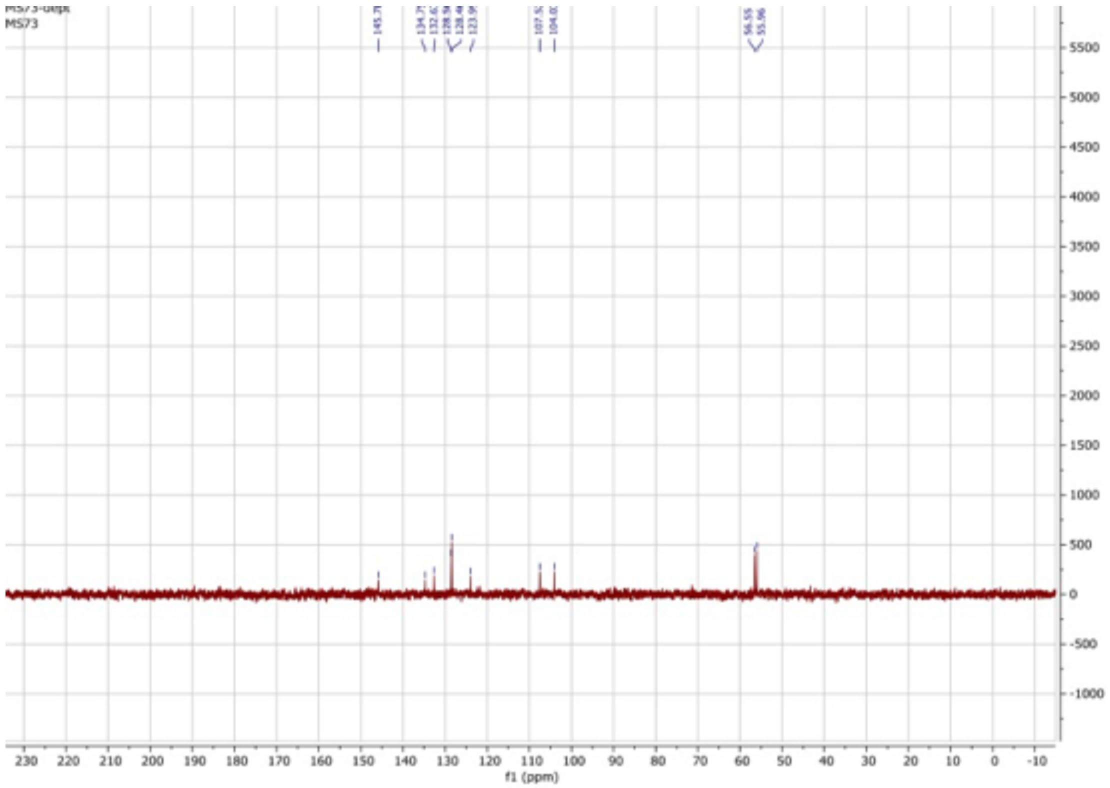

^{13}C NMR (101 MHz, Chloroform-d) δ 145.78, 134.75, 132.63, 128.56, 128.46, 123.99, 107.52, 104.03, 56.55, 55.96.

Area % Report

Data File: C:\32Karat\Projects\Default\Data\delia\Martina\vellutone Z.dat
Method: C:\32Karat\Projects\Default\Method\standard 0.7 ml.met
Acquired: 16/04/2021 8.50.00
Printed: 04/08/2022 13.22.32

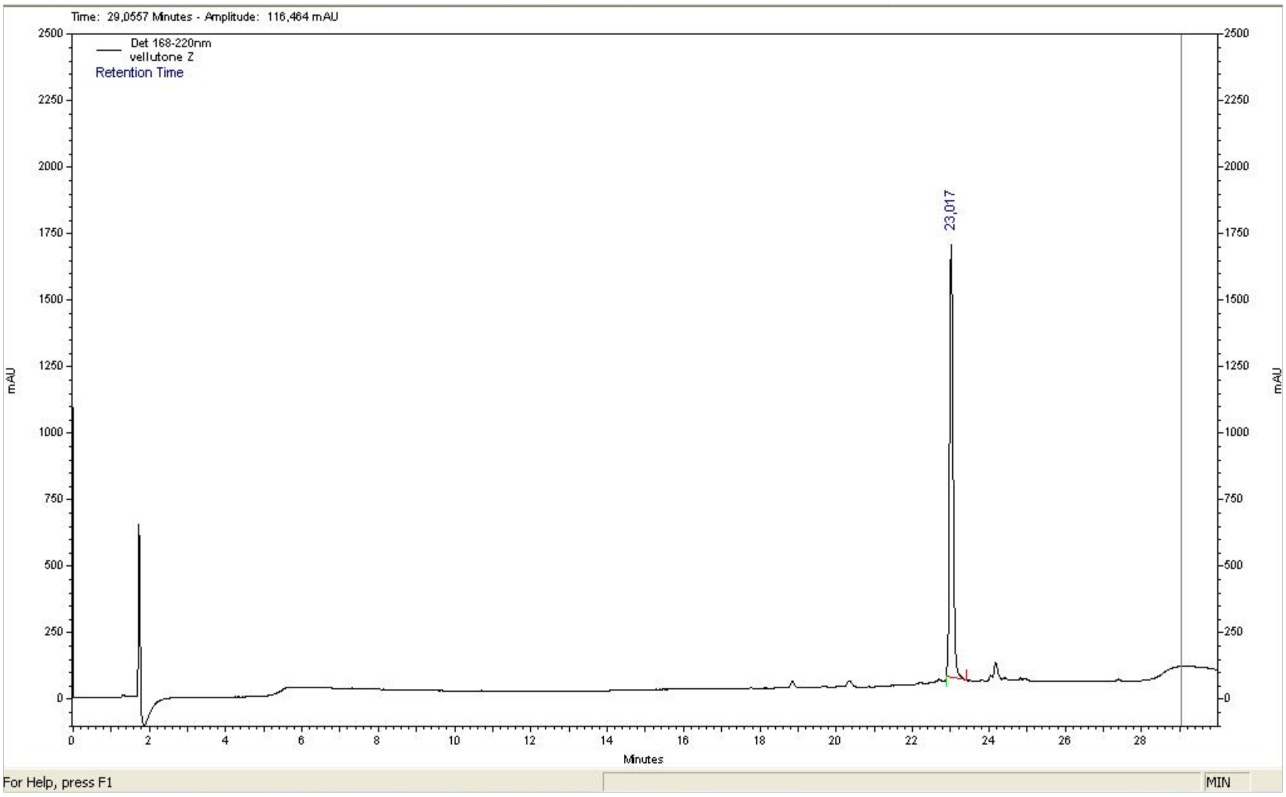

Det 168-220nm
Results

Time	Area	Area %	Height	Height %
23,017	10467135	100,00	1624633	100,00

Totals				
	10467135	100,00	1624633	100,00

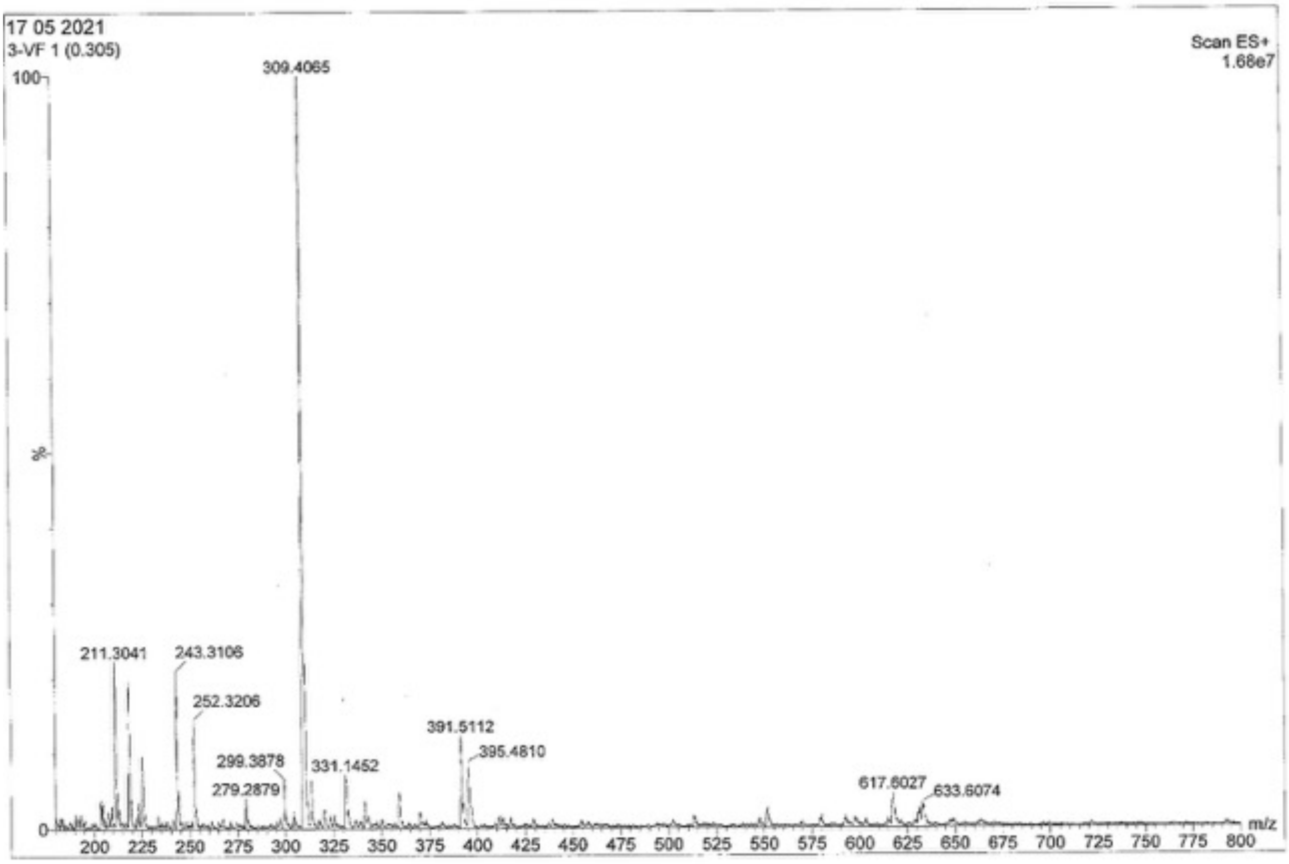
17 05 2021
3-VF 1 (0.305)
Scan ES+
1.68e7
100
%
0
309.4065
211.3041
243.3106
252.3206
299.3878
279.2879
331.1452
391.5112
395.4810
617.6027
633.6074
m/z
200 225 250 275 300 325 350 375 400 425 450 475 500 525 550 575 600 625 650 675 700 725 750 775 800

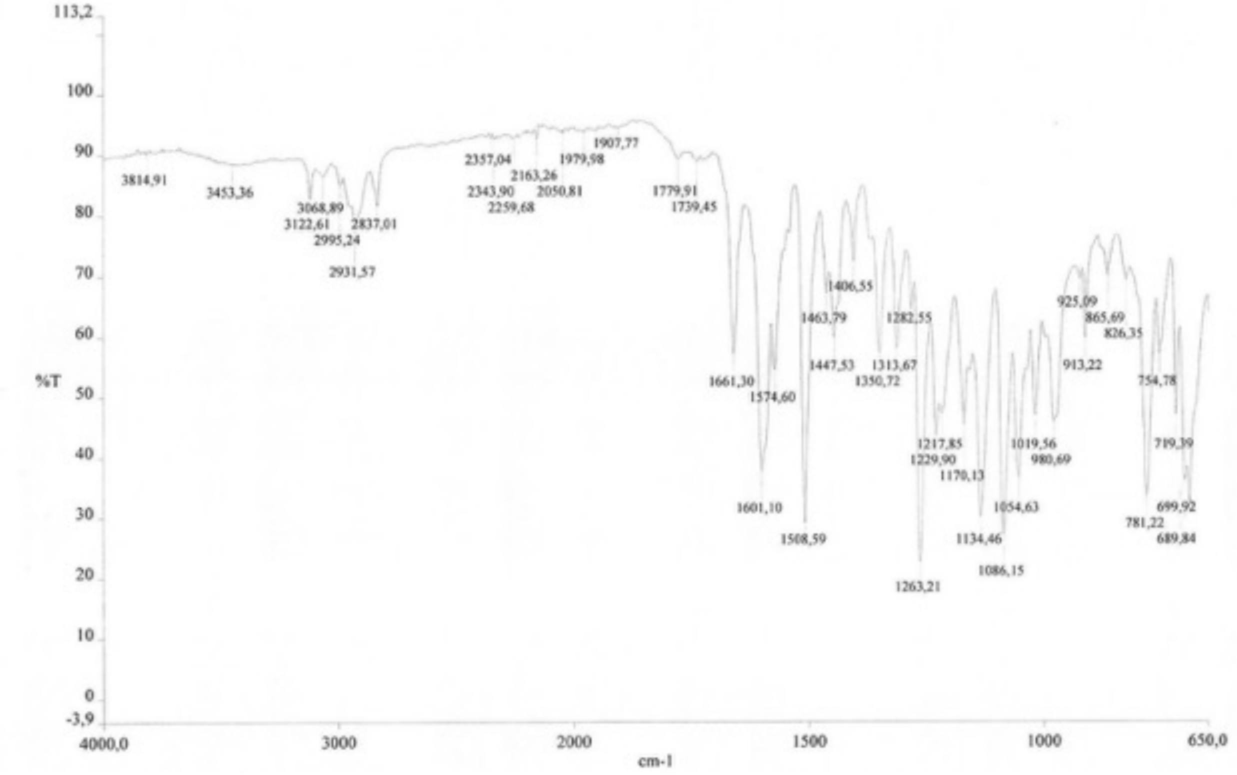
113,2
%T
3814,91
3453,36
3068,89
3122,61
2837,01
2995,24
2931,57
2357,04
2343,90
2163,26
2050,81
2259,68
1979,98
1907,77
1779,91
1739,45
1661,30
1574,60
1601,10
1508,59
1463,79
1447,53
1406,55
1313,67
1350,72
1282,55
1217,85
1229,90
1170,13
1263,21
1134,46
1086,15
1054,63
1019,56
980,69
925,09
913,22
865,69
826,35
754,78
719,39
699,92
781,22
689,84
-3,9
4000,0
3000
2000
1500
1000
650,0
cm-1

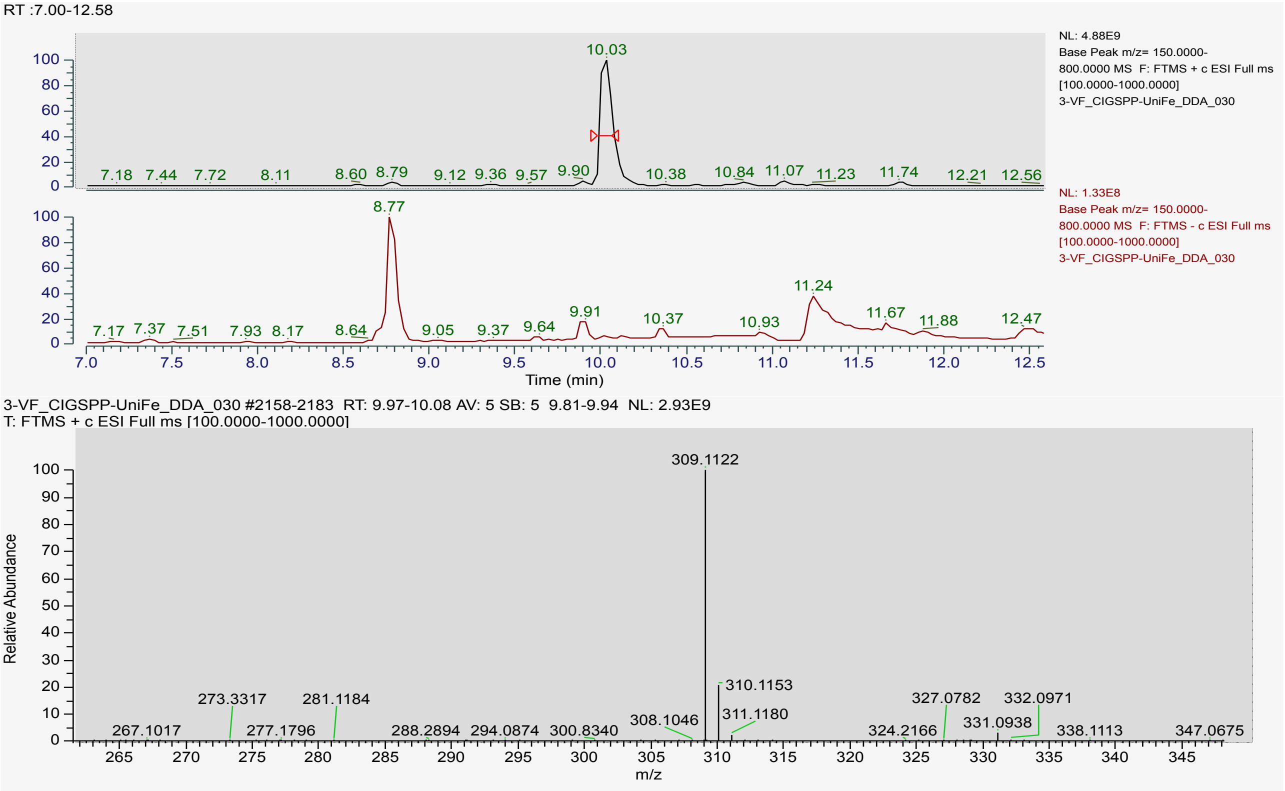

Peak Mass	Display Formula	Combined Fit	RDB	Delta [ppm]	Theo. mass	Rank	Combined Score	# Matched Iso.	# Missed Iso.	MS Cov. [%]	Pattern Cov. [%]
309,1122	$C_{19}H_{17}O_4$	23,7200377676601	11,5	0,2	309,11214	1	95,94	4	4	99,96	98,96
331,0938	$C_{19}H_{16}O_4{}^{23}Na$	15,094285170234	11,5	-0,81	331,09408	5	93,79	2	5	98,16	97,31
